# Assessment of Aggregation- and Condensation-Prone Regions of Proteins Involved in Neurodegenerative, Neurological and Mental-State Diseases

**DOI:** 10.3390/biom16091286

**Published:** 2026-09-05

**Authors:** Katja Venko, Eva Žerovnik

**Affiliations:** 1 Theory Department, National Institute of Chemistry, Hajdrihova 19, 1000 Ljubljana, Slovenia; 2 Department of Biochemistry and Molecular and Structural Biology, Jožef Stefan Institute, Jamova 39, 1000 Ljubljana, Slovenia; 3 Jožef Stefan International Postgraduate School, Jamova 39, 1000 Ljubljana, Slovenia

**Keywords:** protein aggregation, protein condensation, liquid–liquid phase separation—LLPS, amyloid, prion, prediction from amino acid sequence, in silico models

## Abstract

Over more than a decade of development, various data-driven computational approaches based on the physicochemical properties of proteins have been developed for estimating the aggregation potential of proteins. The currently available algorithms and models enable a sequence-based design strategy to predict regions prone to transform into liquid–liquid phase-separated (LLPS) condensed or solidified aggregated states; for the latter, one can distinguish amyloid and prion-like aggregates. In this study, we performed a comprehensive in silico experiment; more than 40 models were used to test amino acid sequences of various proteins known to aggregate in neurodegenerative diseases, certain progressive myoclonic epilepsies and mental illnesses. Altogether, 20 proteins were analyzed and their proneness to aggregate or condensate was discussed in view of their possible normal and/or toxic function. The reported large set of computational models enables highly accurate predictions of proteins that are prone to aggregation and/or condensation. Furthermore, such aggregation profiling can be performed for any protein sequence of interest.

## 1. Introduction

Protein aggregation into amyloid or prion states is a feature of almost all neurodegenerative diseases, such as Alzheimer’s disease (AD), Parkinson’s disease (PD), Huntington’s disease and amyotrophic lateral sclerosis (ALS). Most neurodegenerative diseases can also be considered as proteinopathies, characterized by aggregation of one or two specific proteins. Numerous studies have shown the co-occurrence of protein aggregates that are not primarily involved in the pathological process of each neurodegenerative disease. These so-called secondary protein aggregates influence the propagation of an individual’s pathology [1]. Furthermore, the scope of protein aggregation as a possible primary or secondary effect has been expanded to mental health illnesses such as schizophrenia, bipolar disorder and major depression [2,3,4]. This indicates that some functions and signaling pathways may be shared by the aggregating proteins causing neurological and mental-state disorders [5,6,7]. Therefore, common underlying mechanisms linked to protein aggregation may apply, at least for some general processes leading to neurodegeneration or initially to a decline in cognitive performance—the so-called mild cognitive impairment (MCI) [8]. What is even more interesting, and in line with the brain–gut axis, is that other diseases, such as diabetes mellitus type 2 and cardiovascular disease, also share some of these common underlying mechanisms [9,10,11,12]. Not least in viral infections, protein condensation also occurs in the brain of the human host [13], possibly leading to “brain fog” and states similar to long COVID [14]. Thus, the role of protein aggregation becomes broader. It could well be that in certain diseases, especially monogenic inherited cases, protein aggregation is the initial cause of pathology, followed by increased oxidative stress and neuroinflammation. If protein aggregation occurs as a consequence, it is also important as it amplifies other mentioned processes in a kind of negative feedback loop. Function-wise, much remains open to research. With current knowledge collected from Gene Cards, STRING analysis of protein–protein networks and similar bioinformatics tools, we can see overlapping processes in which proteins aggregating in different neurodegenerative and mental health disorders participate, among them autophagy with mTOR signaling, cytoskeleton dynamics, and neuronal plasticity via brain-derived neurotrophic factor (BDNF).

In this regard, our study focused on aggregating proteins known to be related to different proteinopathies, such as neurodegenerative diseases with AD, PD and ALS as examples, but also progressive myoclonic epilepsies (PMEs) and mental illnesses. The proteins analyzed are cystatins (stefin A and stefin B, cystatin C) [15,16,17,18], α-synuclein [19], SOD1 (superoxide dismutase 1) [20], malin (E3 ubiquitin-protein ligase NHLRC1) [21], laforin (lafora PTPase) [21], RIP3 (receptor-interacting protein kinase 3) [22], CRMP1 (collapsin response element mediator protein 1) [23,24], TRIOBP1 (trio and F-actin binding protein, splice 1) [25], FUS (fused in sarcoma) [26], tau [27], TDP-43 (TAR DNA-binding protein 43) [28], DISC1 (disrupted in schizophrenia 1) [29], FABP3 (fatty acid-binding protein 3) [30], LIMP2 (lysosome membrane protein 2) [31], dysbindin 1 [32], hnRNPA1 (heterogeneous nuclear ribonucleoprotein A1) [33], NPAS3 (neuronal PAS domain-containing protein 3) [34], and PRICKLE1 (prickle-like protein 1) [35]. In general, protein aggregation is triggered by an interplay of particular amino acid sequence regions, protein 3D structure and environmental factors [36]. On this basis, prediction models using various bioinformatics and machine learning approaches have been developed. In the last decade, impressive innovations in computational methods for protein aggregation/condensation prediction have been made. The currently available algorithms and in silico tools enable a sequence-based design strategy to predict the propensities of proteins to transform into liquid–liquid phase-separated (LLPS) condensed states or solidified aggregates of amyloid and prion type [37,38,39]. Thus, by using in silico tools, easy and quick evaluation of aggregation/condensation proneness of various proteins can be performed, which can help us to understand their mechanisms of action and to plan further complex and expensive experimental tests [40]. Protein condensates formed by LLPS in distinction to more toxic forms of protein aggregates are at least initially reversible, but over time and under high-intensity light or high protein concentration they can transform into irreversible amyloid clumps [41].

Moreover, comparative analysis of the proteomes suggests that proteins forming membraneless organelles (MLOs) have a high propensity to phase-separate. It was shown that in MLOs the content of intrinsically disordered proteins and proteins that serve as LLPS drivers increases, whereas the content of proteins prone to aggregation decreases [42]. However, soluble prefibrillar oligomers, rich in beta structure, are still the most toxic to cells, likely by perforating membranes or by interacting with membrane-bound receptors [43,44]. Therefore, predicting the aggregation and condensation properties of proteins may reveal the unknown mechanisms of their toxicity. Aggregate-prone regions may well represent sites for small-molecule inhibitors of aggregation, condensation and membrane damage.

In this study, a set of 20 above-named proteins, which have been reported to aggregate in neurodegenerative diseases, progressive myoclonic epilepsies (PMEs) and mental illnesses, was chosen for the in silico experiment. We performed computational analysis from their protein sequences by using more than 40 open-source programs. We analyzed aggregation/condensation-prone regions and compared their proneness to aggregate or condensate with available published in vitro data.

### Biological Functions of the 20 Proteins Involved in Neurodegenerative Diseases (AD, PD and ALS), PMEs and Mental-State Illnesses (Schizophrenia and Major Depression)

Protein aggregation is a hallmark of several neurodegenerative disorders and is increasingly recognized as a relevant feature of selected psychiatric and neurodevelopmental conditions. Considering the specific functions of aggregate-prone proteins, we should be aware that misfolded or aggregated proteins gain in toxic functions and at the same time they are losing their normal functions. Although the proteins involved considerably differ in their primary structures and physiological functions, their aggregation may converge on common cellular processes. Therefore, some physiological functions and signaling pathways may be shared by aggregating proteins that cause neurological and mental problems [5,45]. The pathologies and normal functions of the selected proteins that are important for brain physiology are described below.

One of the most-characterized aggregation-prone proteins is α-synuclein, encoded by the *SNCA* gene. In PD α-synuclein forms aggregates and together with other co-aggregating proteins it forms neuronal inclusions known as Lewy bodies [19]. It is a natively unfolded protein, also termed an intrinsically disordered protein. The protein is composed of three distinct regions: (1) an amino terminus (residues 1–60), containing apolipoprotein lipid-binding motifs, which are predicted to form amphiphilic helices conferring the propensity to form α-helical structures on membrane binding; (2) a central hydrophobic region (61–95), the so-called NAC (non-Aβ component), which confers the β-sheet potential; and (3) a carboxyl terminus that is highly negatively charged, and is largely disordered.

Protein aggregation also occurs in ALS. SOD1, a homodimeric metalloenzyme that catalyzes the removal of superoxide radicals, is one of the proteins implicated in familial and sporadic forms of ALS. Mutations A4V and G93A cause familial types of ALS, whereas most cases are sporadic, due to wild-type SOD1 aggregation (and loss of function) [20]. A metalloenzyme complex of SOD1 localizes to the cytoplasm, nucleus, and mitochondria. Interestingly, some studies indicate an interaction between cystatin B and SOD1 [46].

Members of the cystatin family are likewise implicated in some neurodegenerative and epileptic conditions characterized by protein aggregation. Cystatin C, the L68Q mutant, aggregates in hereditary Cystatin C amyloid angiopathy (HCCAA) affecting brain blood vessels. In contrast, mutations in cystatin B (stefin B) lead to the progressive myoclonus epilepsy of type 1 (EPM1), also known as Unverricht–Lundborg disease [16,17,47]. Cystatins are primarily inhibitors of cysteine proteases and thereby contribute to the regulation of proteolysis and modulation of immune responses. Alternative functions of cystatins are also important, such as induction of autophagy and maintenance of the integrity of actin cytoskeleton. Cystatin A (stefin A) is an intracellular thiol proteinase inhibitor with an important role in desmosome-mediated cell–cell adhesion in the epidermis [48]. Cystatin B (stefin B) appears to have several functions in the nervous system. It participates in neural proliferation, interneuron migration, neurogenesis and physiological processes at the synapse, including synaptic plasticity [49,50]. In addition, it contributes to prevention of inflammation and oxidative stress. It inhibits NLRP3 inflammasome activation via AMPK/mTOR signaling [51]. Accordingly, stefin B-deficient macrophages led to increased caspase-11 expression and NLRP3 inflammasome activation, destabilization of the mitochondrial membrane and elevated mitochondrial ROS generation [51].

Interestingly, very similar symptoms to EPM1 caused by mutations of stefin B also appear in the case of PRICKLE and *SCARB2* gene mutations. PRICKLE family proteins participate in planar cell polarity (PCP) signaling [35]. PRICKLE1 binds to the transmembrane protein Vangl, and Vangl–Prickle complexes accumulate at the plasma membrane, where they contribute to regulation of the actin cytoskeleton [52] and inhibit a cytoplasmic protein Dishevelled and transmembrane protein Frizzled [35]. While the *SCARB2* gene encodes the LIMP2 protein, which functions in membrane transport in the endosomal/lysosomal compartment [31]. LIMP2 is also present at cardiac intercalated discs, where it interacts with N-cadherin. The knockdown of LIMP2 with RNA interference decreased the binding of N-cadherin to the phosphorylated form of beta-catenin and LIMP-2 overexpression had the reverse effect [53].

Alterations in protein degradation and cellular homeostasis are also prominent in Lafora disease, a type of PME [21], characterized by the accumulation of starch-like polyglucosan inclusions known as Lafora bodies. These inclusions are particularly abundant in organs with high glucose metabolism, like the brain, heart, liver, and skeletal muscle. Malin and laforin are the major Lafora disease-associated proteins. Both participate in glycogen metabolism; malin exerts ubiquitin ligase activity, while laforin has phosphatase activity. Malin interacts with laforin and promotes its polyubiquitination and degradation. Malin and laforin co-localize in the endoplasmic reticulum, where they form centrosomal aggregates. The centrosomal aggregation of laforin and malin is dependent on the functional microtubule network. Laforin/malin aggregates are immunoreactive for ubiquitin, ubiquitin-conjugating enzymes, endoplasmatic reticulum chaperones and proteasome subunits, demonstrating their aggresome-like properties.

RIP3 has been implicated to mediate necroptosis in AD, ALS, PD, Gaucher’s disease and multiple sclerosis [22]. RIP3 functions as a serine/threonine protein kinase and forms a necrosis-inducing complex with other proteins, which initiate regulated cell death [54]. On the other hand, RNA-binding proteins TDP-43 and FUS are also implicated in neurodegenerative diseases, like ALS and frontotemporal dementia [26,28]. Both proteins regulate multiple aspects of RNA metabolism. Moreover, FUS additionally participates in nuclear–cytoplasmic shuttling [26], while TDP-43 links to regulation of α-synuclein gene expression [27].

Importantly, abnormal protein aggregation is not restricted only to classical neurodegenerative diseases. In this regard, DISC1 has been reported to form aggregates in schizophrenia, bipolar disorder, and major depressive disorder [29,55]. DISC1 is involved in a broad range of cellular processes, including neurotransmitter signaling and NMDAR expression and signaling through a phosphodiesterase 4/PKA/CREB-dependent mechanism, which provides a potential molecular basis for its influence on NMDAR-dependent cognitive processes. Phosphorylated CREB binds to a specific sequence in the promoter region of BDNF and thus regulates its transcription. Genome-wide association studies (GWAS) indicate that genes involved in the cAMP/PKA/CREB signaling pathway could be potential candidates for schizophrenia [56].

In patients with schizophrenia, CRMP1 aggregates were also reported. CRMP1 can form co-aggregates with DISC1 [24]. CRMP family of proteins is a class of microtubule-associated proteins that play important roles in the process of nervous system development, such as axon guidance, synapse maturation, cell migration [23]. CRMP1 is a cytosolic phosphoprotein originally identified as a mediator of semaphorin 3A signaling during axon differentiation.

Another protein implicated in schizophrenia and schizoaffective disorder is NPAS3. Its aggregation was demonstrated in patients [34]. NPAS3 is a brain-expressed transcription factor whose loss of function is associated with neurodevelopmental disorders [57]. It regulates expression of nerve growth factor VGF through the NF-κB signaling pathway. An overexpression of NPAS3 promotes neural cell proliferation, which can be blocked by knockdown of VGF. The downstream signaling pathways involved in NPAS3-VGF-induced proliferation include the glutamate NMDAR receptors and regulatory network involving NPAS3, NF-κB, and BDNF [57].

Furthermore, defects in dysbindin 1, a *DTNBP1* gene encoded protein, are also associated with schizophrenia. Genetic mutations lead to alterations in the glutamatergic transmission in the brain and modify Akt signaling. In schizophrenic patients reduced protein levels have been observed in nerve terminals of the hippocampus and associated with an increased release of glutamate [58]. Dysbindin 1 is co-assembled with seven other proteins into a large protein complex, known as the biogenesis of lysosome-related organelles complex-1 (BLOC-1). BLOC-1 and AP-3 complex are required to target membrane protein cargos into vesicles assembled at cell bodies for delivery into neurites and nerve terminals [59]. Large dense-core vesicles (LDCVs) are involved in the secretion of hormones and neuropeptides, including BDNF, which plays important roles in neuronal development, survival and synaptic plasticity [32].

Additional proteins indicating aggregation properties in neurological and mental-state diseases are hnRNAP1 and TRIOBP1. Mutations in the hnRNAP1 gene occur in ALS and inclusion body myopathy with early-onset Paget disease with frontotemporal dementia [33]. hnRNAP1 participates in packaging pre-mRNA into hnRNP particles, transport of poly(A) mRNA from the nucleus to the cytoplasm and modulation of splice site selection [60], while TRIOBP1 aggregates in individuals with schizophrenia and major depressive disorder [25]. It is a ubiquitous protein involved in stabilization of the actin cytoskeleton [61]. In addition, FABP3 has emerged as an important contributor to PD pathogenesis [30]. FABPs are cytosolic proteins involved in fatty acid (FA) uptake, transport and targeting. Through their interactions with lipids, they can modulate the function of membrane-embedded enzymes, stability of membranes, ion channels and receptors, and consequently gene expression and cellular growth and differentiation. In the central nervous system, FABP3 impacts α-synuclein aggregation, neurotoxicity, and neuroinflammation. Accumulation in mitochondria during neurodegeneration, disrupting membrane potential and homeostasis, is reported.

On the other hand, tau, encoded by the *MAPT* gene, represents another major aggregation-prone protein and is implicated in many neurodegenerative diseases collectively known as tauopathies [27]. Tau functions in binding and stabilizing the microtubules, internal cytoskeleton and tracking routes of neurons. Multiple tau isoforms exist in the nervous system and their new functions are recently being discovered [62].

Taken together, the physiological functions and disease associations of the 20 aggregation-prone proteins considered in this study reveal some convergence among proteins that, at first sight, appear functionally unrelated. In Table 1 the normal physiological functions of selected proteins and the brain diseases they contribute to or cause, are summarized. It can be observed that many of the proteins aggregating in mental illnesses are involved in actin cytoskeleton organization: TRIOBP1 regulates actin filament stability [61] while CRMP1 and tau are involved in microtubule stability and dynamics [23,27]. Stefin B also has a role in cytoskeleton dynamics by forming a complex with cytoskeletal proteins β-spectrin and NF-L (neurofilament light chain) [16]. In parallel, stefin B, malin, and laforin play roles in protein degradation and cellular homeostasis [17,21]. Stefin B and α-synuclein are important for synaptic plasticity via BDNF [16,19]. TDP-43, FUS and hnRNPA1 are RNA-binding proteins that regulate RNA metabolism [26,28,60]. Thus, convergence on several major cellular processes are observed: (i) RNA binding and regulation of RNA metabolism, (ii) protein degradation and maintenance of cellular homeostasis, (iii) synaptic plasticity via BDNF, and (iv) involvement in actin cytoskeleton organization.

## 2. Materials and Methods

### 2.1. Dataset

For the computational analysis, a set of 20 human proteins that were reported to aggregate in neurodegenerative diseases, some types of progressive myoclonic epilepsies, or mental illnesses were collected. In general, these are mainly proteins with a known amyloidogenic mode of action. The amino acid sequences of these proteins were compiled from the UniProtKB database (www.uniprot.org). The list of the studied proteins with their UniProtKB codes and information about their functions and associated pathologies is shown in Table 1.

### 2.2. In Silico Experiments

Prediction of protein aggregation/condensation formation was based on amino acid sequence input (Supplementary Figures S1a–S20a). Models using various bioinformatics and machine learning approaches were applied to calculate protein proneness and/or determine active regions. We performed computational analysis from the protein sequences by using >40 open-source tools. The tools used in this study are listed in Table 2. We analyze propensities for forming condensates, aggregates, amyloids and prions. In general, regions were identified and represented if at least five or more amino acids were predicted as aggregation/condensation hotspots. The propensity can be evaluated with all tools, while some tools also identify condensation-, aggregation-, amyloid- and/or prion-prone regions. In Supplementary Table S2, further details about the versions and setting of tools used in this study are available.

### 2.3. 3D Visualization of Aggregation/Condensation-Prone Regions

Most algorithms for detecting aggregation-prone regions (APRs) and condensation-prone regions (CPRs) from primary protein sequences perform predictions without considering the 3D structure of the protein. Consequently, aggregation or condensation-prone regions can also be predicted in the inner core of the protein, which in general would not contribute to aggregation unless the protein unfolded extensively. The main bottleneck for detecting APRs/CPRs at protein surfaces is the lack of known high-resolution structures that can be incorporated into the algorithms. Only a few predictors combine structure-based and sequence-based features into integrated predictions, which results in improved accuracy of predictions [40]. Thus, we visualized predicted APRs and CPRs in 3D structures of selected proteins based on the results of predictions presented in Table 3 and Figures S1a–S20a. Protein structures were obtained from the RCSB Protein Data Bank (www.rcsb.org) or, if no high-resolution structure was available in PDB, the predicted structure from the AlphaFold Protein Structure Database was used (alphafold.ebi.ac.uk) [105]. The results were visualized with UCSF ChimeraX 1.9 [106]. Criteria for visualization of CRPs were (i) cyan—region determined with 2 predictors and (ii) blue—region determined with 3 or more predictors. Criteria for visualization of ARPs were: (i) light green—region determined with 2 predictors and (ii) dark green—region determined with 3 or more predictors. Criteria for visualization of amyloid regions were: (i) yellow—region determined with 3–5 predictors and (ii) orange region determined with 6 or more predictors.

## 3. Results

**Figure 1:**
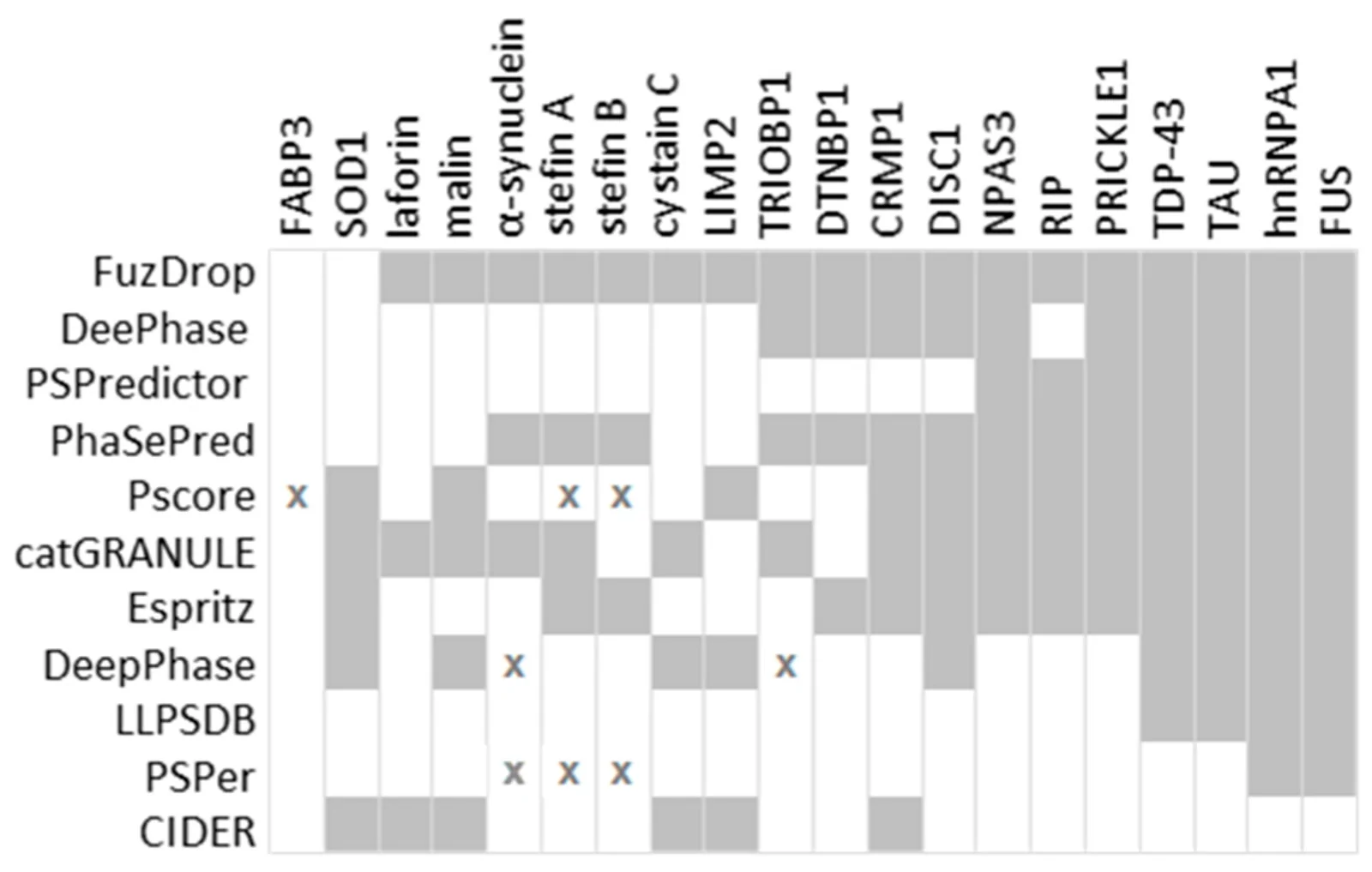
Heatmap of protein condensation propensities; *gray*—condensate, *white*—no condensate, *X*—not available.

**Figure 2:**
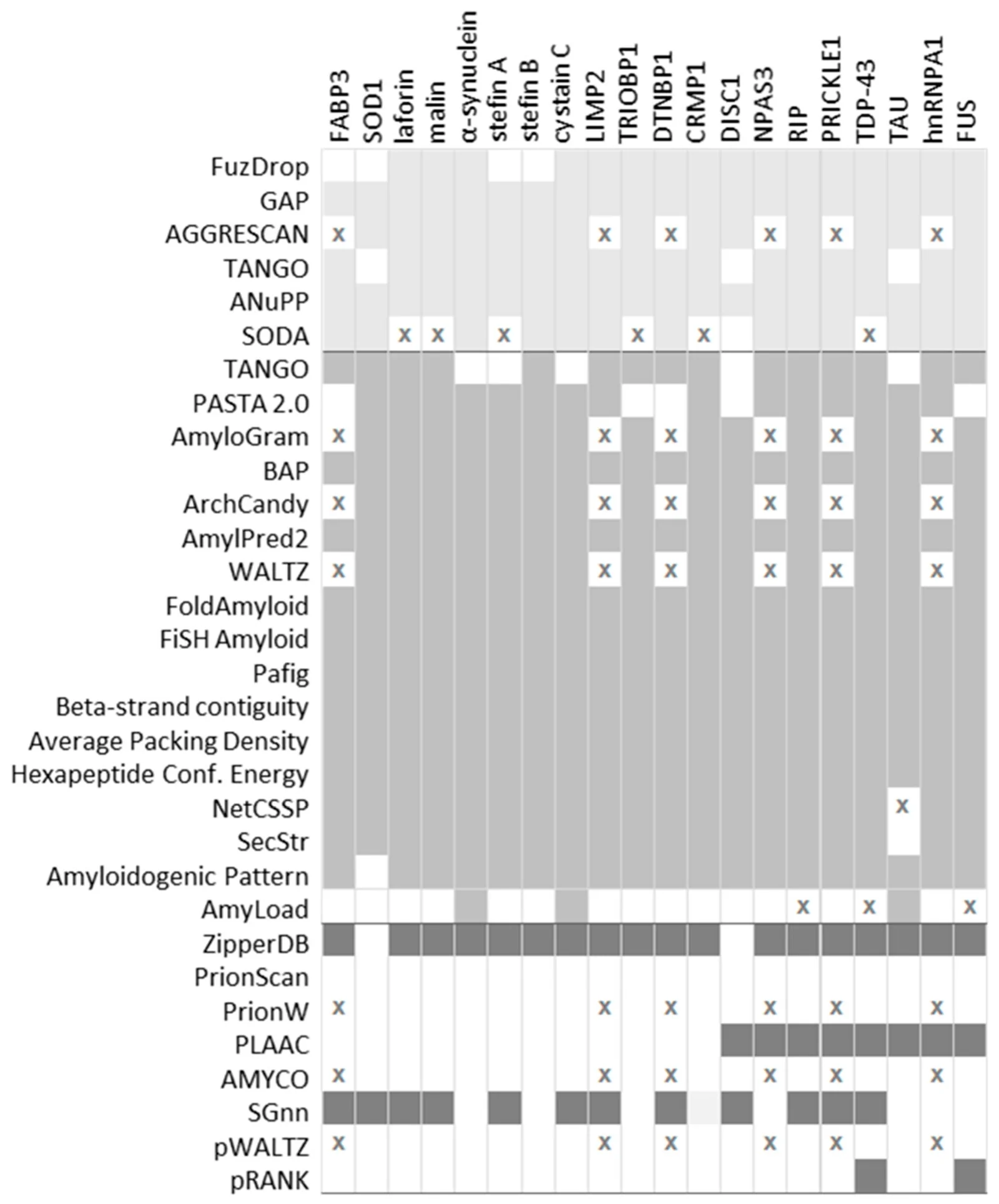
Heatmap of protein aggregation propensities; *light gray*—aggregate, *middle gray*—amyloid, *dark gray*—prion, *white*—negative prediction, *X*—not available.

Analysis of predictions generated by 44 programs (Figure 1 and Figure 2) reveals that all 20 proteins have aggregation/amyloid propensity, whereas no overall prion propensity was detected. Strong condensation propensity was predicted for FUS, tau, hnRNPA1 and TDP-43, moderate condensation propensity for PRICKLE1, NPAS3, DISC1, RIP and CRMP1, while the remaining proteins showed comparatively weak condensation propensity, with more than five predictors classifying them as non-condensation-prone. These findings are broadly consistent with our expectations, partially based on the literature data. All selected proteins were expected to exhibit the potential to form aggregates and no potential to form the prion type of aggregates, whereas only a subset was anticipated to undergo biomolecular condensation. Overall, predictors for aggregation/amyloid proneness indicate relatively strong agreement among predictions; on the other hand, predictions for condensation and prion propensity are considerably more heterogeneous and contradictory.

Among the used predictors, not all of them provide residue-level predictions of aggregation/condensation-prone regions (APRs, CPRs). Detailed analyses were therefore restricted to tools capable of identifying such sequence segments. These analyses included five predictors for condensates, five for aggregates, 14 for amyloids and two for prions. Predicted regions were compared across individual tools. In general, the results supported the overall classification of condensation/aggregation propensities, although substantial variability was observed in the precise localization of predicted CRPs and APRs. Detailed residue-level predictions are presented in Figures S1a–S20a and Table S3. Table 3 and Table S1 summarize the consensus predictions for condensation-, aggregation-, amyloid- and prion-prone regions according to the number of predictors supporting each region. The complete results of predictions obtained with all predictors are provided in Table S3. To further integrate structure- and sequence-based features, the predicted consensus APRs and CPRs were mapped onto 3D protein structures. This combined representation facilitates interpretation of the predictions in their structural context and may improve the biological relevance of the identified regions. The corresponding 3D visualizations are presented in Figures S1b–S20b.

The proteins included in this study cover a broad range of molecular sizes and can be broadly divided into small, medium-sized and large proteins. The limit is arbitrary, proteins with a molecular mass > 50 kDa are classified as large. Small globular proteins (<20 kDa) include stefin A (11 kDa) and stefin B (11.1 kDa), cystatin C (13.3 kDa), α-synuclein (14.5 kDa), FABP3 (14.8 kDa) and SOD1 (15.9 kDa). Their oligomers have been characterized by biophysical techniques. For example, a tetramer of human stefin B has been studied by both heteronuclear NMR and X-ray diffraction and it is composed from two domain-swapped dimers [107]. The structure of domain-swapped dimers and even the octamers of cystatin C have been determined as well [108,109]. In accordance, non-native SOD1 trimers have been reported to represent particularly toxic oligomeric species [110]. On the other spectrum of the set of proteins are medium-sized proteins (20–50 kDa), such as laforin (37 kDa), malin (42 kDa), hnRNPA1 (39 k Da), dysbinidin 1 (39 kDa), TDP-43 (43 kDa), and even some larger proteins (>50 kDa), such as FUS (53 kDa), LIMP2 (54 kDa), RIP3 (57 kDa), CRMP1 (62 kDa), TRIOBP1 (68 kDa), tau (79 kDa), DISC1 (94 kDa), PRICKLE1 (94 kDa) and NPAS3 (101 kDa).

### Comparisons of Computational and Experimental Studies

Notably, some interesting matches among our computational predictions and reported experimental observations were found. Overall, the in silico analyses identified several hotspot regions of aggregation/condensation although only a subset of these regions has thus far been experimentally characterized. Consequently, the predicted regions may provide useful starting points for future experimental studies and may help address unresolved questions concerning protein aggregation in proteinopathies.

Stefin B, for example, was shown to interact with brain β-spectrin, a cytoskeletal protein with neurofilament light (NF-L) chain, which plays an important role in cytoskeleton dynamics and receptor for activated C kinase 1 (RACK-1). These proteins co-localize in Purkinje cells and Bergmann glia, where they form a complex with stefin B and contribute to cell development and differentiation [111]. The strong aggregation and condensation propensity of this member of cystatins was observed also through in silico experiment. Notably, HCCAA is caused by L68Q mutation leading to aggregation of the cystatin C [18]. In our analysis, the wild-type region surrounding residue L68 is not determined as an aggregation/condensation-prone region, with the exception of predictions obtained using AmyLoad and ArchCandy. The aggregation has also been experimentally observed for residues 84–90 and 124–129 [89], which was also determined computationally.

SOD1 is a compact homodimer protein with a β-barrel structure stabilized by a C57–C146 disulfide bridge. Its metal-binding site involves residues H46, H48, H63, H71, H80, D83, and H120. SOD1 tends to form fibrillar aggregates in the absence of the intramolecular disulfide bond or bound zinc ions [112]. These aggregates may have cytotoxic effects in ALS. Our analysis identified aggregation and amyloid-prone regions at residues 4–8, 15–22, 30–35, 44–50, 101–107, 112–120, and 146–152. These regions are also consistent with experimental observations relevant to familial types of ALS, which are frequently associated with pathological SOD1 mutations (A4V and G93A) [113]. Moreover, in residues 101–107, 113–120, and 147–153, the amyloid aggregates were observed [112]. Interestingly, the tryptophan residue at position 32 (W32) is necessary for the formation of a competent seed for aggregation, since the W32S substitution blocked this phenomenon. However, from over 185 SOD1 mutations identified to date, only some (H46R, D90A, and R115G) cause ALS with a defined clinical phenotype. Thus, it is believed that the pathogenicity of SOD1 mutations is not due to a lack of functional protein but rather to the accumulation of its misfolded aggregates [20]. Moreover, some studies indicate interaction between cystatin B and SOD1 [46], which can also be supported with our predictions, since aggregation/condensation-prone regions were determined in both proteins.

α-synuclein fibrillar aggregates are characteristic of several neurodegenerative diseases collectively referred to as synucleinopathies [19,114,115]. α-synuclein is well known to be involved in fibril formation via Alzheimer’s disease amyloid plaque domain (NAC domain); this is the central hydrophobic region, which forms the core of the filaments, yet the C-terminus may regulate aggregation and determine the diameter of the filaments [116]. Our analysis identified high supported aggregation and condensation propensities with most reliable APRs at residues 14–19, 36–42, 55–63, and 73–80 and broad CPR spanning residues 25–128. Residues 35–40 have been previously experimentally demonstrated to form amyloid fibrils [89], consistent with our predictions. C-terminal region is largely unstructured and highly negatively charged [116] and known to interact with tau and TDP-43, potentially promoting the formation of toxic aggregates [117,118]. The condensation proneness in the C-terminal region was also weakly supported by some of the in silico predictors (DeePhase, catGranule, SODA). In this regard, with our computational methods, potential for condensation/aggregation in the C-terminal region of tau was also predicted.

The formation of insoluble filaments that accumulate in AD and related tauopathies were reported for tau forms [27]. Experimentally characterized amyloid aggregates include residues 306–378 [119], 591–596 and 623–628 [89]. Tau protein possesses folded the N-terminal domain (two RNA Recognition Motifs—RRM1 and RRM2), and the mostly unstructured C-terminal domain where most disease-associated mutations occur. The 3D high-resolution structure is not known and the Alphafold-2 model also contains regions of low structural confidence. On the C-terminus, the CPRs were computationally predicted, whereas APRs were identified more broadly across all the sequence.

TDP-43 amyloid aggregates have been experimentally observed within the C-terminal region, including residues 312–321 and 335–340 [120]. Due to its functions in regulation of RNA processing, it is well documented to bind to specific intronic regions of genes like CFTR and apoA-II with the N-terminal region [118], while the C-terminal region was already mentioned to interact with α-synuclein [28]. TDP-43 undergoes RNA-dependent LLPS via its C-terminal prion-like domain, forming nuclear and stress-induced condensates. RNA binding stabilizes liquid-like states, whereas reduced RNA binding promotes solidification [121]. Disease-associated mutations increase β-sheet propensity and accelerate liquid-to-solid transition within condensates M337V, A315T, and Q331K at the C-terminus [120]. Pathological TDP-43 aggregates may therefore arise through liquid-to-solid transitions within the condensates. Consistent with these observations, our computational analysis identified a prominent CPR at the C-terminal region (270–414).

Malin and laforin are associated with Lafora disease, a PME. They can form aggregates with aggresome-like properties and are also associated with the centrosome, a membraneless organelle typical for condensates [21]. Our computational analysis indicates strong aggregation proneness, but comparatively weak condensation propensity for both proteins.

CRMP1 is regionally intrinsically disordered and prone to form condensates by surface residues via a disordered region particularly within the N-terminal region. In contrast, ARPs are frequently predicted throughout the whole protein sequence. CRMP1 has been reported to aggregate in patients with schizophrenia and co-aggregates with DISC1 [56,122]. Ravindran et al. demonstrated that the mutant CRMP1 variants can exert dominant-negative effects on oligomerization of CRMP1 proteins. Altered CRMP1 levels have been reported in psychiatric diseases and an enrichment of CRMP1 in insoluble fractions from schizophrenia brain samples has been reported [23]. These findings emphasize that mutations in the CRMP1 gene have a key role in brain development and function, linking it directly to a human neurodevelopmental disorder [24].

TRIOBP1 is a predominantly disordered protein with poorly determined 3D structure [61]. TRIOBP1 insoluble aggregates were found in patients with schizophrenia and major depressive disorder [123]. So far, one critical region at residues 324–348 was elucidated to be required for its aggregation [123]. Zaharija et al. further showed that two distinct regions of the protein are both required for aggregation [25]. Unexpectedly, this region was only predicted by FuzzDrop in our analysis, whereas several other regions of TRIOBP1 received stronger computational support to be capable of inducing aggregation.

RIP3 forms RIP1/RIP3 condensates both in vitro and in cells. These heterodimeric filamentous structures exhibit classical characteristics of β-amyloids [124]. Because of the RIP3 mode of action, the condensation and aggregation propensities are logically to be expected. Residues 457–462 have previously been reported to form aggregates [125]. Our analysis identified ARPs within the C-terminal region, including segments around residues 449–454 and 461–466, but additionally also in several regions throughout the protein.

FUS associates with RNA and other macromolecules to form distinct, reversible phase-separated liquid droplets. Persistence of the phase-separated state and increased cytoplasmic localization have both been proposed to predispose FUS to irreversible aggregation, which is a pathological hallmark of subtypes of ALS and frontotemporal dementia [126]. Cytoplasmic FUS aggregates contribute to motor neuron degeneration. A recent study found that FUS aggregates spread like prions within the brain, leading to detrimental effects on cognitive function and behavior. This prion-like mechanism suggests that misfolded FUS aggregates “corrupt” healthy FUS proteins as a template, contributing to disease progression. The phosphorylation of FUS’s prion-like domain suppresses phase separation and leads to toxic aggregation [26]. The strong condensation propensities were determined with a majority of our predictors, while prion proneness was supported with lower number of predictors (pRANK, PLAAC and ZipperDB). Prion-prone regions were detected computationally at residues 49–53, 81–88, and 112–119. In the C–terminal region a strong aggregation proneness was observed computationally and experimentally, residues 306–311 have been reported to form aggregates (Waltz DB 2.0) and amyloid fibrils [127].

The C-terminal region of DISC1 exhibits considerable structural plasticity, which is essential for its function but at the same time this region is also prone to aggregation. Disease-associated mutations (S713E, S704C, L807-frameshift) are susceptible to transitioning from functional to aggregated states. Oligomer assembly of the C-terminal DISC1 domain (640–854) is regulated by self-association motifs and by the disease-associated polymorphism S704C [29]. Aggregation of DISC1 occurs through a central region of the protein [122]. The DISC1 C-region can form amyloid-like fibers in vitro at residues 716–761 [29], which surprisingly are not defined in our computational studies, but shorter aggregation- and amyloid-prone regions are identified throughout the protein. Structural mapping has divided human DISC1 into four relatively structured regions: D = 257–383, I = 539–655, S = 635–738, and C = 691–836. None of the structured DISC1 regions (D, I, S, or C) formed aggregates on their own in neuroblastoma cells, but the combination of D + I + the linker between them did form visible aggregates [55,122]. DISC1 has also been shown to co-aggregate with TDP-43 [28]. Our computational analyses indicate few CPRs, although these remain largely unsupported by direct experimental evidence. DISC1 has additionally been reported to exhibit prion-like aggregation behavior, although a clearly established prion-like domain has not yet been defined [128]. Consistent with this uncertainty, our computational studies indicate a weak prion potential by PLAAC and SCnn predictors.

FABP3 is a small, highly structured lipid-binding protein. Although FABP3 itself has not been shown to form fibrils, it binds α synuclein and alters its aggregation pathway toward toxic species [129]. Residues 127–129 are annotated for fatty acid-binding. Our computational studies did not identify prominent CPRs, whereas a few aggregation- and amyloid-prone regions were determined. FABP3 promotes prion-like spreading of α-synuclein preformed fibrils in the mouse brain, but FABP3 itself has not been demonstrated to be a prion-forming protein either in vivo or in silico [130].

The experimentally supported aggregation-prone form of dysbinidin 1 is dysbindin-1B isoform (303 aa; which lacks the C-terminal domain) and not the full-length dysbindin-1A [131]. Dysbindin-1B forms perinuclear aggresomes, cell-invasive deposits, and shows exosome-mediated intercellular propagation in mice, which can be described as prion-like behavior [58,132,133]. Our computational study identified CPR at the C-terminus, whereas aggregation-, amyloid- and prion-prone regions were only weakly supported. The co-aggregation of DISC1 and dysbindin has been reported in postmortem brains from a subgroup of chronic mental disease cases. In the same study, the interaction was mapped to DISC1 residues 316–597 and dysbindin 1 residues 82–173 [134].

NPAS3 is also susceptible to aggregation, since structural and cellular studies show mutation-induced (D262V, D314N) loss of fibril reversibility and enhanced aggregation. Mutation V304I increases aggregation propensity in bacterial and mammalian expression systems. Furthermore, the same study also reported that mutated NPAS3-V304I decreases soluble endogenous NPAS3 and increases insoluble endogenous NPAS3, with associated changes in transcriptional activity [135]. Samardžija et al. [34] found full-length NPAS3 in the insoluble fraction in 70% of the samples of the postmortem insular cortex of patients with schizophrenia, indicating that insolubility/aggregation is much more widespread than would be explained by the rare V304I mutation alone. Based on these findings, they concluded that in neuroblastoma cells, oxidative stress had a stronger effect on NPAS3 aggregation than the V304I mutation itself. Our in silico study identified CPR at the C-terminal region and several amyloid-prone regions determined throughout the protein. However, experimental domain studies show that NPAS3 contains an N-terminal basic helix-loop-helix (bHLH) DNA-binding region plus two PAS domains, and that these regions mediate heterodimerization with ARNT/ARNT2, whereas the C-terminal transactivation domain lies outside this core [136].

Mutations in LIMP2, encoded by *SCARB2*, have been identified as a cause of action of myoclonus renal failure and PME [31]. Fourteen disease-causing *SCARB2* mutations have been identified [137] but not identified as causing LIMP2 self-aggregation into detergent-insoluble deposits. To our knowledge, experimentally validated aggregation-, amyloid-, prion- or condensate-prone regions have not yet been reported for LIMP2, although our computational analysis reveals few amyloid-prone regions. The similar lack of experimental or computational validated amyloid-prone regions was observed also for PRICKLE1 [35].

Several studies have identified residues 186–320 as the part of hnRNPA1 that most directly mediates LLPS, reversible amyloid, and pathological fibrillization [138,139,140]. A few segments were identified as reversible amyloid-forming cores in hnRNPA1 (209–217, 234–240) [138], PY-NLS [139], and a 45-residue segment of the prion-like low-complexity domain (LCD) [140]. Guo et al. [141] demonstrated that the wild-type hnRNPA1 can intrinsically assembles into self-seeding fibrils and that the pathological mutations in the prion-like domain strengthen a steric zipper motif, thereby accelerating fibrillization and cross-seeding of wild-type hnRNPA1. Beijer et al. [142] showed that mutations in the LCD/PrLD exert diverse effects: P288A accelerates fibrillization, and G304Nfs*3 impairs LLPS, and alters stress granule disassembly without strongly changing fibrillization. Lee and Rio [143] showed that the D262V LCD mutation alters RNA-dependent interactions, changes stress granule kinetics, and promotes a cell aggregation phenotype. Molliex et al. [144] and Martin et al. [145] showed that hnRNPA1 undergoes spontaneous concentration-dependent LLPS, which is mediated by the C-terminal LCD. Linsenmeier et al. [146] showed that fibrils are promoted specifically at the condensate interface, not uniformly throughout droplets. Morelli et al. [147] further demonstrated that RNA stoichiometry strongly alters the pathway from soluble hnRNPA1A to condensates to amyloid, with low RNA promoting condensation and amyloid, and high RNA suppressing condensation but not necessarily amyloid formation. Consistent with these experimental observations, our computational analyses revealed a CPR in the C-terminus. In contrast, a discrepancy between computational and experimental observations has been noticed, as most strongly predicted amyloid-prone regions were determined primarily within the N-terminal region.

## 4. Discussion

The phenomenon of amyloid toxicity is likely caused by the common structural motifs of the prefibrillar oligomers, which can form an α-helix or β-barrel structure in the membrane environment. The oligomers also interfere with biological membranes rigidity and order, bind with other membrane proteins or even make pores into membranes [42,43]. The “loss of normal function and gain in toxic function” may be in common with many proteinopathies and represents an important therapeutic target. Potential strategies include inhibition of the oligomer formation, stabilization of cellular membranes and inhibition of voltage-gated channels [104]. Therefore, an emerging approach to studying major neurodegenerative and mental illnesses is through studying proteostasis, which consists of regulation protein synthesis, post-translational modifications, folding/misfolding, aggregation and degradation, and clearance of the aggregates. Protein condensation and aggregation are important parts of proteostasis maintenance. In the present study, a set of 20 proteins (Table 1), previously associated with aggregation and/or pathology in neurodegenerative, neurological diseases and major mental-state illnesses, was computationally evaluated for their propensities to form condensates and aggregates, including amyloid- or prion-like assemblies. While proteins such as α-synuclein, tau, and TDP-43 have been extensively investigated for their aggregation potential, many of the remaining proteins have only limited experimental evidence linking them to aggregation or condensation processes. During the design of this study, we considered including negative control to demonstrate that the computational methods do not simply identify ARPs indiscriminately throughout the protein. However, we recognized that identifying truly “non-aggregating” proteins is inherently challenging, as protein aggregation is widely regarded as a general physicochemical property that can emerge under specific environmental or pathological conditions. For example, myoglobulin is a highly stable globular protein, yet it has been shown to aggregate under particular environmental conditions. Consequently, the objective of this study was not to classify proteins as aggregating versus non-aggregating. Instead, we aimed to identify the relative potential of aggregation and condensation propensity and to map protein regions with an elevated potential to undergo these processes. Such an approach is particularly relevant for proteins for which experimental evidence remains scarce or incomplete.

Overall, comprehensive set of tools (more than 40 open-source prediction models) were applied to characterize the aggregation/condensation propensities. Since the main bottleneck for more accurate determination of APRs/CPRs at protein surfaces is the lack of known high-resolution structures, we additionally performed visualization of our consensus predictions with 3D protein structures. This approach enables the integration of structure- and sequence-based features into an integrated prediction. Benchmark comparisons indicated that the computational methods generally performed well in identifying condensation/aggregation propensities, although the precise localization of CPRs and APRs varied substantially among the prediction tools. A major limitation of the present study is the lack of experimental evidence for the investigated proteins, which prevents a direct validation of the predicted CPRs and APRs. Nevertheless, whenever experimental data were available in the literature, we attempted to compare our predictions with these observations and discuss their consistency and potential biological relevance.

We believe that valuable scientific information lies in the application of many (>40) available predictive programs. With this computational approach we confirm that the proteins observed to aggregate or condensate indeed have a high potential to do so. The rational for including multiple predictors lies in the fact that the molecular determinants governing protein aggregation and biomolecular condensation are diverse and multifactorial. Individual computational tools generally assess distinct physicochemical, structural, or sequence-derived features. Therefore, the use of multiple computational predictors and the integration of their outputs provide a significant advantage over reliance on a single method. Most of the tools employed in this study are individual predictors that assess specific molecular characteristics. In contrast, metapredictors (PhaSePred, ANuPP, MetAmyl, AmylPred2) integrate outputs from several computational models to generate a consensus prediction, thereby offering a more comprehensive assessment of protein behavior by integrating approaches and also incorporating further information like intrinsic disorder region integration and other relevant sequence features. A detailed overview of the advantages, limitations, and recent developments of all computational tools used in this study was presented and discussed in our previous work [40], to which readers are referred for a deeper understanding of the methodologies and interpretation of the results reported here. Overall, benchmarking studies conducted by numerous research groups have demonstrated that metapredictors and consensus-based approaches generally exhibit improved predictive performance, robustness, and reliability compared with individual predictors. Through such a comprehensive computational analysis of protein condensation and aggregation propensities, a deeper understanding of aggregation-prone proteins can be achieved. This knowledge may facilitate the rational design of molecules capable of inhibiting biomolecular condensation and/or preventing irreversible amyloid-type aggregation. Similar to the transformative impact of AlphaFold on protein structure prediction, these computational approaches hold considerable promise for the identification of previously unrecognized aggregation-prone proteins within large-scale biological datasets. Future predictive frameworks should additionally incorporate further layers of biologically relevant information, such as metal-binding sites and post-translational modifications (e.g., phosphorylation).

## 5. Conclusions

In this study, set of 20 proteins causing pathology in some brain diseases, characterized as proteinopathies, were computationally tested for their propensities to form aggregates or condensates. More than 40 open sourced prediction models were used to evaluate the aggregation/condensation propensities. Since the main bottleneck for more accurate determination of APRs/CPRs at protein surfaces is the lack of known high-resolution structures, we performed further visualization of our consensus predictions with 3D protein structures to combine structure- and sequence-based features into an integrated prediction. Protein structures were obtained from the Protein Data Bank (PDB) or if no high-resolution structures were available in PDB, AlphaFold Protein Structure Database was used. In this way the results of predictions became more interpretable and applicable. Benchmark analyses were performed and results in general show accurate predictions of condensation/aggregation propensities, but differences in condensation and aggregation prone region determinations. Using proposed in silico model’s platform a highly accurate predictions of condensate and amyloid propensities as well as more general aggregate prone regions can be generated for any protein of scientific or industrial interest. With this study a valuable insight to more understanding of the aggregating proteins is obtained, leading to design of potential inhibitors of condensation or/and amyloid-type of aggregation.

## Figures and Tables

**Table 1:** List of the studied aggregate-prone proteins with their pathological and normal functions.

Protein	UniProtKB	Sequence	Disease	Function
**stefin A** (cystatin A)	P01040	1–98	epidermolytic ichthyosis	intracellular thiol proteinase inhibitor with important role in desmosome-mediated cell–cell adhesion in lower levels of epidermis
**stefin B** (cystatin B)	P04080	1–98	myoclonic epilepsy	reversible inhibitor of cathepsins (L, H, B)
**cystatin C**	P01034	27–146	hereditary cerebral hemorrhage with amyloidosis, age-related macular degeneration, cystatin C amyloid angiopathy (CCAA)	inhibitor of cysteine proteinases
**α-synuclein**	P37840	1–126	Parkinson disease, dementia, multiple system atrophy	roles in synaptic activity (regulation of synaptic vesicle trafficking and subsequent neurotransmitter release), in multimeric membrane-bound state as molecular chaperone, in folding of synaptic fusion components (SNAREs)
**SOD1** (superoxide dismutase 1)	P00441	2–154	amyotrophic lateral sclerosis, spastic tetraplegia, axial hypotonia	destroys radicals (catalyze removal of superoxide ions) and in this way protects cells from formation of other reactive oxygen species (ROS)
**malin** (E3 ubiquitin-protein ligase NHLRC1)	Q6VVB1	1–395	myoclonic epilepsy of Lafora 2 (MELF2)	ubiquitin ligase activity and involved in glycogen metabolism
**laforin** (lafora PTPase)	O95278	1–331	myoclonic epilepsy of Lafora 1 (MELF1)	phosphatase activity and involved in glycogen metabolism
**RIP3** (receptor-interacting protein kinase 3)	Q9Y572	1–518	traumatic brain injury (TBI), multiple sclerosis, Alzheimer’s disease, myocardial ischemia–reperfusion injury	receptor-interacting serine/threonine protein kinase, role in necroptosis, involved in release of inflammatory factors
**CRMP1** (collapsin response element mediator protein 1)	Q14194	1–572	muscular hypotonia, intellectual disability, autism spectrum disorder, amyotrophic lateral sclerosis	cytosolic microtubule-associated phosphoprotein with roles as the mediator of semaphorin 3A signaling involved in axon differentiation during neural development, in remodeling of actin cytoskeleton organization. and synapse maturation
**TRIOBP1** (trio and F-actin binding protein, splice 1)	Q9H2D6	593 (1772–2365)	schizophrenia	ubiquitous protein involved in stabilization of actin cytoskeleton
**FUS** (fused in sarcoma)	P35637	1–526	frontotemporal dementia, amyotrophic lateral sclerosis	DNA/RNA-binding protein with role in various cellular processes such as transcription regulation, RNA splicing, RNA transport, DNA repair and damage response
**tau**	P10636-8	1–758	Alzheimer disease, Pick disease of the brain, progressive supranuclear palsy, frontotemporal dementia, corticobasal degeneration	microtubule-associated protein promotes microtubule assembly and stability
**TDP-43** (TAR DNA-binding protein 43)	Q13148	1–414	amyotrophic lateral sclerosis, frontotemporal dementia, limbic-predominant age-related TDP-43 encephalopathy (LATE)	involved in RNA binding, regulation of RNA metabolism and gene expression
**DISC1** (disrupted in schizophrenia1)	Q9NRI5	1–854	schizophrenia	involved in regulation of embryonic and adult neurogenesis, neural progenitor proliferation
**FABP3** (Fatty Acid-Binding Protein 3)	P05413	1–133	Synucleinopathy	role in intracellular transport of long-chain fatty acids and their acyl-CoA esters
**LIMP2** (lysosome membrane protein 2)	Q14108	1–478	progressive myoclonic epilepsy	lysosomal receptor for glucosylceramidase (GBA1)
dysbindin 1	Q96EV8	1–351	schizophrenia	component of BLOC-1 complex, which is required for normal biogenesis of lysosome-related organelles
**NPAS3** (neuronal PAS domain-containing protein 3)	Q8IXF0	1–933	schizophrenia	role in neurogenesis and control regulatory pathways relevant to schizophrenia and psychotic illness
**PRICKLE1** (prickle-like protein 1)	Q96MT3	1–831	progressive myoclonic epilepsy	involved in planar cell polarity pathway that controls convergent extension during gastrulation and neural tube closure
**hnRNPA1** (Heterogeneous nuclear ribonucleoprotein A1)	P09651	1–372	Amyotrophic lateral sclerosis, frontotemporal dementia, myopathy	involved in packaging of pre-mRNA into hnRNP particles, transport of poly(A) mRNA from nucleus to cytoplasm, modulation of splice site selection, splicing of pyruvate kinase

**Table 2:** List of the computational tools used for prediction of condensates, aggregates, amyloids and prions.

	Predictor
*Prediction of condensate propensity*	PSPer [63], PSPredictor [64], LLPSDB [65], PhaSePred [66], CIDER [67], DeepPhase [68]
*prone regions*	FuzDrop [69], DeePhase [37], Pscore [70], Espritz [71], catGranule [72]
*Prediction of aggregation propensity*	GAP [73]
*prone regions*	SODA [74], FuzDrop [69], ANuPP [75], Aggrescan [76], TANGO [77]
*Prediction of amyloid propensity*	TANGO [78], WALTZ-DB 2.0 [78], SALSA [79], PASTA 2.0 [80], AmyloGram [81], MetAmyl [82]
*prone regions*	AmyLoad [83], FoldAmyloid [84], ArchCandy [85], Budapest Amyloid Predictor (BAP) [86], FiSH Amyloid [87], AmylPred2 [88], WALTZ [89], Amyloidogenic Pattern [90], Average Packing Density [91], Beta-strand contiguity [92], Hexapeptide Conf. Energy [93], NetCSSP [94], Pafig [95], SecStr [96]
*Prediction of prion propensity*	PrionScan [97], PrionW [98], AMYCO [99], SGnn [100], pWALTZ [101], pRANK [102]
*prone regions*	PLAAC [103], ZipperDB [104]

**Table 3:** Consensus of predictions of the condensation-, aggregation-, amyloid- and prion-prone regions.

Protein	Condensation Regions *	Aggregation Regions *	Amyloid Regions **	Prion Regions *
stefin A	1–11, 53–59, 64–73	66–72	16–20, 41–57, 65–70, 81–85	no
stefin B	1–12	37–42, 43–52, 54–58, 66–72	47–58, 64–72, 81–85	no
cystatin C	25–40, 44–49	9–20, 82–90, 122–130	9–20, 56–60, 73–77, 83–90, 124–130	no
α-synuclein	50–65, 101–113, 114–128, 25–128	14–19, 36–42, 55–63, 73–80	36–42, 54–65, 73–80	no
SOD1	28–104	30–38, 101–107, 114–121, 146–152	4–8, 15–22, 30–35, 44–50, 101–107, 112–120, 146–152	no
malin	133–182	44–59, 86–95, 121–128, 137–134, 183–187, 198–217, 241–247, 266–279, 283–325, 336–348, 366–372, 380–395	24–28, 48–58, 68–73, 86–92, 111–118, 130–134, 139–146, 151–156, 178–186, 199–213, 227–232, 239–244, 249–253, 266–279, 290–295 *, 301–305, 310–316, 323–327, 331–338, 367–382, 387–392	no
laforin	93–105	119–124, 144–152, 156–161, 180–191, 226–230, 248–256, 257–268, 275–287, 288–298	82–88, 119–126, 145–150, 163–167, 175–179, 185–192, 218–222, 226–230, 249–254, 259–267, 278–285, 291–298	no
RIP3	160–187, 329–333, 400–450, 434–447, 476–487	46–54, 120–131, 202–217	1–6, 34–38, 47–52, 70–75, 120–131, 137–141, 189–194, 204–216, 228–232, 262–266, 297–301, 344–348, 432–436, 449–454, 461–466	no
CRMP1	510–567, 525–547	33–38, 45–51, 98–109, 113–121, 136–144, 165–172, 181–198, 235–265, 429–434, 444–450	31–37, 47–51, 62–66, 72–76, 101–107, 137–146, 152–157, 163–169,182–189, 193–198, 235–242, 248–254, 262–266, 288–293, 299–304, 318–322, 348–352, 362–368, 379–386, 390–395, 429–434, 444–450	no
TRIOBP1	20–30, 140–214, 187–200, 280–295, 350–363, 397–420	39–43, 91–95, 551–558	12–16, 39–43, 82–86, 91–95, 105–109, 171–175, 487–491, 510–514, 552–558, 574–579	no
FUS	1–274, 360–437, 370–423, 440–526	283–312, 370–375	286–290, 294–299, 308–313, 322–326, 364–370, 437–441	49–53, 81–88, 112–119
tau	1–25, 26–50, 51–119, 120–134, 135–146, 147–230, 231–300, 231–300, 310–317, 318–330, 331–349, 350–455, 456–525, 526–573, 574–589, 590–605, 606–611, 612–622, 700–709, 719–739	186–195, 265–278, 613–620	299–308, 543–548, 591–596, 623–628, 707–711	no
TDP-43	255–269, 270–310, 311–344, 345–414	54–59, 70–75, 104–111, 125–135, 212–222, 228–235, 244–255, 332–336	3–7, 27–35, 54–60, 70–76, 105–111, 129–135, 148–154, 171–175, 192–196, 218–222, 228–234, 248–257, 313–318, 335–339	324–329, 368–381
DISC1	1–30, 180–200, 230–250, 285–315, 420–440	54–59, 205–217, 403–411, 624–633, 751–757	62–65, 118–122, 144–148, 210–217, 266–271, 348–354, 388–394, 400–406, 448–453, 474–479, 508–513, 557–562, 599–604, 628–632, 685–691, 699–705, 823–829, 844–850	no
LIMP2	no	10–26, 440–457	3–8, 9–12, 13–28, 39–43, 60–67, 131–136, 138–152, 166–171, 185–192, 199–204, 213–217, 223–227, 237–242, 264–269, 274–282, 310–315, 323–328, 335–339, 366–370, 374–378, 383–390, 403–411, 430–457	no
PRICKLE1	317–334, 367–376, 468–474, 481–493, 588–596, 657–679, 750–785, 795–831	138–143, 155–160, 168–172	45–50, 71–75, 97–102, 120–124, 138–143, 149–164, 165–172, 196–200, 214–219, 238–248, 289–293, 298–302, 417–421, 561–565, 616–620, 722–726, 750–754, 791–795	no
FABP3	no	83–87 *, 114–119 *	67–71, 82–86,93–97, 105–109, 114–119, 119–124	no
dysbindin 1	269–291, 323–327, 335–351	no	7–11, 80–85, 105–109, 214–218, 252–257	no
hnRNPA1	177–201, 202–370	57–65, 147–153	14–19, 42–46, 57–65, 104–112, 132–136, 148–152, 161–166	367–371
NPAS3	1–22, 472–515, 516–549, 563–583, 584–628, 629–674, 675–701, 702–726, 742–755, 763–790	707–719 *	33–37, 69–73, 92–101, 127–132, 141–145, 152–156, 160–165, 170–183, 193–197, 262–267,284–290, 294–299, 309–316, 328–337, 340–347, 359–364, 366–371, 391–399, 405–409, 410–417, 426–433, 644–648, 783–787, 801–808, 851–855, 870–876, 913–917	no

* consensus of ≥2 predictions, ** consensus of ≥4 predictions.

## Data Availability

Protein sequences used in this research are available in Supplementary Materials.

## Supplementary Materials

The following supporting information can be downloaded at https://www.mdpi.com/article/10.3390/biom16091286/s1, Figure S1. Stefin-A: (a) Predictions of condensation, aggregation, amyloid and prion prone regions (b) visualization of regions in 3D protein structure; Figure S2. Stefin-B: (a) Predictions of condensation, aggregation, amyloid and prion prone regions (b) visualization of regions in 3D protein structure; Figure S3. Cystatin-C: (a) Predictions of condensation, aggregation, amyloid and prion prone regions (b) visualization of regions in 3D protein structure; Figure S4. *α*-synuclein: (a) Predictions of condensation, aggregation, amyloid and prion prone regions (b) visualization of regions in 3D protein structure; Figure S5. SOD1: (a) Predictions of condensation, aggregation, amyloid and prion prone regions (b) visualization of regions in 3D protein structure; Figure S6. Malin: (a) Predictions of condensation, aggregation, amyloid and prion prone regions (b) visualization of regions in 3D protein structure; Figure S7. Laforin: (a) Predictions of condensation, aggregation, amyloid and prion prone regions (b) visualization of regions in 3D protein structure; Figure S8. RIP3: (a) Predictions of condensation, aggregation, amyloid and prion prone regions (b) visualization of regions in 3D protein structure; Figure S9. CRMP1: (a) Predictions of condensation, aggregation, amyloid and prion prone regions (b) visualization of regions in 3D protein structure; Figure S10. TRIOBP-1: (a) Predictions of condensation, aggregation, amyloid and prion prone regions (b) visualization of regions in 3D protein structure; Figure S11. FUS: (a) Predictions of condensation, aggregation, amyloid and prion prone regions (b) visualization of regions in 3D protein structure; Figure S12. TAU: (a) Predictions of condensation, aggregation, amyloid and prion prone regions (b) visualization of regions in 3D protein structure; Figure S13. TDP-43: (a) Predictions of condensation, aggregation, amyloid and prion prone regions (b) visualization of regions in 3D protein structure; Figure S14. DISC1: (a) Predictions of condensation, aggregation, amyloid and prion prone regions (b) visualization of regions in 3D protein structure; Figure S15. FABP3: (a) Predictions of condensation, aggregation, amyloid and prion prone regions (b) visualization of regions in 3D protein structure; Figure S16. LIMP2: (a) Predictions of condensation, aggregation, amyloid and prion prone regions (b) visualization of regions in 3D protein structure; Figure S17. DTNBP1: (a) Predictions of condensation, aggregation, amyloid and prion prone regions (b) visualization of regions in 3D protein structure; Figure S18. NPAS3: (a) Predictions of condensation, aggregation, amyloid and prion prone regions (b) visualization of regions in 3D protein structure; Figure S19. PRICKLE1: (a) Predictions of condensation, aggregation, amyloid and prion prone regions (b) visualization of regions in 3D protein structure; Figure S20. hnRNPA1: (a) Predictions of condensation, aggregation, amyloid and prion prone regions (b) visualization of regions in 3D protein structure; Table S1. Consensus of predictions of the condensation-, aggregation-, amyloid- and prion-prone regions according to the number of the same region predictions by different predictors; Table S2. List of computational tools for prediction of condensates and aggregates/amyloids/prions; Table S3. Results of predictions with computational tools for prediction of condensates and aggregates/amyloids/prions.
