# Supplementary material for: Assessment of Aggregation- and Condensation-Prone Regions of Proteins Involved in Neurodegenerative, Neurological and Mental-State Diseases
Source: Biomolecules. 2026 Sep 5;16(9):1286. doi: 10.3390/biom16091286 (PMC13604358; doi:10.3390/biom16091286)
Supplement: Supplementary file 1 [file biomolecules-16-01286-s001.zip › biomolecules_4439821_SI.pdf]

Assessment of aggregation and condensation prone regions of proteins involved in different brain diseases

Katja Venko and Eva Žerovnik

Legend:
- condensate prone region ≥ 3
- condensate prone region = 2
- amyloid prone region ≥3
- amyloid prone region ≥ 6
- aggregate and amyloid prone region
- aggregate prone region = 2
- aggregate prone region ≥ 3
- condensation prone region
- aggregation prone region
- amyloid prone region
- prione prone region

Figure S1. Stefin-A: (a) Predictions of condensation, aggregation, amyloid and prion prone regions (b) visualization of regions in 3D protein structure.

(a)

>stefin-A, P01040

MIPGGLSEAKPATPEIQEIVDKVKPQLEEKTNETYGKLEAVQYKTQVVAGTNYYIKVRAGDNKYMHLKVFKSLPGQNEDLVLTGYQVDKNKDDELTGF

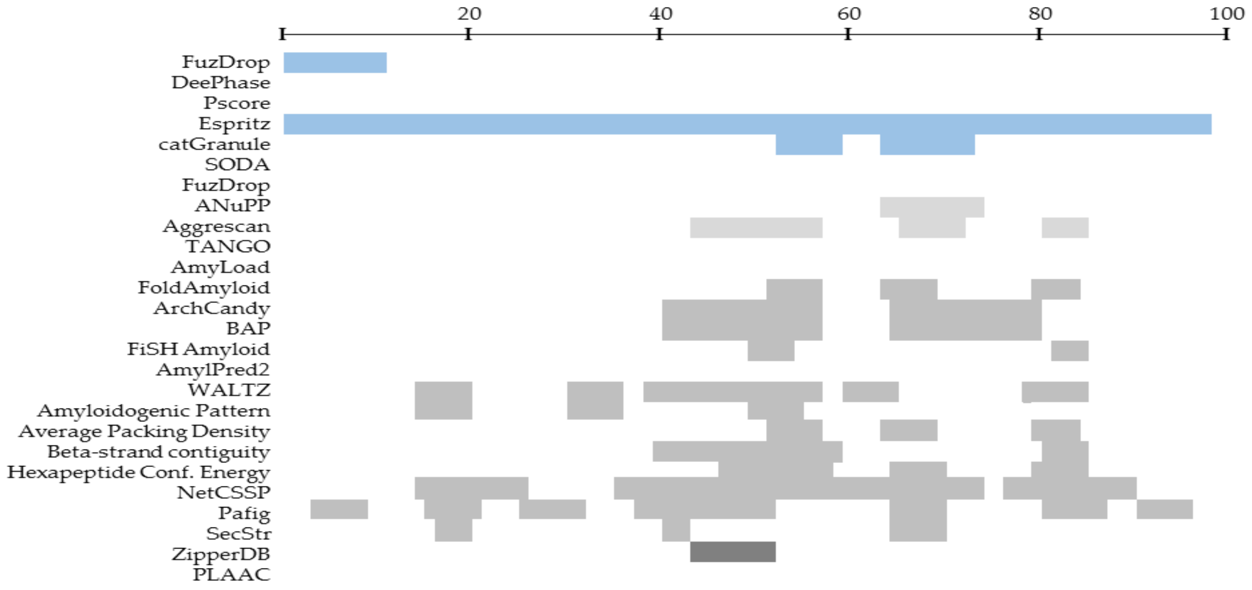

(b) PDB: **3KSE**

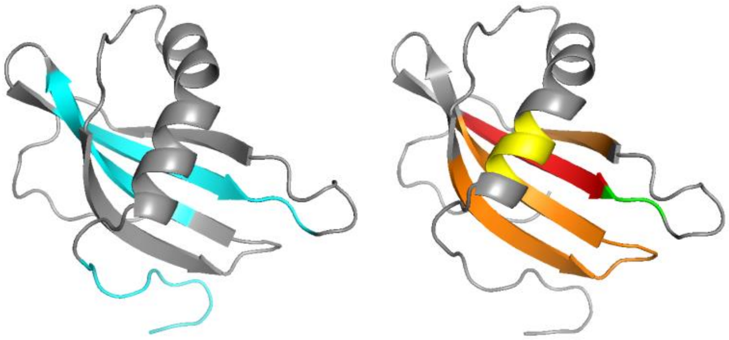

Condensation prone regions

1 10 20 30 40 50 60 70 80 90

MIPGGLSEAKPATPEIQEIVDKVKPQLEEKTNETYGKLEAVQYKTQVVAGTNYYIKVRAGDNKYMHLKVFKSLPGQNEDLVLTGYQVDKNKDDELTGF

Aggregation/amyloid prone regions

1 10 20 30 40 50 60 70 80 90

MIPGGLSEAKPATPEIQEIVDKVKPQLEEKTNETYGKLEAVQYKTQVVAGTNYYIKVRAGDNKYMHLKVFKSLP GQNEDLVLTGYQVDKNKDDELTGF

Figure S2. Stefin-B: (a) Predictions of condensation, aggregation, amyloid and prion prone regions (b) visualization of regions in 3D protein structure.

(a)

>stefin-B, P04080

MMCGAPSATQPATAETQHIADQVRSQLEEKENKKFPVFKAVSFKSQVVAGTNYFIKVHVGDEDFVHLRVFQSLPHENKPLTLSNYQTNKAKHDELTYF

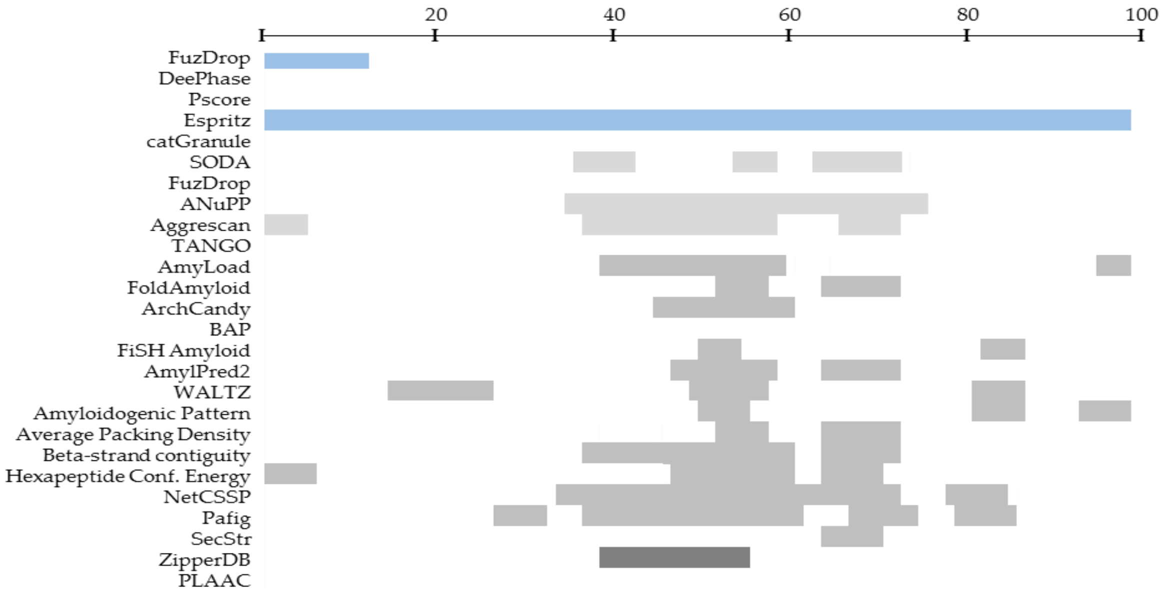

(b) PDB: **2OCT**

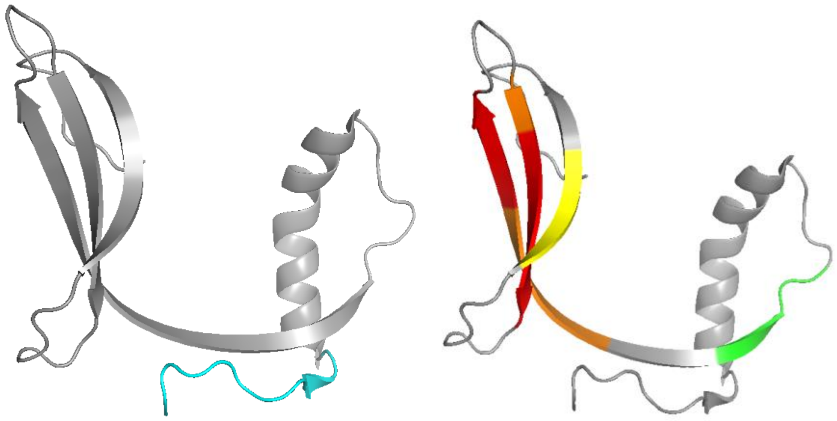

Condensation prone regions

1 10 20 30 40 50 60 70 80 90

MMCGAPSATQPATAETQHIADQVRSQLEEKENKKFPVFKAVSFKSQVVAGTNYFIKVHVGDEDFVHLRVFQSLPHENKPLTLSNYQTNKAKHDELTYF

Aggregation/amyloid prone regions

1 10 20 30 40 50 60 70 80 90

MMCGAPSATQPATAETQHIADQVRSQLEEKENKKFPVFKAVSFKSQVVAGTNYFIKVHVGDEDFVHLRVFQSLPHENKPLTLSNYQTNKAKHDELTYF

Figure S3. Cystatin-C: (a) Predictions of condensation, aggregation, amyloid and prion prone regions (b) visualization of regions in 3D protein structure.

(a)

>cystatin-C, P01034

MAGPLRAPLLLLAILAVALAVSPAAGSSPGKPPRLVGGPMDASVEEEGVRRALDFAVGEYNKASNDMYHSRALQVVRARKQIVAGVNYFLDVELGRTTC
TKTQPNLDNCPFHDQPHLKRKAFCSFQIYAVPWQGTMTLSKSTCQDA

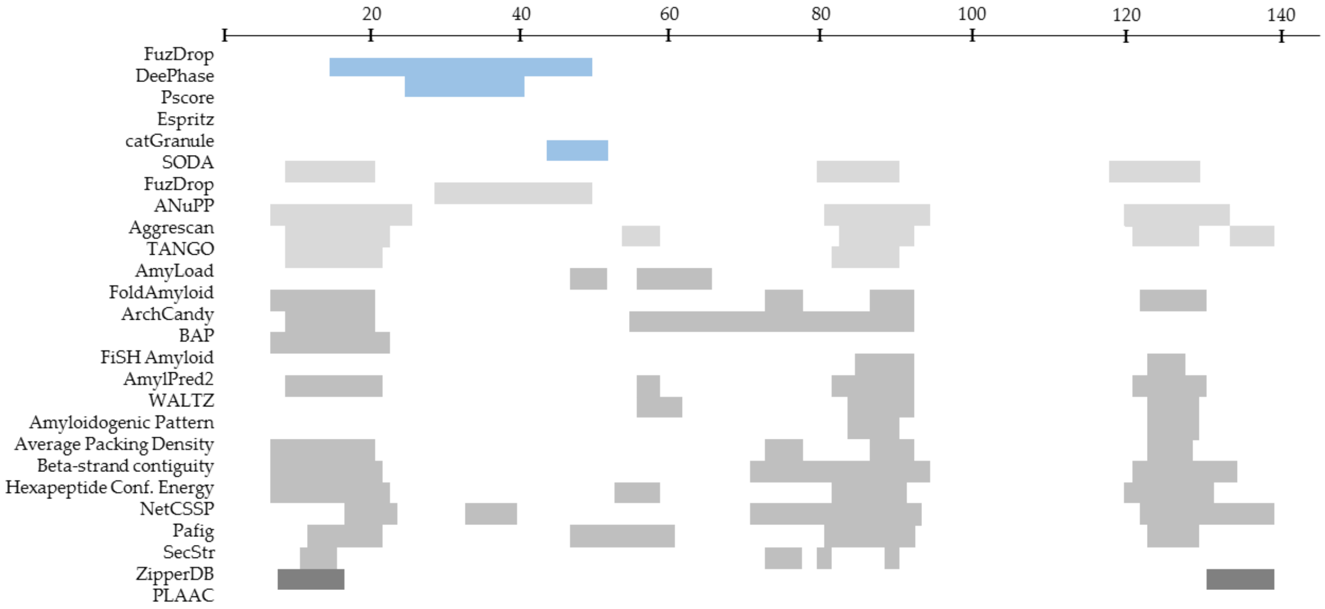

(b) AF: **P01034**

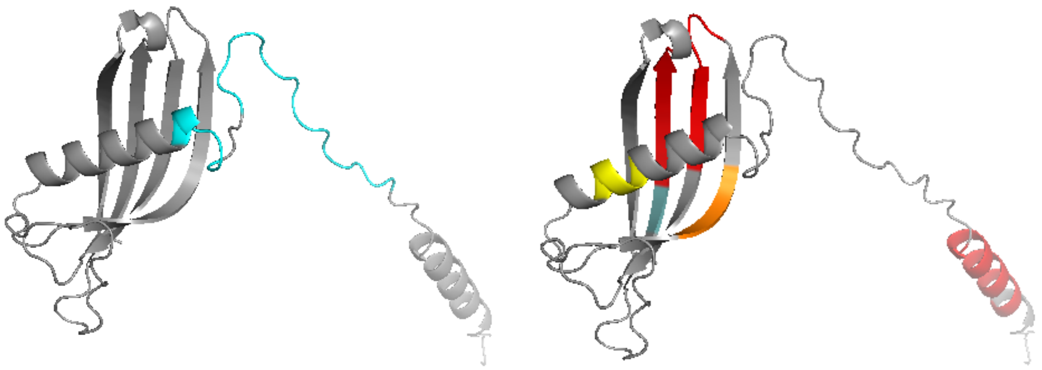

Condensation prone regions

1 10 20 30 40 50 60 70 80 90 100

MAGPLRAPLLLLAILAVALAVSPAAGSSPGKPPRLVGGPMDASVEEEGVRRALDFAVGEYNKASNDMYHSRALQVVRARKQIVAGVNYFLDVELGRTTCT

110 120 130 140

KTQPNLDNCPFHDQPHLKRKAFCSFQIYAVPWQGTMTLSKSTCQDA

Aggregation/amyloid prone regions

1 10 20 30 40 50 60 70 80 90 100

MAGPLRAPLLLLAILAVALAVSPAAGSSPGKPPRLVGGPMDASVEEEGVRRALDFAVGEYNKASNDMYHSRALQVVRARKQIVAGVNYFLDVELGRTTCT

110 120 130 140

KTQPNLDNCPFHDQPHLKRKAFCSFQIYAVPWQGTMTLSKSTCQDA

Figure S4. α-synuclein: (a) Predictions of condensation, aggregation, amyloid and prion prone regions (b) visualization of regions in 3D protein structure.

(a)

>α-synuclein, P37840

MDVFMKGLSKAKEGVVAAAEKTKQGVAEAAGKTKEGVLYVVAEKTKEQVTNVGGAVVTGVTAVAQKTVEGAGSIAAATGFVKKDQLGKNEEGAPQE GILEDMPVDPDNEAYEMPSEEGYQDYEPEA

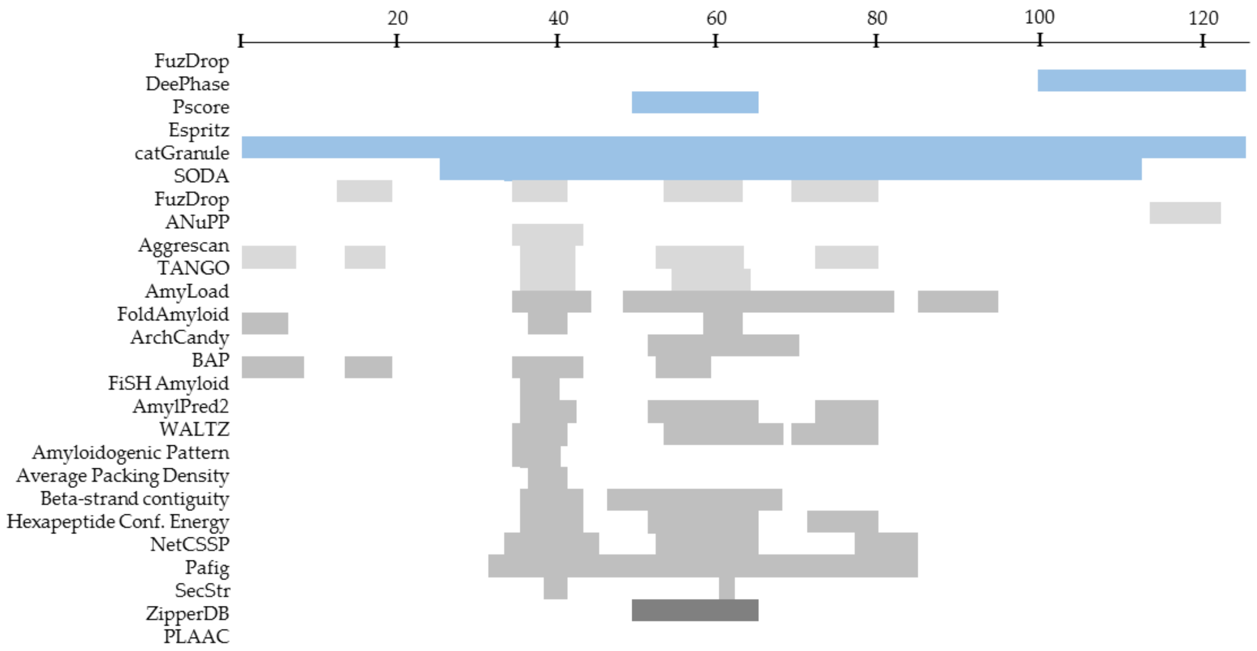

(b) PDB: **2NOA**

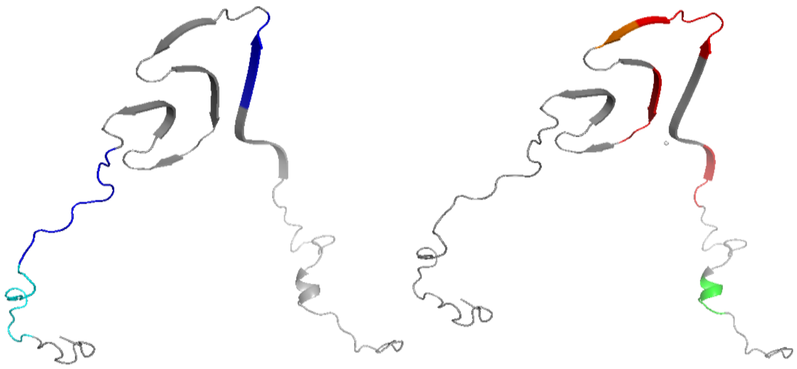

>synuclein, P37840

Condensation prone regions

1 10 20 30 40 50 60 70 80 90 100

MDVFMKGLSKAKEGVVAAAEKTKQGVAEAAGKTKEGVLYVGSKTKEGVVHGVATVAEKTKEQVTNVGGAVVTGVTAVAQKTVEGAGSIAAATGFVKKDQL

110 120 130 140

GKNEEGAPQEGILEDMPVDPDNEAYEMPSEEGYQDYEPEA

Aggregation/amyloid prone regions

1 10 20 30 40 50 60 70 80 90 100

MDVFMKGLSKAKEGVVAAAEKTKQGVAEAAGKTKEGVLYVGSKTKEGVVHGVATVAEKTKEQVTNVGGAVVTGVTAVAQKTVEGAGSIAAATGFVKKDQL

110 120 130 140

GKNEEGAPQEGILEDMPVDPDNEAYEMPSEEGYQDYEPEA

Figure S5. SOD1: (a) Predictions of condensation, aggregation, amyloid and prion prone regions (b) visualization of regions in 3D protein structure.

(a)

>SOD1,P00441

MATKAVCVLKGDGPVQGIINFEQKESNGPVKVWGSIKGLTEGLHGFHVHEFGDNTAGCTSAGPHFNPLSRKHGGPKDEERHVGDLGNVTADKDGVAD
VSIEDSVISLSGDHCIIGRTLVVHEKADDLGKGGNEESTKTGNAGSRLACGVIGIAQ

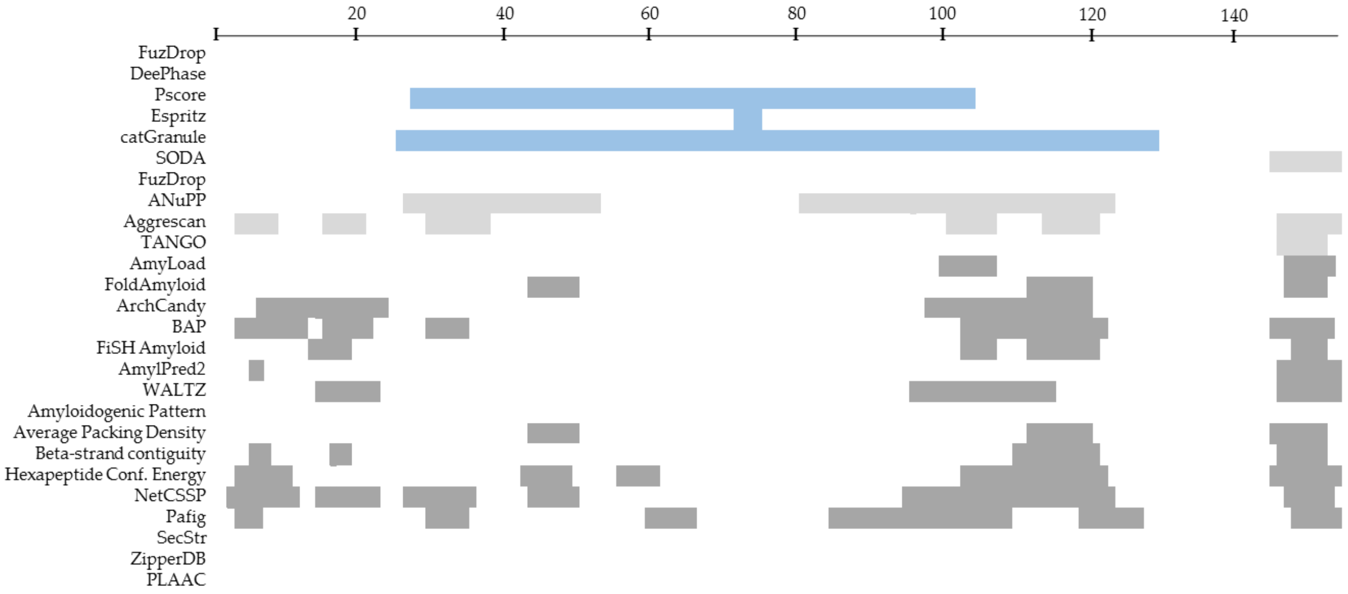

(b) PDB: **1N19**

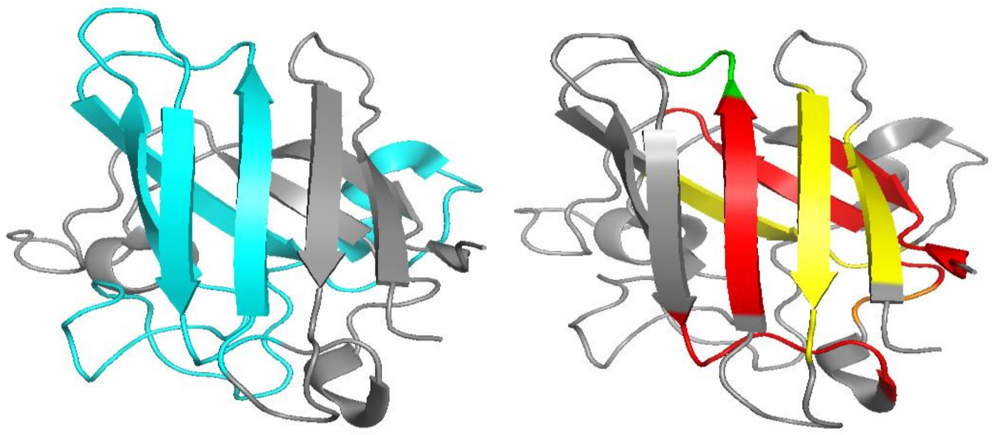

Condensation prone regions

1 10 20 30 40 50 60 70 80 90 100

MATKAVCVLKGDGPVQGIINFEQKESNGPVKVWGSIKGLTEGLHGFHVHEFGDNTAGCTSAGPHFNPLSRKHGGPKDEERHVGDLGNVTADKDGVADVSIE

110 120 130 140 150

DSVISLSGDHCIIGRTLVVHEKADDLGKGGNEESTKTGNAGSRLACGVIGIAQ

Aggregation/amyloid prone regions

1 10 20 30 40 50 60 70 80 90 100

MATKAVCVLKGDGPVQGIINFEQKESNGPVKVWGSIKGLTEGLHGFHVHEFGDNTAGCTSAGPHFNPLSRKHGGPKDEERHVGDLGNVTADKDGVADVSIE

110 120 130 140 150

DSVISLSGDHCIIGRTLVVHEKADDLGKGGNEESTKTGNAGSRLACGVIGIAQ

Figure S6. Malin: (a) Predictions of condensation, aggregation, amyloid and prion prone regions (b) visualization of regions in 3D protein structure.

(a)

>malin, Q6VVB1

MAAEASESGPALHELMREAEISLLECKVCFEKFGHRQQRRPRNLSCGHVVCLACVAALAHPRTLALECPFCRRACRGCDTSDCLPVLHLIELLGSALRQSP
AAHRAAPSAPGALTCHHTFGGWGTLVNPTGLALCPKTGRVVVVHDGRRRVKIFDSGGGCAHQFGEKGDAAQDIRYPVDVTITNDCHVVVTDAGDRSI
KVFDFFGQIKLVIGGQFSLPWGVETTPQNGIVVTDAEAGSLHLLDVDFAEGVLRRTERLQAHLCNPRGVAVSWLTGAIAVLEHPLALGTGVCSTRVKVFS
SSMQLVGQVDTFGLSLYFPSKITASAVTFDHQGNVIVADTSGPAILCLGKPEEFPVPKPMVTHGLSHPVALTFTKENSLLVLDTASHSIKVYKVDWG

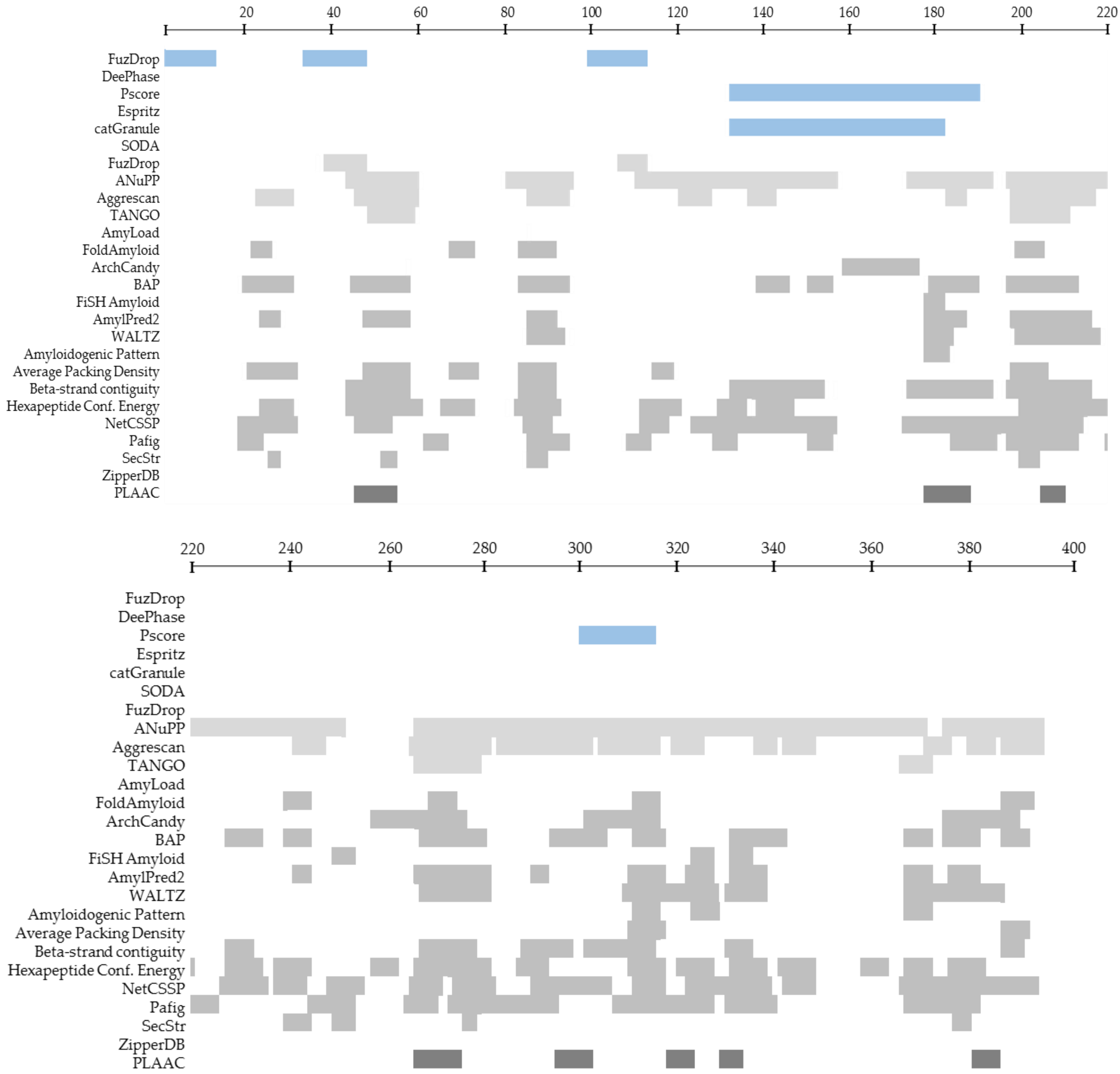

(b) AF: **Q6VVB1**

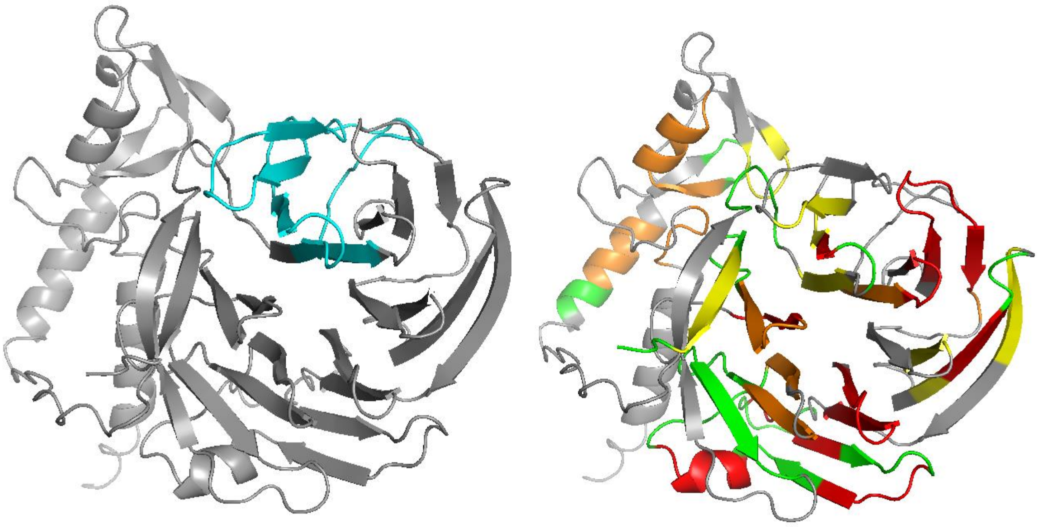

Condensation prone regions

1 10 20 30 40 50 60 70 80 90 100
MAAEASESGPALHELMREAEISLLECKVCFEKFGHRQQRRPRNLSCGHVVCLACVAALAHPRTLALECPFCRRACRGCDTSDCLPVLHLIELLGSALRQS
. 110 120 130 140 150 160 170 180 190 200
PAAHRAAPSAPGALTCHHTFGGWGTLVNPTGLALCPKTGRVVVVHDGRRRVKIFDSGGGCAHQFGEKGDAAQDIRYPVDVTITNDCHVVVTDAGDRSIKV
. 210 220 230 240 250 260 270 280 290 300
FDFFGQIKLVIGGQFSLPWGVETTPQNGIVVTDAEAGSLHLLDVDFAEGVLRRTERLQAHLCNPRGVAVSWLTGAIAVLEHPLALGTGVCSTRVKVFSSS
. 310 320 330 340 350 360 370 380 390
MQLVGQVDTFGLSLYFPSKITASAVTFDHQGNVIVADTSGPAILCLGKPEEFPVPKPMVTHGLSHPVALTFTKENSLLVLDTASHSIKVYKVDWG

Aggregation/amyloid prone regions

1 10 20 30 40 50 60 70 80 90 100
MAAEASESGPALHELMREAEISLLECKVCFEKFGHRQQRRPRNLSCGHVVCLACVAALAHPRTLALECPFCRRACRGCDTSDCLPVLHLIELLGSALRQS
. 110 120 130 140 150 160 170 180 190 200
PAAHRAAPSAPGALTCHHTFGGWGTLVNPTGLALCPKTGRVVVVHDGRRRVKIFDSGGGCAHQFGEKGDAAQDIRYPVDVTITNDCHVVVTDAGDRSIKV
. 210 220 230 240 250 260 270 280 290 300
FDFFGQIKLVIGGQFSLPWGVETTPQNGIVVTDAEAGSLHLLDVDFAEGVLRRTERLQAHLCNPRGVAVSWLTGAIAVLEHPLALGTGVCSTRVKVFSSS
. 310 320 330 340 350 360 370 380 390
MQLVGQVDTFGLSLYFPSKITASAVTFDHQGNVIVADTSGPAILCLGKPEEFPVPKPMVTHGLSHPVALTFTKENSLLVLDTASHSIKVYKVDWG

Figure S7. Laforin: (a) Predictions of condensation, aggregation, amyloid and prion prone regions (b) visualization of regions in 3D protein structure.

(a)

>laforin,O95278

MRFRFGVVVPPAVAGARPELLVVGSRPELGRWEPRGAVRLRPAGTAAGDGALALQEPGLWLGEVELAAEEAAQDGAEPGRVDTFWYKFLKREPGGEL
SWEGNGPHHDRCCTYNENNLVDGVYCLPIGHWIEATGHTNEMKHTTDFYFNIAGHQAMHYSRILPNIWLGSCPRQVEHVTIKLKHELGITAVMNFQTE
WDIVQNSSGCNRYPEPMTPDTMIKLYREEGLAYIWMPTPDMSTEGRVQMLPQAVCLLHALLEKGHIVYVHCNAGVGRSTAAVCGWLQYVMGWNLR
KVQYFLMAKRPAVYIDEEALARAQEDFFQKFGKVRSSVCSL

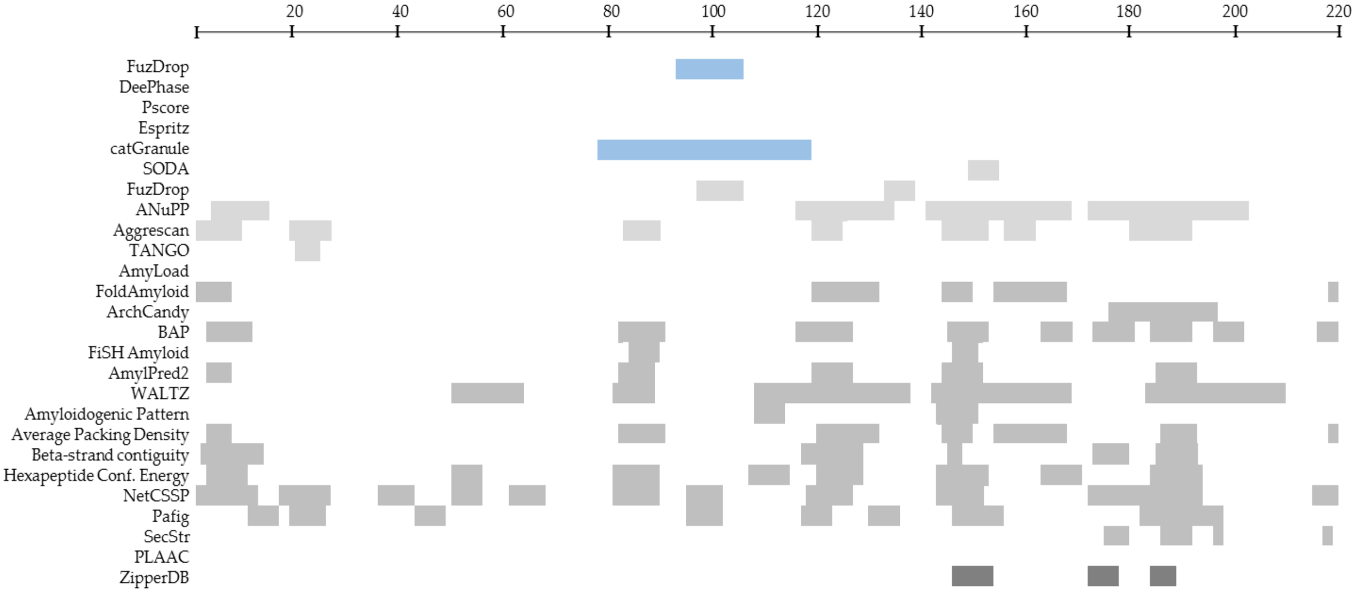

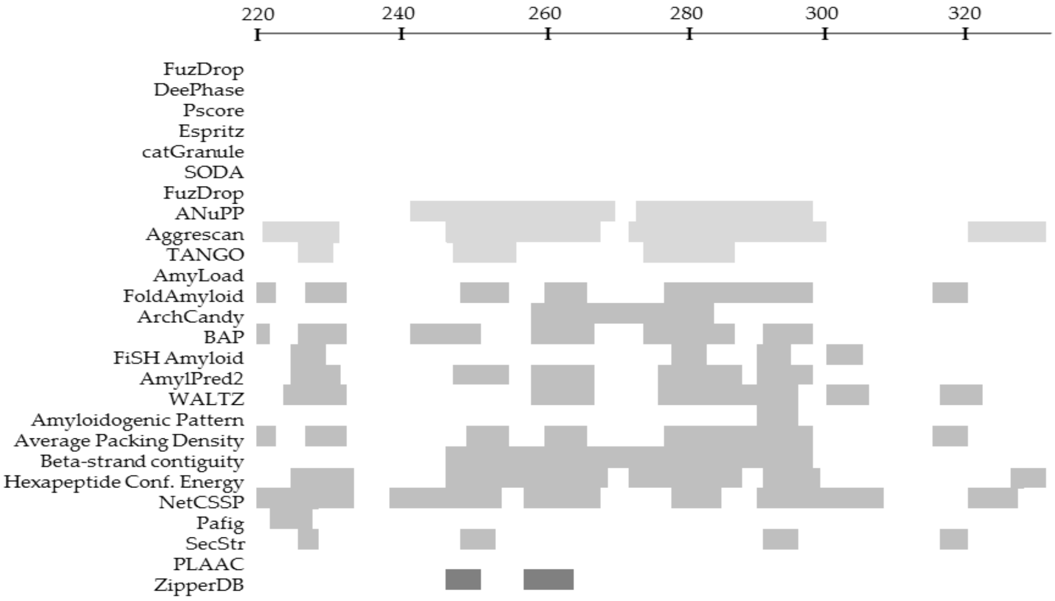

(b) PDB: **4RKK**

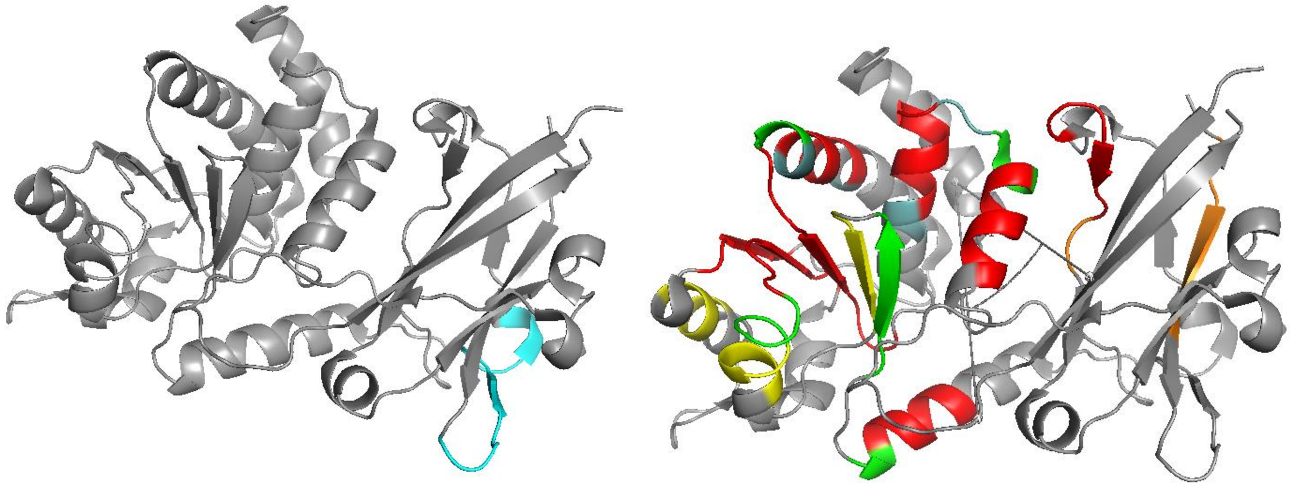

Condensation prone regions

1 10 20 30 40 50 60 70 80 90 100
MRFRFGVVVPPAVAGARPELLVVGSRPELGRWEPRGAVRLRPAGTAAGDGALALQEPGLWLGEVELAAEEAAQDGAEPGRVDTFWYKFLKREPGGELSWE
. 110 120 130 140 150 160 170 180 190 200
GNGPHHDRCCTYNENNLVDGVYCLPIGHWIEATGHTNEMKHTTDFYFNIAGHQAMHYSRILPNIWLGSCPRQVEHVTIKLKHELGITAVMNFQTEWDIVQ
. 210 220 230 240 250 260 270 280 290 300
NSSGCNRYPEPMTPDTMIKLYREEGLAYIWMPTPDMSTEGRVQMLPQAVCLLHALLEKGHIVYVHCNAGVGRSTAAVCGWLQYVMGWNLRKVQYFLMAKR
. 310 320 330
PAVYIDEEALARAQEDFFQKFGKVRSSVCSL

Aggregation/amyloid prone regions

1 10 20 30 40 50 60 70 80 90 100
MRFRFGVVVPPAVAGARPELLVVGSRPELGRWEPRGAVRLRPAGTAAGDGALALQEPGLWLGEVELAAEEAAQDGAEPGRVDTFWYKFLKREPGGELSWE
. 110 120 130 140 150 160 170 180 190 200
GNGPHHDRCCTYNENNLVDGVYCLPIGHWIEATGHTNEMKHTTDFYFNIAGHQAMHYSRILPNIWLGSCPRQVEHVTIKLKHELGITAVMNFQTEWDIVQ
. 210 220 230 240 250 260 270 280 290 300
NSSGCNRYPEPMTPDTMIKLYREEGLAYIWMPTPDMSTEGRVQMLPQAVCLLHALLEKGHIVYVHCNAGVGRSTAAVCGWLQYVMGWNLRKVQYFLMAKR
. 310 320 330
PAVYIDEEALARAQEDFFQKFGKVRSSVCSL

Figure S8. RIP3: (a) Predictions of condensation, aggregation, amyloid and prion prone regions (b) visualization of regions in 3D protein structure.

(a)

>RIP3,Q9Y572

MSCVKLWPSGAPAPLVSIEELENQELVGKGGFGTVFRAQHRKWGYDVAVKIVNSKAISREVKAMASLDNEFVLRLEGVIEKVNWDQDPKPALVTKFME
NGSLSGLLQSQCPRPWPLLCRLLKEVVLGMFYLHDQNPVLLHRDLKPSNVLLDPELHVKLADFGLSTFQGGSQSGTGSGEPGGTLGYLAPELFVNVNRKA
STASDVYSFGILMWAVLAGREVELPTEPSLVYEAVCNRQNRPSLAELPQAGPETPGLEGLKELMQLCWSSEPKDRPSFQECLPKTDEVFQMVENNMNA
AVSTVKDFLSQLRSSNRRFSIPESGQGGTEMDGFRRTIENQHSRNDVMVSEWLNKLNLEEPPSSVPKKCPSLTKRSRAQEEQVPQAWTAGTSSDSMAQ
PPQTPETSTFRNQMPSPTSTGTPSPGPRGNQGAERQGMNWSCRTPEPNPVTGRPLVNIYNCSGVQVGDNNYLTMQQTTALPTWGLAPSGKGRGLQ
HPPPVGSQEGPKDPEAWSRPQGWYNHSGK

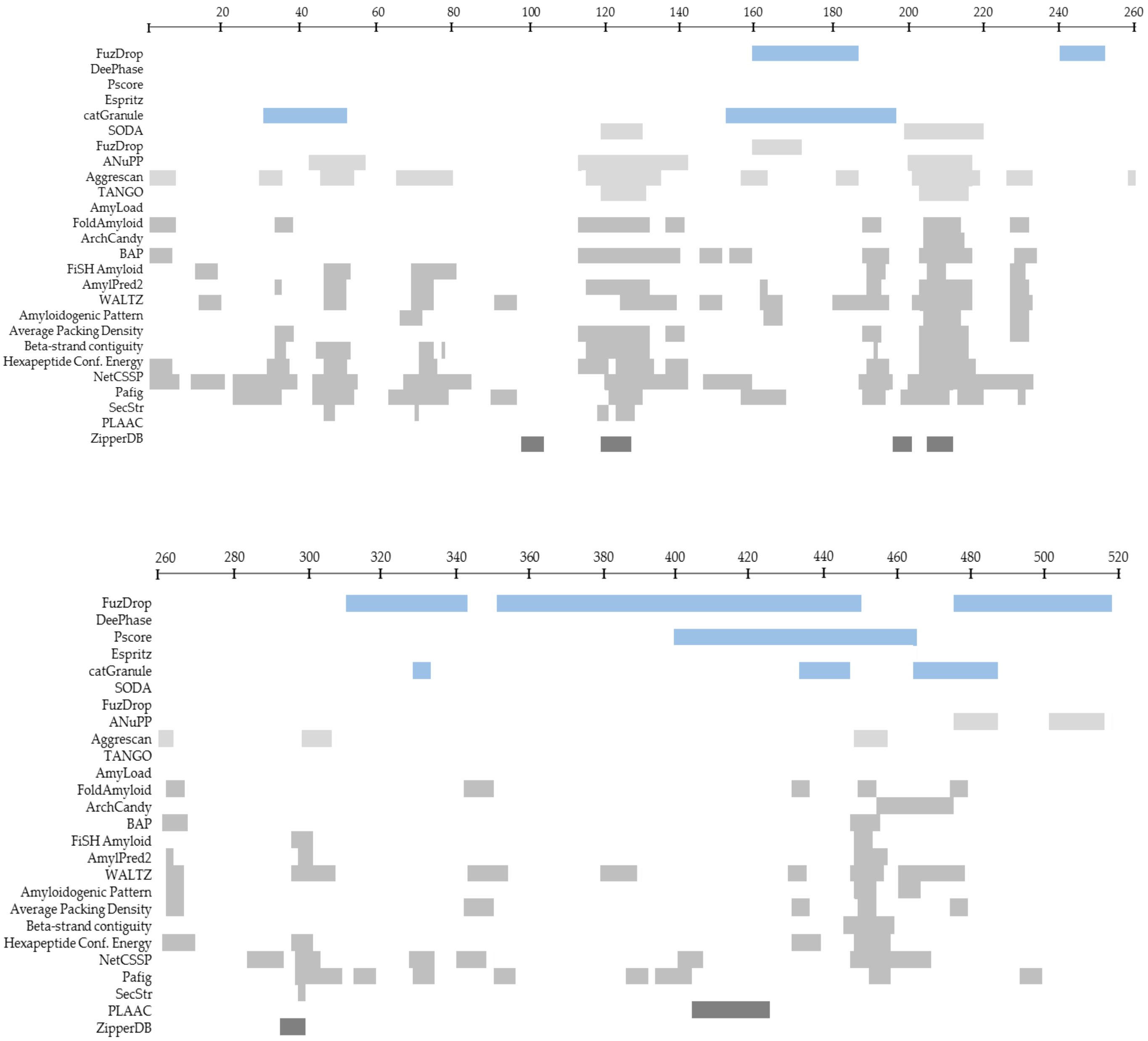

(b) AF: **Q9Y572**

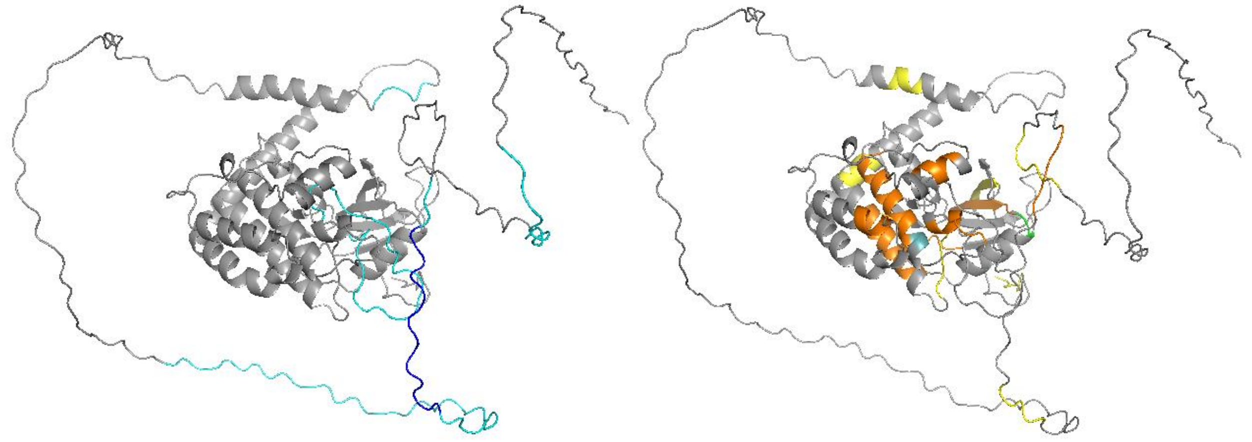

Condensation prone regions

1 10 20 30 40 50 60 70 80 90 100

MSCVKLWPSGAPAPLVSIEELENQELVGKGGFGTVFRAQHRKWGYDVAVKIVNSKAISREVKAMASLDNEFVLRLEGVIEKVNWDQDPKPALVTKFMENG

. 110 120 130 140 150 160 170 180 190 200

SLSGLLQSQCPRPWPLLCRLLKEVVLGMFYLHDQNPVLLHRDLKPSNVLLDPELHVKLADFGLSTFQGGSQSGTGSGEPGGTLGYLAPELFVNVNRKAST .

210 220 230 240 250 260 270 280 290 300

ASDVYSFGILMWAVLAGREVELPTEPSLVYEAVCNRQNRPSLAELPQAGPETPGLEGLKELMQLCWSSEPKDRPSFQECLPKTDEVFQMVENNMNAAVST

. 310 320 330 340 350 360 370 380 390 400

VKDFLSQLRSSNRRFSIPESGQGGTEMDGFRRTIENQHSRNDVMVSEWLNKLNLEEPPSSVPKKCPSLTKRSRAQEEQVPQAWTAGTSSDSMAQPPQTPET

. 410 420 430 440 450 460 470 480 490 500

STFRNQMPSPTSTGTPSPGPRGNQGAERQGMNWSCRTPEPNPVTGRPLVNIYNCSGVQVGDNNYLTMQQTTALPTWGLAPSGKGRGLQHPPPVGSQEGP

. 510

KDPEAWSRPQGWYNHSGK

Aggregation/amyloid prone regions

1 10 20 30 40 50 60 70 80 90 100

MSCVKLWPSGAPAPLVSIEELENQELVGKGGFGTVFRAQHRKWGYDVAVKIVNSKAISREVKAMASLDNEFVLRLEGVIEKVNWDQDPKPALVTKFMENG

. 110 120 130 140 150 160 170 180 190 200

SLSGLLQSQCPRPWPLLCRLLKEVVLGMFYLHDQNPVLLHRDLKPSNVLLDPELHVKLADFGLSTFQGGSQSGTGSGEPGGTLGYLAPELFVNVNRKAST .

210 220 230 240 250 260 270 280 290 300

ASDVYSFGILMWAVLAGREVELPTEPSLVYEAVCNRQNRPSLAELPQAGPETPGLEGLKELMQLCWSSEPKDRPSFQECLPKTDEVFQMVENNMNAAVST

. 310 320 330 340 350 360 370 380 390 400

VKDFLSQLRSSNRRFSIPESGQGGTEMDGFRRTIENQHSRNDVMVSEWLNKLNLEEPPSSVPKKCPSLTKRSRAQEEQVPQAWTAGTSSDSMAQPPQTPET

. 410 420 430 440 450 460 470 480 490 500

STFRNQMPSPTSTGTPSPGPRGNQGAERQGMNWSCRTPEPNPVTGRPLVNIYNCSGVQVGDNNYLTMQQTTALPTWGLAPSGKGRGLQHPPPVGSQEGP

. 510

KDPEAWSRPQGWYNHSGK

Figure S9. CRMP1: (a) Predictions of condensation, aggregation, amyloid and prion prone regions (b) visualization of regions in 3D protein structure.

(a)

>CRMP1,Q14194

MSYQGKKSIPHITSDRLLIKGGRIINDDQSLYADVYLEDGLIKQIGENLIVPGGVKTIEANGRMVIPGGIDVNTYLQKPSQGMTAADDFFQGTRAALVGGT
TMIIDHVVPEPGSSLLTSFEKWHEAADTKSCCDYSLHVDITSWYDGVREELEVLVQDKGVNSFQVYMAYKDVYQMSDSQLYEAFTFLKGLGAVILVHAE
NGDLIAQEQKRILEMGITGPEGHALSRPEELEAEAVFRAITIAGRINCPVYITKVMSKSAADIIALARKKGPLVFGEPIAASLGTDGTHYWSKNWAKAAAFV
TSPPLSPDPTTPDYLTSLLACGDLQVTGSGHCPYSTAQKAVGKDNFTLIPEGVNGIEERMTVVWDKAVATGKMDENQFVAVTSTNAAKIFNLYPRKGRIA
VGSDADVVIWDPDKLKTITAKSHKSAVEYNIFEGMECHGSPLVVISQGKIVFEDGNINVNKGMGRFIPRKAFPEHLYQRVKIRNKVFGLQGVSRGMYDG
PVYEVPATPKYATPAPSAKSSPSKHQPPPIRNLHQSNFSLSGAQIDDNNPRRTGHRIVAPPGGRSNITSLG

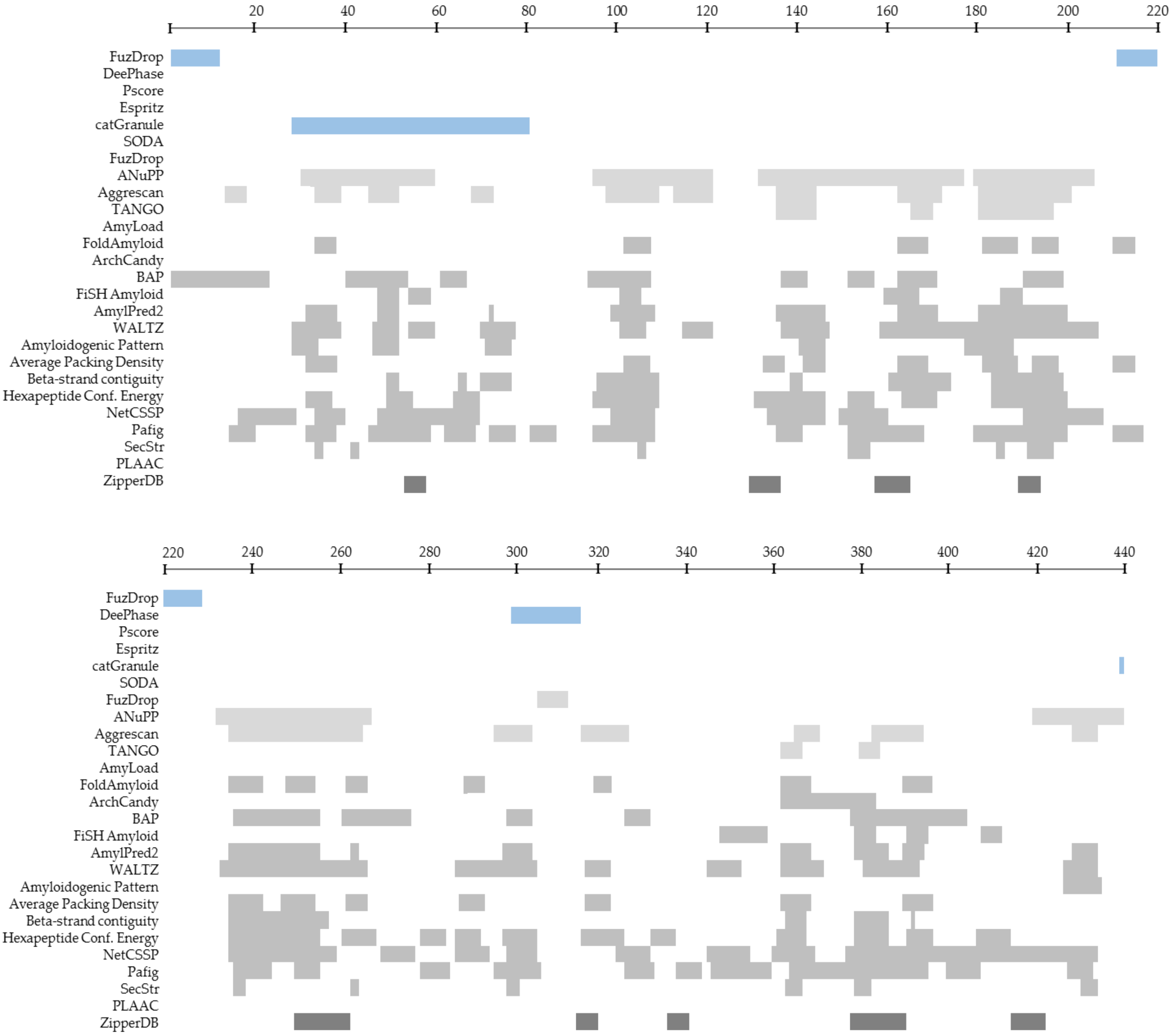

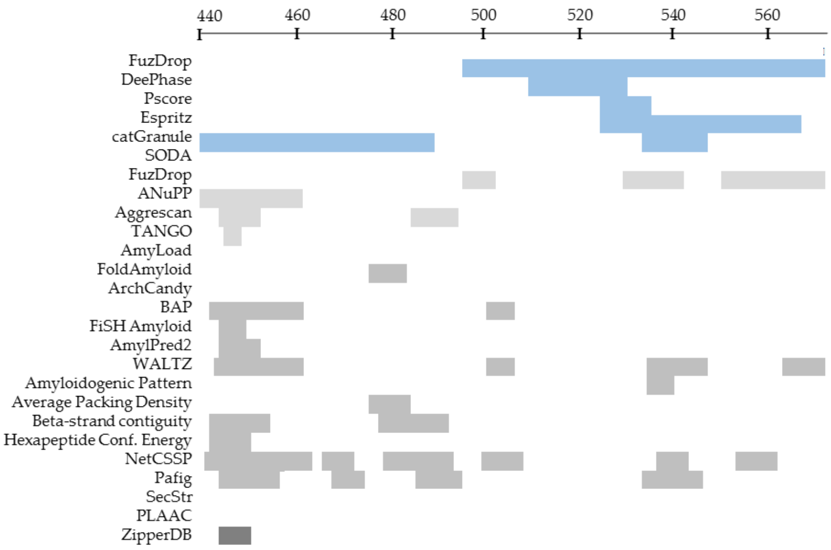

(b) AF: **Q14194**

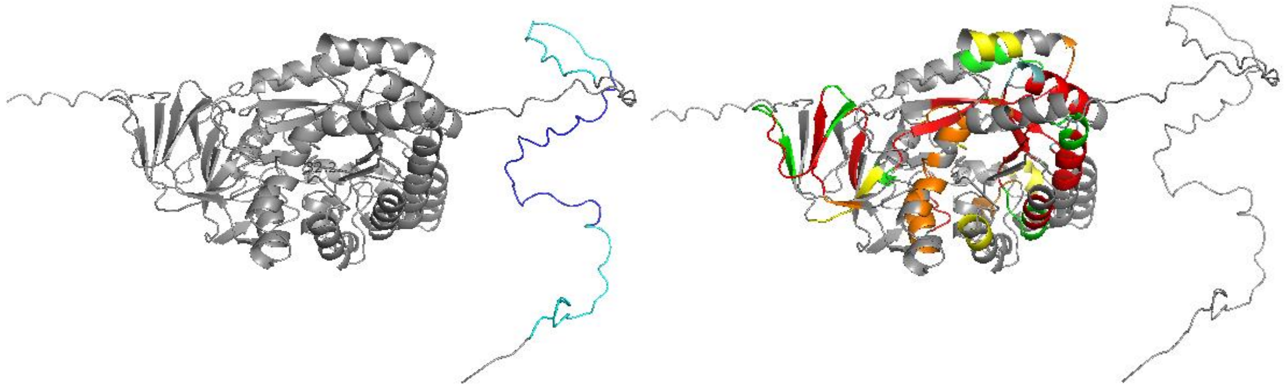

Condensation prone regions

1 10 20 30 40 50 60 70 80 90 100
MSYQGKKSIPHITSDRLLIKGGRIINDDQSLYADVYLEDGLIKQIGENLIVPGGVKTIEANGRMVIPGGIDVNTYLQKPSQGMTAADDFFQGTRAALVGG
. 110 120 130 140 150 160 170 180 190 200
TTMIIDHVVPEPGSSLLTSFEKWHEAADTKSCCDYSLHVDITSWYDGVREELEVLVQDKGVNSFQVYMAYKDVYQMSDSQLYEAFTFLKGLGAVILVHAE
. 210 220 230 240 250 260 270 280 290 300
NGDLIAQEQKRILEMGITGPEGHALSRPEELEAEAVFRAITIAGRINCPVYITKVMSKSAADIIALARKKGPLVFGEPIAASLGTDGTHYWSKNWAKAAA
. 310 320 330 340 350 360 370 380 390 400
FVTSPPLSPDPTTPDYLTSLLACGDLQVTGSGHCPYSTAQKAVGKDNFTLIPEGVNGIEERMTVVWDKAVATGKMDENQFVAVTSTNAAKIFNLYPRKGR
. 410 420 430 440 450 460 470 480 490 500
IAVGSDADVVIWDPDKLKTITAKSHKSAVEYNIFEGMECHGSPLVVISQGKIVFEDGNINVNKGMGRFIPRKAFPEHLYQRVKIRNKVFGLQGVSRGMYD
. 510 520 530 540 550 560 570
GPVYEVPATPKYATPAPSAKSSPSKHQPPPIRNLHQSNFSLSGAQIDDNNPRRTGHRIVAPPGGRSNITSLG

Aggregation/amyloid prone regions

1 10 20 30 40 50 60 70 80 90 100
MSYQGKKSIPHITSDRLLIKGGRIINDDQSLYADVYLEDGLIKQIGENLIVPGGVKTIEANGRMVIPGGIDVNTYLQKPSQGMTAADDFFQGTRAALVGG
. 110 120 130 140 150 160 170 180 190 200
TTMIIDHVVPEPGSSLLTSFEKWHEAADTKSCCDYSLHVDITSWYDGVREELEVLVQDKGVNSFQVYMAYKDVYQMSDSQLYEAFTFLKGLGAVILVHAE
. 210 220 230 240 250 260 270 280 290 300
NGDLIAQEQKRILEMGITGPEGHALSRPEELEAEAVFRAITIAGRINCPVYITKVMSKSAADIIALARKKGPLVFGEPIAASLGTDGTHYWSKNWAKAAA
. 310 320 330 340 350 360 370 380 390 400
FVTSPPLSPDPTTPDYLTSLLACGDLQVTGSGHCPYSTAQKAVGKDNFTLIPEGVNGIEERMTVVWDKAVATGKMDENQFVAVTSTNAAKIFNLYPRKGR
. 410 420 430 440 450 460 470 480 490 500
IAVGSDADVVIWDPDKLKTITAKSHKSAVEYNIFEGMECHGSPLVVISQGKIVFEDGNINVNKGMGRFIPRKAFPEHLYQRVKIRNKVFGLQGVSRGMYD
. 510 520 530 540 550 560 570
GPVYEVPATPKYATPAPSAKSSPSKHQPPPIRNLHQSNFSLSGAQIDDNNPRRTGHRIVAPPGGRSNITSLG

Figure S10. TRIOBP-1: (a) Predictions of condensation, aggregation, amyloid and prion prone regions (b) visualization of regions in 3D protein structure.

(a)

>TRIOBP-1, Q9H2D6

MTPDLLNFKKGWMSILDEPGEPPSPSLTTTSTSQWKKHWFVLTDSSLKYYRDSTAEEADELDGEIDLRSCTDVTEYAVQRNYGFQIHTKDAVYTLSAMTS
GIRRNWIEALRKTVRPTSAPDVTKLSDSNKENALHSYSTQKGPLKAGEQRAGSEVISRGGPRKADGQRQALDYVELSPLTQASPQRARTPARTPDRLAKQ
EELERDLAQRSEERRKWFEATDSRTPEVPAGEGPRRGLGAPLTEDQQNRLSEEIEKKWQELEKLPLRENKRVPLTALLNQSRGERRGPPSDGHEALEKEV
QALRAQLEAWRLQGEAPQSALRSQEDGHIPPGYISQEACERSLAEMESSHQQVMEELQRHHERELQRLQQEKEWLLAEETAATASAIEAMKKAYQEEL
SRELSKTRSLQQGPDGLRKQHQSDVEALKRELQVLSEQYSQKCLEIGALMRQAEEREHTLRRCQQEGQELLRHNQELHGRLSEEIDQLRGFIASQGMGN
GCGRSNERSSCELEVLLRVKENELQYLKKEVQCLRDELQMMQKDKRFTSGKYQDVYVELSHIKTRSEREIEQLKEHLRLAMAALQEKESMRNSLAE

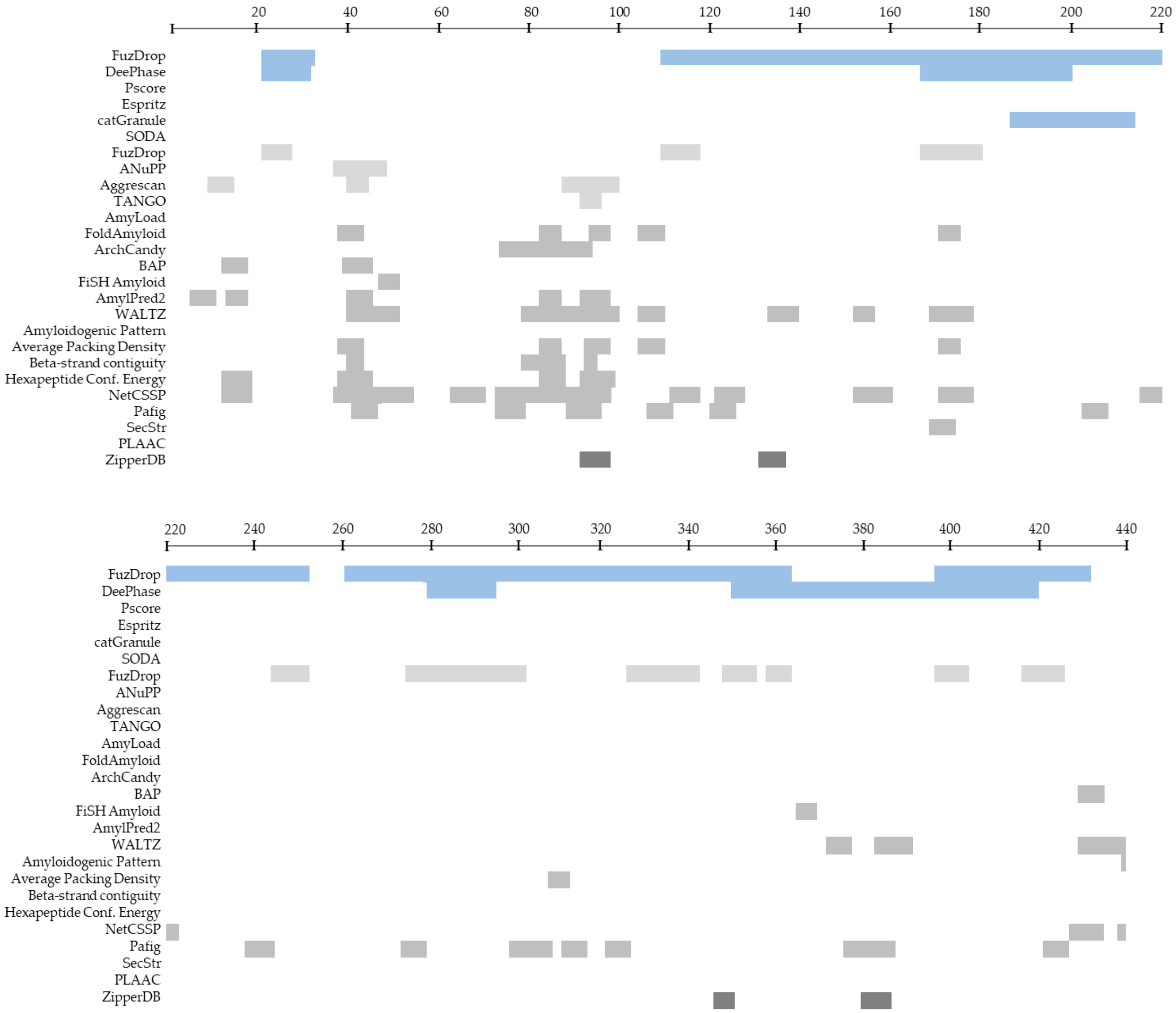

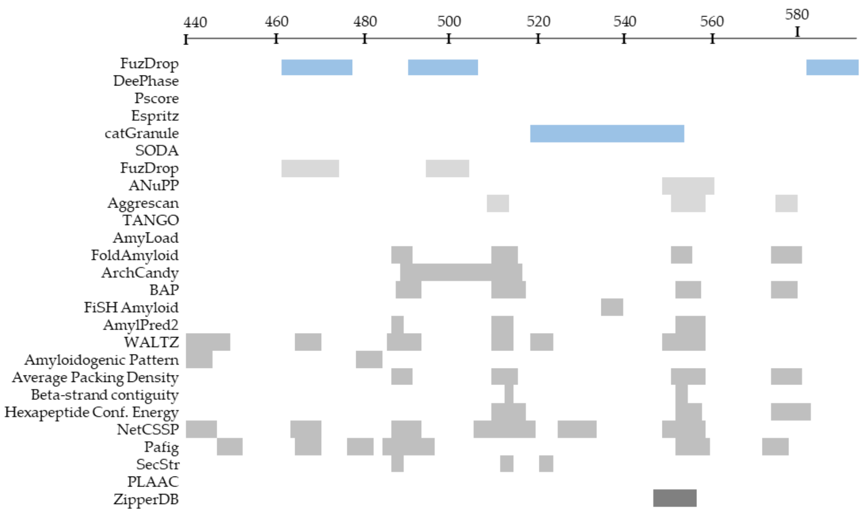

(b) AF: **Q9H2D6**

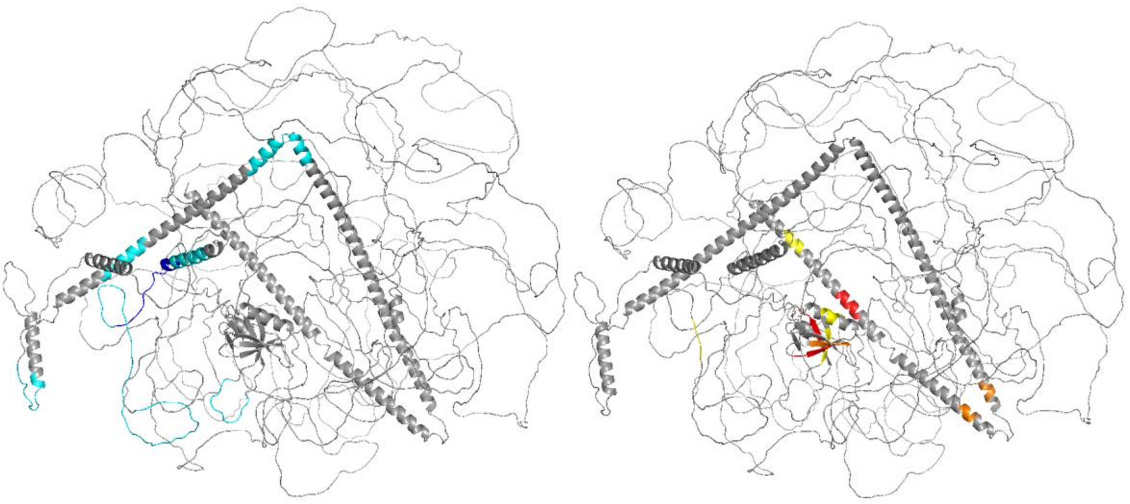

Condensation prone regions

1 10 20 30 40 50 60 70 80 90 100
MTPDLLNFKKGWMSILDEPGEPPSPSLTTTSTSQWKKHWFVLTDSSLKYYRDSTAEEADELDGEIDLRSCTDVTEYAVQRNYGFQIHTKDAVYTLSAMTS
. 110 120 130 140 150 160 170 180 190 200
GIRRNWIEALRKTVRPTSAPDVTKLSDSNKENALHSYSTQKGPLKAGEQRAGSEVISRGGPRKADGQRQALDYVELSPLTQASPQRARTPARTPDRLAKQ
. 210 220 230 240 250 260 270 280 290 300
EELERDLAQRSEERRKWFEATDSRTPEVPAGEGPRRGLGAPLTEDQQNRLSEEIEKKWQELEKLPLRENKRVPLTALLNQSRGERRGPPSDGHEALEKEV
. 310 320 330 340 350 360 370 380 390 400
QALRAQLEAWRLQGEAPQSALRSQEDGHIPPGYISQEACERSLAEMESSHQQVMEELQRHHERELQRLQQEKEWLLAEETAATASAIEAMKKAYQEELSR
. 410 420 430 440 450 460 470 480 490 500
ELSKTRSLQQGPDGLRKQHQSDVEALKRELQVLSEQYSQKCLEIGALMRQAEEREHTLRRCQQEGQELLRHNQELHGRLSEEIDQLRGFIASQGMGNGCG
. 510 520 530 540 550 560 570 580 590
RSNERSSCELEVLLRVKENELQYLKKEVQCLRDELQMMQKDKRFTSGKYQDVYVELSHIKTRSEREIEQLKEHLRLAMAALQEKESMRNSLAE

Aggregation/amyloid prone regions

1 10 20 30 40 50 60 70 80 90 100
MTPDLLNFKKGWMSILDEPGEPPSPSLTTTSTSQWKKHWFVLTDSSLKYYRDSTAEEADELDGEIDLRSCTDVTEYAVQRNYGFQIHTKDAVYTLSAMTS
. 110 120 130 140 150 160 170 180 190 200
GIRRNWIEALRKTVRPTSAPDVTKLSDSNKENALHSYSTQKGPLKAGEQRAGSEVISRGGPRKADGQRQALDYVELSPLTQASPQRARTPARTPDRLAKQ
. 210 220 230 240 250 260 270 280 290 300
EELERDLAQRSEERRKWFEATDSRTPEVPAGEGPRRGLGAPLTEDQQNRLSEEIEKKWQELEKLPLRENKRVPLTALLNQSRGERRGPPSDGHEALEKEV
. 310 320 330 340 350 360 370 380 390 400
QALRAQLEAWRLQGEAPQSALRSQEDGHIPPGYISQEACERSLAEMESSHQQVMEELQRHHERELQRLQQEKEWLLAEETAATASAIEAMKKAYQEELSR
. 410 420 430 440 450 460 470 480 490 500
ELSKTRSLQQGPDGLRKQHQSDVEALKRELQVLSEQYSQKCLEIGALMRQAEEREHTLRRCQQEGQELLRHNQELHGRLSEEIDQLRGFIASQGMGNGCG
. 510 520 530 540 550 560 570 580 590
RSNERSSCELEVLLRVKENELQYLKKEVQCLRDELQMMQKDKRFTSGKYQDVYVELSHIKTRSEREIEQLKEHLRLAMAALQEKESMRNSLAE

Figure S11. FUS: (a) Predictions of condensation, aggregation, amyloid and prion prone regions (b) visualization of regions in 3D protein structure.

(a)

>FUS,P35637

MASNDYTQQATQSYGAYPTQPGQGYSQQSSQPYGQQSYSGYSQSTDTSGYGQSSYSSYGQSQNTGYGTQSTPQGYGSTGGYGSSQSSQSSYGQQSS
YPGYGQQPAPSSTSGSYGSSSQSSSYGQPQSGSYSQQPSYGGQQQSYGQQQSYNPPQGYGQQNQYNSSSGGGGGGGGGGNYGQDQSSMSSGGGS
GGGYGNQDQSGGGGSGGYGQQDRGGRGRGGSGGGGGGGGGYNRSSGGYEPRGRGGGRGGRGGMGGSDRGGFNKFGGPRDQGSRHDSEQD
NSDNNTIFVQGLGENVTIESVADYFKQIGIIKTNKKTGQPMINLYTDRETGKLKGEATVSFDDPPSAKAAIDWFDGKEFSGNPIKVSFATRRADFNRGGGN
GRGGRGRGGPMGRGGYGGGGSGGGGRGGFPSGGGGGGGQQRAGDWKCPNPTCENMNFSWRNECNQCKAPKPDGPGGGPGGSHMGGNYGD
DRRGGRGGYDRGGYRGRGGDRGGFRGGRGGGDRGGFGPGKMDSRGEHRQDRRERPY

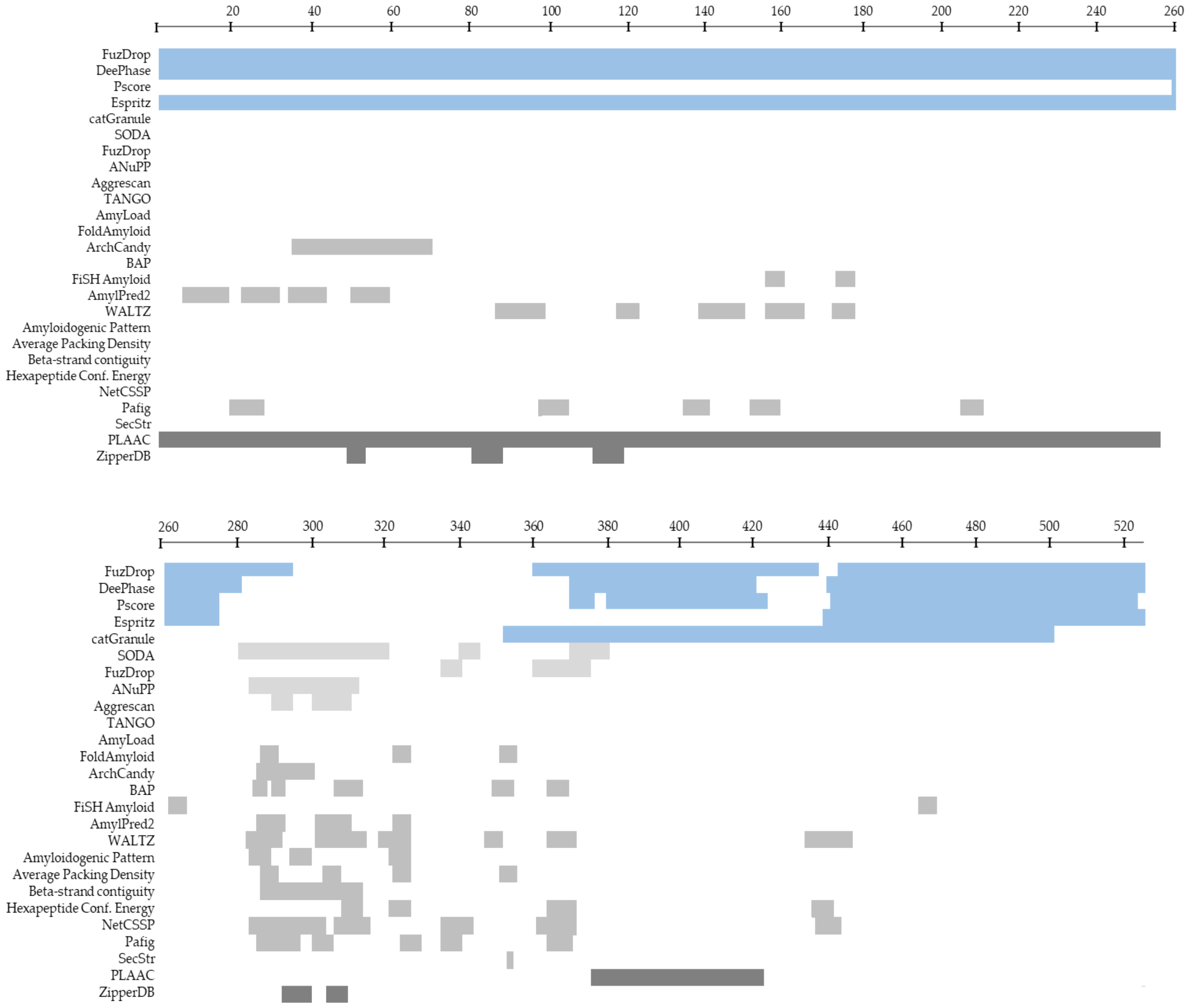

(b) PDB: **5YVG**

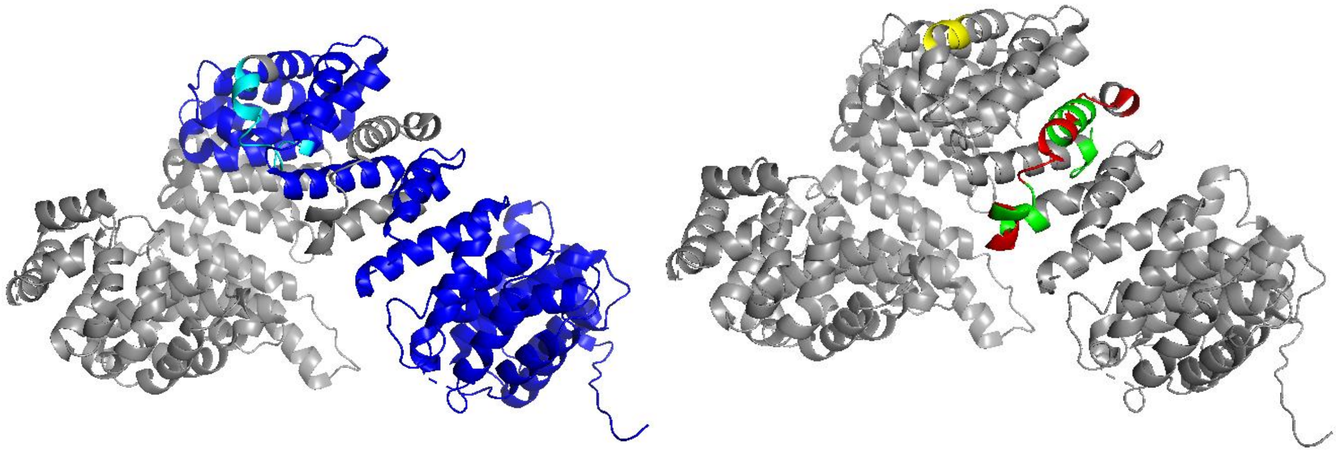

Condensation prone regions

1 10 20 30 40 50 60 70 80 90 100
MASNDYTQQATQSYGAYPTQPGQGYSQQSSQPYGQQSYSGYSQSTDTSGYGQSSYSSYGQSQNTGYGTQSTPQGYGSTGGYGSSQSSQSSYGQQSSYPGY
. 110 120 130 140 150 160 170 180 190 200
GQQPAPSSTSGSYGSSSQSSSYGQPQSGSYSQQPSYGGQQQSYGQQQSYNPPQGYGQQNQYNSSSGGGGGGGGGGNYGQDQSSMSSGGGSGGGYGNQDQS
. 210 220 230 240 250 260 270 280 290 300
GGGGSGGYGQQDRGGRGRGGSGGGGGGGGGGYNRSSGGYEPRGRGGGRGGRGGMGGSDRGGFNKFGGPRDQGSRHDSEQDNSDNNTIFVQGLGENVTIES
. 310 320 330 340 350 360 370 380 390 400
VADYFKQIGIIKTNKKTGQPMINLYTDRETGKLKGEATVSFDDPPSAKAAIDWFDGKEFSGNPIKVSFATRRADFNRGGGNGRGGRGRGGPMGRGGYGGG
. 410 420 430 440 450 460 470 480 490
GSGGGGRGGFPSGGGGGGGQQRAGDWKCPNPTCENMNFSWRNECNQCKAPKPDGPGGGPGGSHMGGNYGDDRRGGRGGYDRGGYRGRGGD
. 500 510 520
RGGFRGGRGGGDRGGFGPGKMDSRGEHRQDRRERPY

Aggregation/amyloid prone regions

1 10 20 30 40 50 60 70 80 90 100
MASNDYTQQATQSYGAYPTQPGQGYSQQSSQPYGQQSYSGYSQSTDTSGYGQSSYSSYGQSQNTGYGTQSTPQGYGSTGGYGSSQSSQSSYGQQSSYPGY
. 110 120 130 140 150 160 170 180 190 200
GQQPAPSSTSGSYGSSSQSSSYGQPQSGSYSQQPSYGGQQQSYGQQQSYNPPQGYGQQNQYNSSSGGGGGGGGGGNYGQDQSSMSSGGGSGGGYGNQDQS
. 210 220 230 240 250 260 270 280 290 300
GGGGSGGYGQQDRGGRGRGGSGGGGGGGGGGYNRSSGGYEPRGRGGGRGGRGGMGGSDRGGFNKFGGPRDQGSRHDSEQDNSDNNTIFVQGLGENVTIES
. 310 320 330 340 350 360 370 380 390 400
VADYFKQIGIIKTNKKTGQPMINLYTDRETGKLKGEATVSFDDPPSAKAAIDWFDGKEFSGNPIKVSFATRRADFNRGGGNGRGGRGRGGPMGRGGYGGG
. 410 420 430 440 450 460 470 480 490
GSGGGGRGGFPSGGGGGGGQQRAGDWKCPNPTCENMNFSWRNECNQCKAPKPDGPGGGPGGSHMGGNYGDDRRGGRGGYDRGGYRGRGGD
. 500 510 520
RGGFRGGRGGGDRGGFGPGKMDSRGEHRQDRRERPY

Figure S12. TAU: (a) Predictions of condensation, aggregation, amyloid and prion prone regions (b) visualization of regions in 3D protein structure.

(a)

>TAU,P10636

MAEPRQEFEVMEDHAGTYGLGDRKDQGGYTMHQDQEGDTDAGLKESPLQTPTEDGSEEPGSETSDAKSTPTAEDVTAPLVDEGAPGKQAAAQPHTE
IPEGTTAEEAGIGDTPSLEDEAAGHVTQEPESGKVVQEGFLREPGPPGLSHQLMSGMPGAPLLPEGPREATRQPSGTGPEDTEGGRHAPELLKHQLLGD
LHQEGPPLKGAGGKERPGSKEEVDEDRDVDESSPQDSPPSKASPAQDGRPPQTAAREATSIPGFPAEGAIPLPVDFLSKVSTEIPASEPDGPSVGRAKGQ
DAPLEFTFHVEITPNVQKEQAHSEEHLGRAAFPGAPGEGPEARGPSLGEDTKEADLPEPSEKQPAAAPRGKPVSRVPQLKARMVSKSKDGTGSDDKKAK
TSTRSSAKTLKNRPCLSPKHPTPGSSDPLIQPSSPAVCPEPPSSPKYVSSVTSRTGSSGAKEMKLKGADGKTKIATPRGAAPPGQKGQANATRIPAKTPPAP
KTPPSSGEPPKSGDRSGYSSPGSPGTPGSRSRTPSLPTPPTREPKKVAVVRTPPKSPSSAKSRLQTAPVPMPDLKNVKSKIGSTENLKHQPGGGKVQIINKK
LDLSNVQSKCGSKDNIKHVPGGGSVQIVYKPVDLSKVTSKCGSLGNIHHKPGGGQVEVKSEKLDFKDRVQSKIGSLDNITHVPGGGNKKIETHKLTFRENA
KAKTDHGAEIVYKSPVVSGDTSPRHLSNVSSTGSIDMVDSPQLATLADEVSASLAKQGL

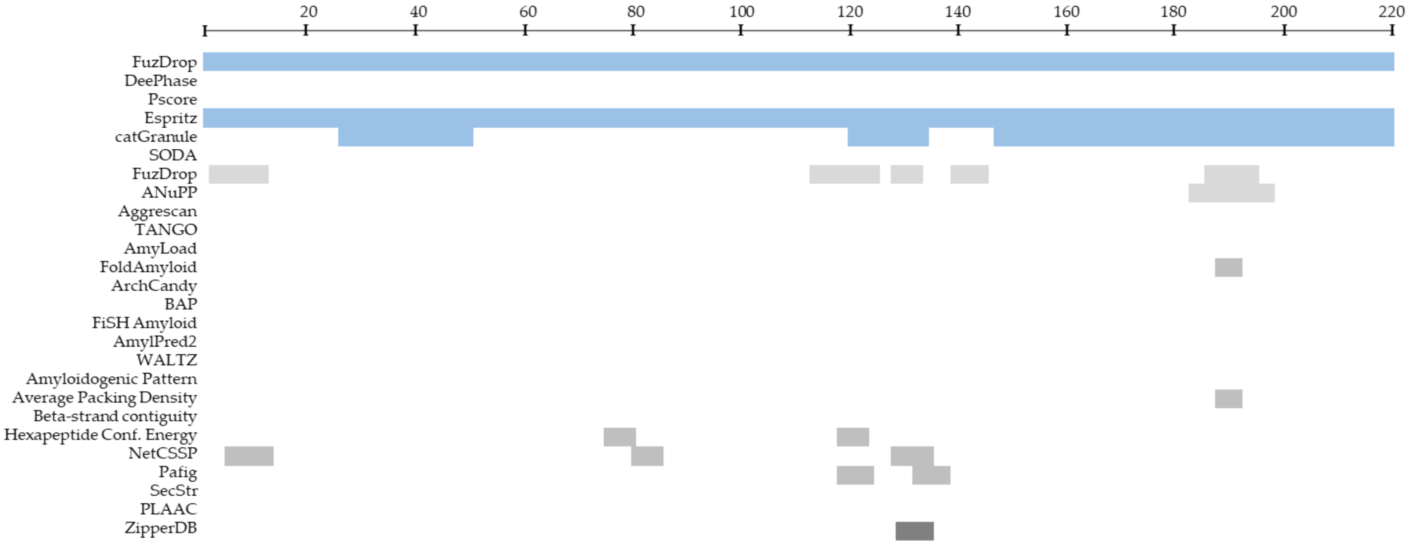

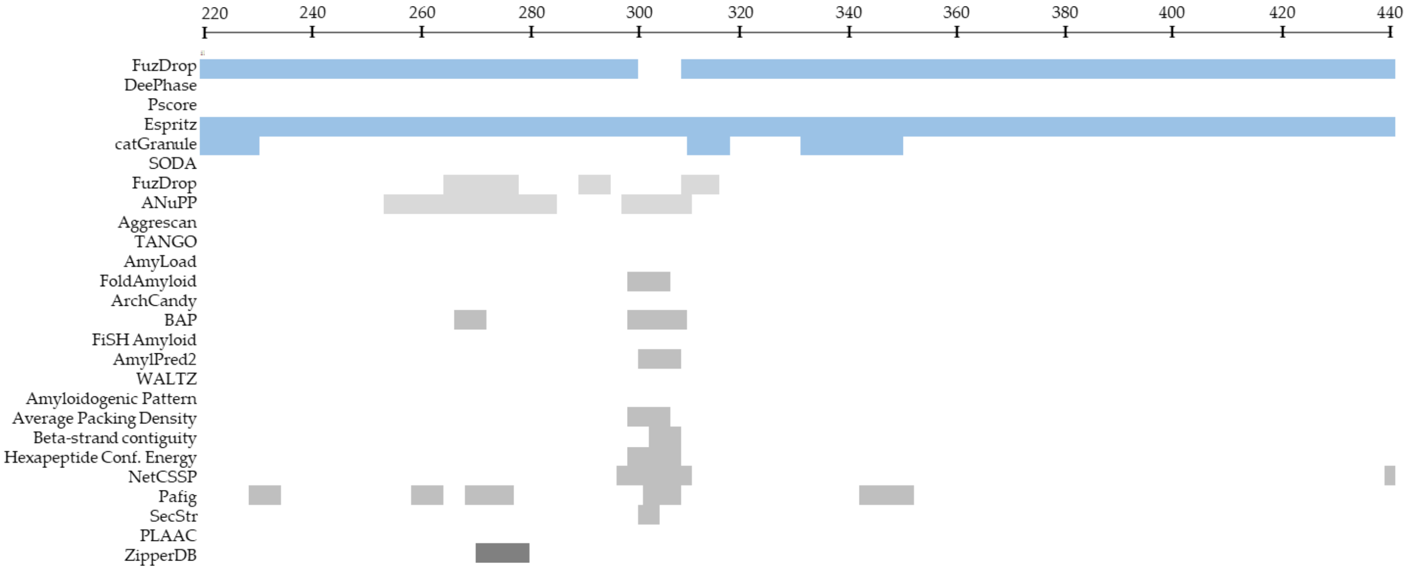

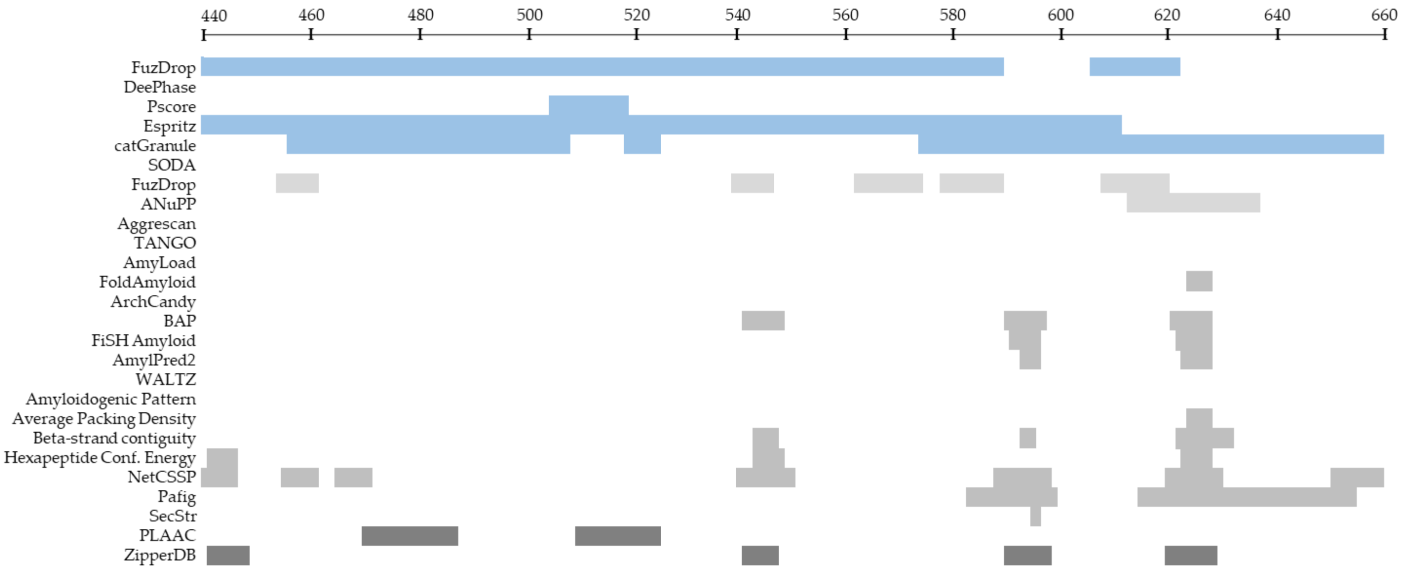
440
460
480
500
520
540
560
580
600
620
640
660
FuzDrop
DeePhase
Pscore
Espritz
catGranule
SODA
FuzDrop
ANuPP
Aggrescan
TANGO
AmyLoad
FoldAmyloid
ArchCandy
BAP
FiSH Amyloid
AmylPred2
WALTZ
Amyloidogenic Pattern
Average Packing Density
Beta-strand contiguity
Hexapeptide Conf. Energy
NetCSSP
Pafig
SecStr
PLAAC
ZipperDB

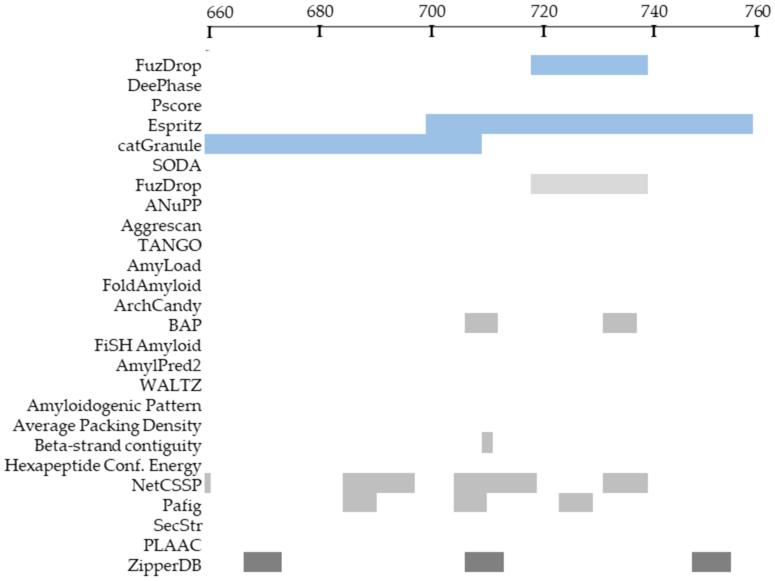
660
680
700
720
740
760
FuzDrop
DeePhase
Pscore
Espritz
catGranule
SODA
FuzDrop
ANuPP
Aggrescan
TANGO
AmyLoad
FoldAmyloid
ArchCandy
BAP
FiSH Amyloid
AmylPred2
WALTZ
Amyloidogenic Pattern
Average Packing Density
Beta-strand contiguity
Hexapeptide Conf. Energy
NetCSSP
Pafig
SecStr
PLAAC
ZipperDB

(b) AF:p10636-9-f1

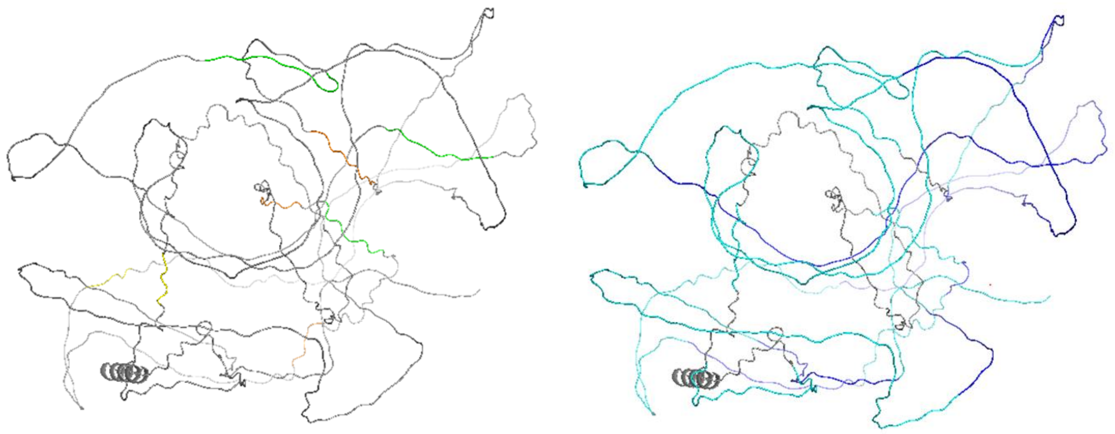

Condensation prone regions

1 10 20 30 40 50 60 70 80 90 100
MAEPRQEFEVMEDHAGTYGLGDRKDQGGYTMHQDQEGDTDAGLKESPLQTPTEDGSEEPGSETSDAKSTPTAEDVTAPLVDEGAPGKQAAAQPHTEIPEG
. 110 120 130 140 150 160 170 180 190 200
TTAEEAGIGDTPSLEDEAAGHVTQEPESGKVVQEGFLREPGPPGLSHQLMSGMPGAPLLPEGPREATRQPSGTGPEDTEGGRHAPELLKHQLLGDLHQEG
. 210 220 230 240 250 260 270 280 290 300
PPLKGAGGKERPGSKEEVDEDRDVDESSPQDSPPSKASPAQDGRPPQTAAREATSIPGFPAEGAIPLPVDFLSKVSTEIPASEPDGPSVGRAKGQDAPLE
. 310 320 330 340 350 360 370 380 390 400
FTFHVEITPNVQKEQAHSEEHLGRAAFPGAPGEGPEARGPSLGEDTKEADLPEPSEKQPAAAPRGKPVSRVPQLKARMVSKSKDGTGSDDKKAKTSTRSS
. 410 420 430 440 450 460 470 480 490 500
AKTLKNRPCLSPKHPTPGSSDPLIQPSSPAVCPEPPSSPKYVSSVTSRTGSSGAKEMKLKGADGKTKIATPRGAAPPGQKGQANATRIPAKTPPAPKTPP
. 510 520 530 540 550 560 570 580 590 600
SSGEPPKSGDRSGYSSPGSPGTPGSRSRTPSLPTPPTREPKKVAVVRTPPKSPSSAKSRLQTAPVPMPDLKNVKSKIGSTENLKHQPGGGKVQIINKKLD
. 610 620 630 640 650 660 670 680 690 700
LSNVQSKCGSKDNIKHVPGGGSVQIVYKPVDLSKVTSKCGSLGNIHHKPGGGQVEVKSEKLDFKDRVQSKIGSLDNITHVPGGGNKKIETHKLTFRENAK
. 710 720 730 740 750
AKTDHGAEIVYKSPVVSGDTSPRHLSNVSSTGSIDMVDSPQLATLADEVSASLAKQGL

Aggregation/amyloid prone regions

1 10 20 30 40 50 60 70 80 90 100
MAEPRQEFEVMEDHAGTYGLGDRKDQGGYTMHQDQEGDTDAGLKESPLQTPTEDGSEEPGSETSDAKSTPTAEDVTAPLVDEGAPGKQAAAQPHTEIPEG
. 110 120 130 140 150 160 170 180 190 200
TTAEEAGIGDTPSLEDEAAGHVTQEPESGKVVQEGFLREPGPPGLSHQLMSGMPGAPLLPEGPREATRQPSGTGPEDTEGGRHAPELLKHQLLGDLHQEG
. 210 220 230 240 250 260 270 280 290 300
PPLKGAGGKERPGSKEEVDEDRDVDESSPQDSPPSKASPAQDGRPPQTAAREATSIPGFPAEGAIPLPVDFLSKVSTEIPASEPDGPSVGRAKGQDAPLE
. 310 320 330 340 350 360 370 380 390 400
FTFHVEITPNVQKEQAHSEEHLGRAAFPGAPGEGPEARGPSLGEDTKEADLPEPSEKQPAAAPRGKPVSRVPQLKARMVSKSKDGTGSDDKKAKTSTRSS
. 410 420 430 440 450 460 470 480 490 500
AKTLKNRPCLSPKHPTPGSSDPLIQPSSPAVCPEPPSSPKYVSSVTSRTGSSGAKEMKLKGADGKTKIATPRGAAPPGQKGQANATRIPAKTPPAPKTPP
. 510 520 530 540 550 560 570 580 590 600
SSGEPPKSGDRSGYSSPGSPGTPGSRSRTPSLPTPPTREPKKVAVVRTPPKSPSSAKSRLQTAPVPMPDLKNVKSKIGSTENLKHQPGGGKVQIINKKLD
. 610 620 630 640 650 660 670 680 690 700
LSNVQSKCGSKDNIKHVPGGGSVQIVYKPVDLSKVTSKCGSLGNIHHKPGGGQVEVKSEKLDFKDRVQSKIGSLDNITHVPGGGNKKIETHKLTFRENAK
. 710 720 730 740 750
AKTDHGAEIVYKSPVVSGDTSPRHLSNVSSTGSIDMVDSPQLATLADEVSASLAKQGL

Figure S13. TDP-43: (a) Predictions of condensation, aggregation, amyloid and prion prone regions (b) visualization of regions in 3D protein structure.

(a)

>TDP-43,Q13148

MSEYIRVTEDENDEPIEIPSEDDGTVLLSTVTAQFPGACGLRYRNPVSQCMRGVRLVEGILHAPDAGWGNLVYVVNYPKDNKRKMDETDASSAVKVKR
AVQKTSDLIVLGLPWKTTEQDLKEYFSTFGEVLMVQVKKDLKTGHSKGFGFVRFTEYETQVKVMSQRHMIDGRWCDCKLPNSKQSQDEPLRSRKVFVG
RCTEDMTEDELREFFSQYGDVMDVFIPKPFRAFAFVTFADDQIAQSLCGEDLIIKGISVHISNAEPKHNSNRQLERSGRFGGNPGGFGNQGGFGNSRGG
GAGLGNNQGSNMGGGMNFGAFSINPAMMAAAQAALQSSWGMMGMLASQQNQSGPSGNNQNQGNMQREPNQAFGSGNNSYSGSNSGAAIG
WGSASNAGSGSGFNGGFGSSMDSKSSGWGM

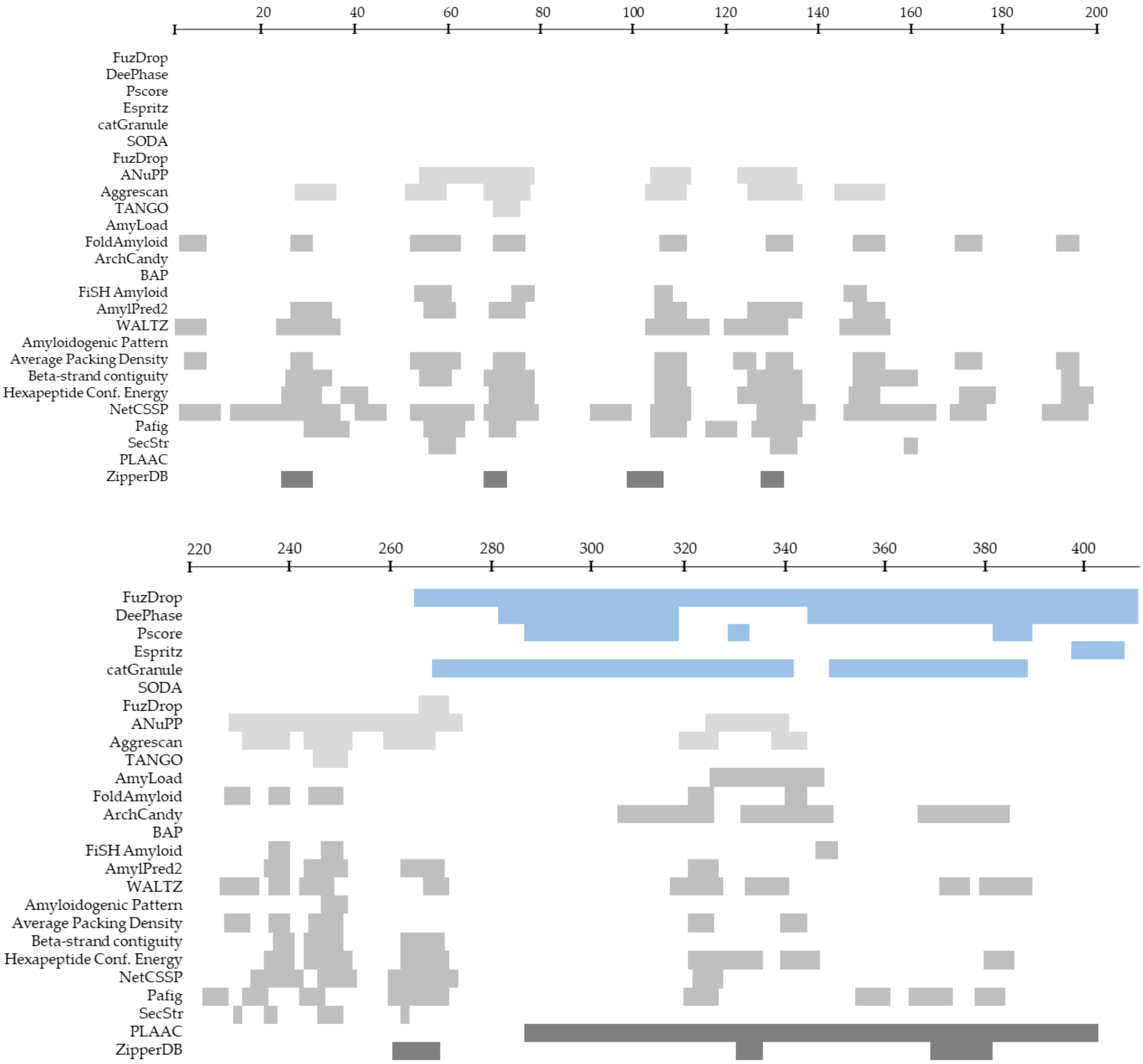

(b) AF: **Q13148**

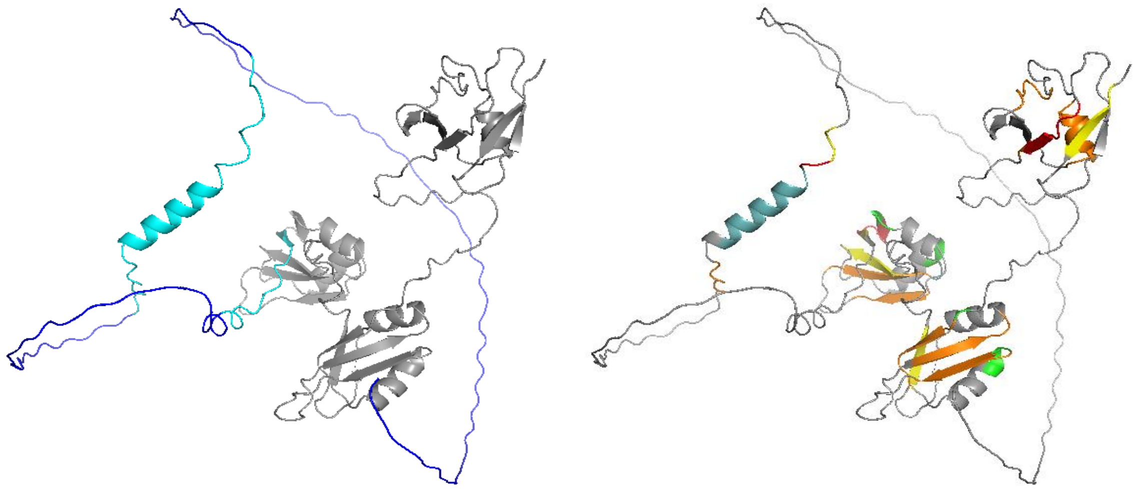

Condensation prone regions

1 10 20 30 40 50 60 70 80 90 100
MSEYIRVTEDENDEPIEIPSEDDGTVLLSTVTAQFPGACGLRYRNPVSQCMRGVRLVEGILHAPDAGWGNLVYVVNYPKDNKRKMDETDASSAVKVKRAV
. 110 120 130 140 150 160 170 180 190 200
QKTSDLIVLGLPWKTTEQDLKEYFSTFGEVLMVQVKKDLKTGHSKGFGFVRFTEYETQVKVMSQRHMIDGRWCDCKLPNSKQSQDEPLRSRKVFVGRCTE
. 210 220 230 240 250 260 270 280 290 300
DMTEDELREFFSQYGDVMDVFIPKPFRAFAFVTFADDQIAQSLCGEDLIIKGISVHISNAEPKHNSNRQLERSGRFGGNPGGFGNQGGFGNSRGGGAGLG
. 310 320 330 340 350 360 370 380 390
NNQGSNMGGGMNFGAFSINPAMMAAAQAALQSSWGMMGMLASQQNQSGPSGNNQNQGNMQREPNQAFGSGNNSYSGSNSGAAIGWGSASN
. 400 410
AGSGSGFNGGFGSSMDSKSSGWGM

Aggregation/amyloid prone regions

1 10 20 30 40 50 60 70 80 90 100
MSEYIRVTEDENDEPIEIPSEDDGTVLLSTVTAQFPGACGLRYRNPVSQCMRGVRLVEGILHAPDAGWGNLVYVVNYPKDNKRKMDETDASSAVKVKRAV
. 110 120 130 140 150 160 170 180 190 200
QKTSDLIVLGLPWKTTEQDLKEYFSTFGEVLMVQVKKDLKTGHSKGFGFVRFTEYETQVKVMSQRHMIDGRWCDCKLPNSKQSQDEPLRSRKVFVGRCTE
. 210 220 230 240 250 260 270 280 290 300
DMTEDELREFFSQYGDVMDVFIPKPFRAFAFVTFADDQIAQSLCGEDLIIKGISVHISNAEPKHNSNRQLERSGRFGGNPGGFGNQGGFGNSRGGGAGLG
. 310 320 330 340 350 360 370 380 390
NNQGSNMGGGMNFGAFSINPAMMAAAQAALQSSWGMMGMLASQQNQSGPSGNNQNQGNMQREPNQAFGSGNNSYSGSNSGAAIGWGSASN
. 400 410
AGSGSGFNGGFGSSMDSKSSGWGM

Figure S14.DISC1: (a) Predictions of condensation, aggregation, amyloid and prion prone regions (b) visualization of regions in 3D protein structure.

(a)

>DISC1,Q9NRI5

MPGGGPQGAPAAAGGGGVSHRAGSRDCLPPAACFRRRRLARRPGYMRSSTGPGIGFLSPAVGTLFRFPGGVSGEESHHSESRARQCGLDSRGLLVRSP
VSKSAAAPTVTSVRGTSAHFGIQLRGGTRLPDRLSWPCGPGSAGWQQEFAAMDSSETLDASWEAACSDGARRVRAAGSLPSAELSSNSCSPGCGPEVP
PTPPGSHSAFTSSFSFIRLSLGSAGERGEAEGCPPSREAESHCQSPQEMGAKAASLDGPHEDPRCLSRPFSLLATRVSADLAQAARNSSRPERDMHSLPD
MDPGSSSSLDPSLAGCGGDGSSGSGDAHSWDTLLRKWEPVLRDCLLRNRRQMEVISLRLKLQKLQEDAVENDDYDKAETLQQRLEDLEQEKISLHFQLP
SRQPALSSFLGHLAAQVQAALRRGATQQASGDDTHTPLRMEPRLLEPTAQDSLHVSITRRDWLLQEKQQLQKEIEALQARMFVLEAKDQQLRREIEEQE
QQLQWQGCDLTPLVGQLSLGQLQEVSKALQDTLASAGQIPFHAEPPETIRSLQERIKSLNLSLKEITTKVCMSEKFCSTLRKKVNDIETQLPALLEAKMHAIS
GNHFWTAKDLTEEIRSLTSEREGLEGLLSKLLVLSSRNVKKLGSVKEDYNRLRREVEHQETAYETSVKENTMKYMETLKNKLCSCKCPLLGKVWEADLEAC
RLLIQSLQLQEARGSLSVEDERQMDDLEGAAPPIPPRLHSEDKRKTPLKVLEEWKTHLIPSLHCAGGEQKEESYILSAELGEKCEDIGKKLLYLEDQLHTAIHS
HDEDLIQSLRRELQMVKETLQAMILQLQPAKEAGEREAAASCMTAGVHEAQA

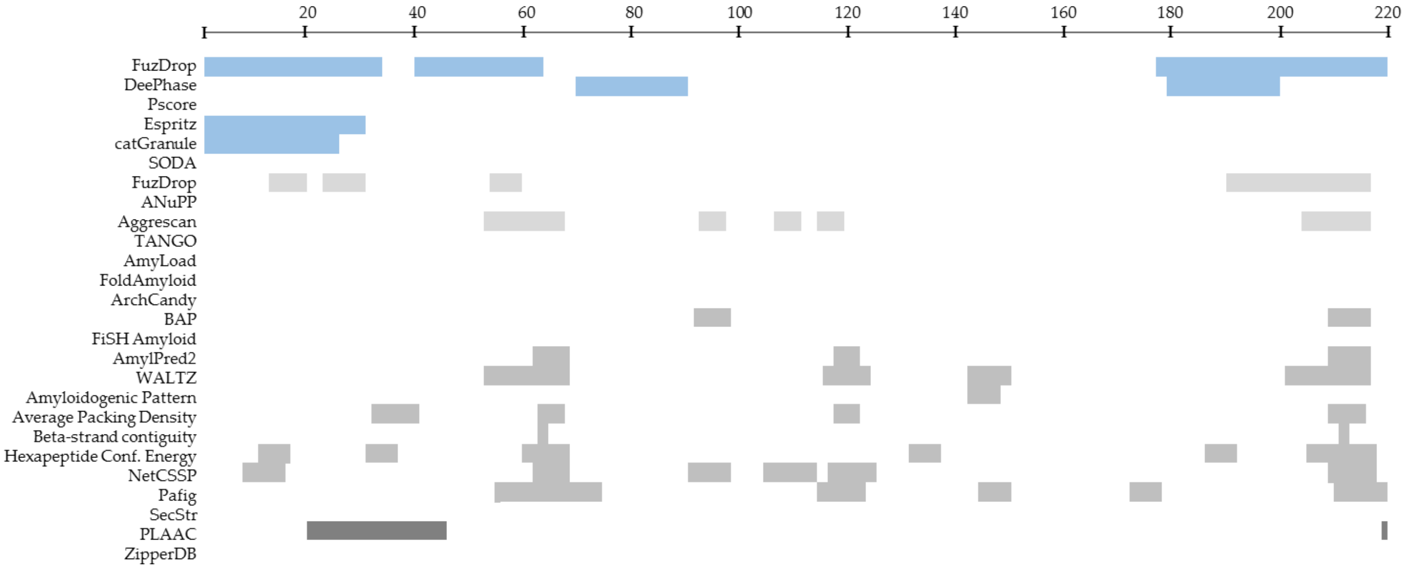

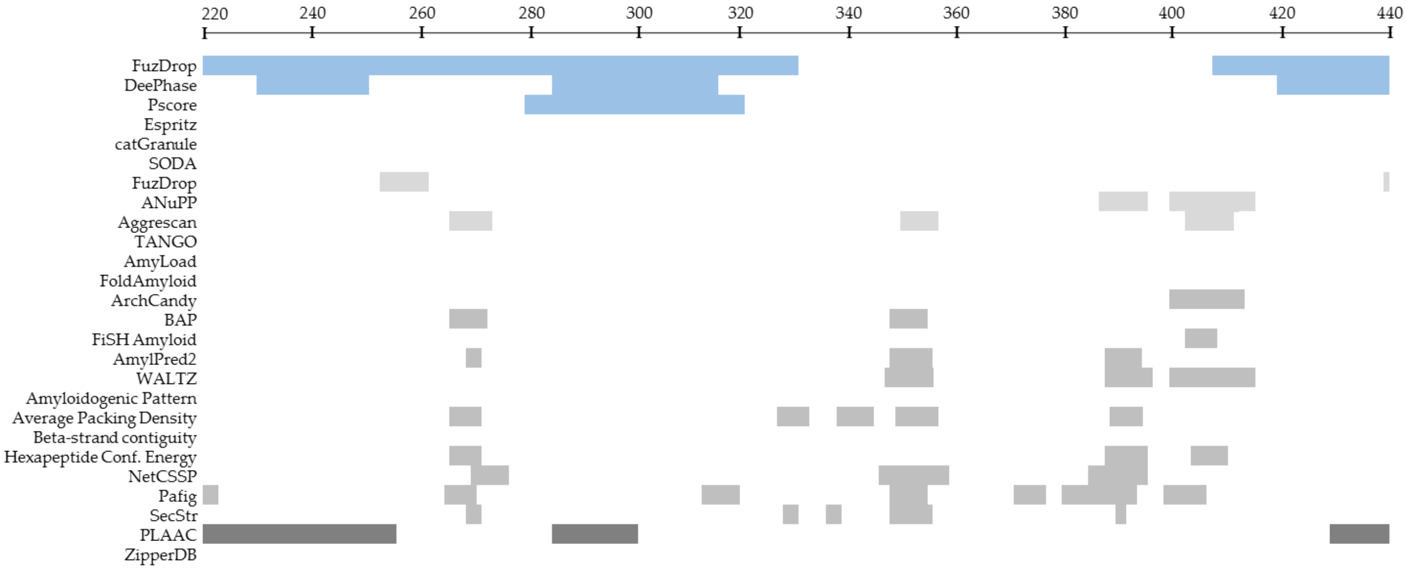

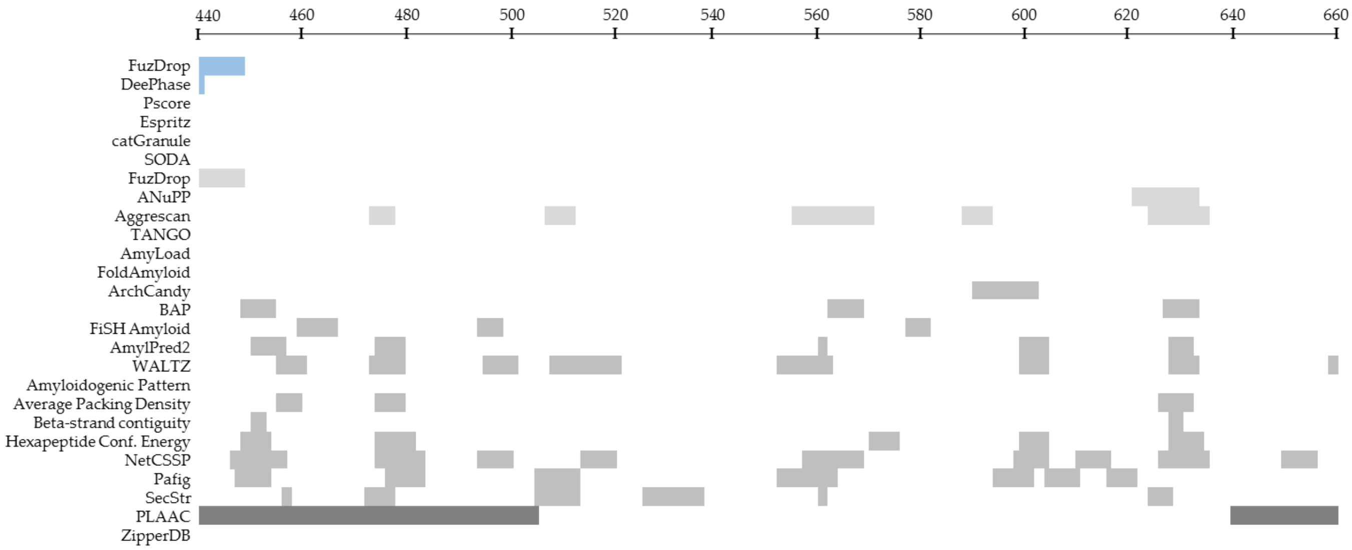
440
460
480
500
520
540
560
580
600
620
640
660
FuzDrop
DeePhase
Pscore
Espritz
catGranule
SODA
FuzDrop
ANuPP
Aggrescan
TANGO
AmyLoad
FoldAmyloid
ArchCandy
BAP
FiSH Amyloid
AmylPred2
WALTZ
Amyloidogenic Pattern
Average Packing Density
Beta-strand contiguity
Hexapeptide Conf. Energy
NetCSSP
Pafig
SecStr
PLAAC
ZipperDB

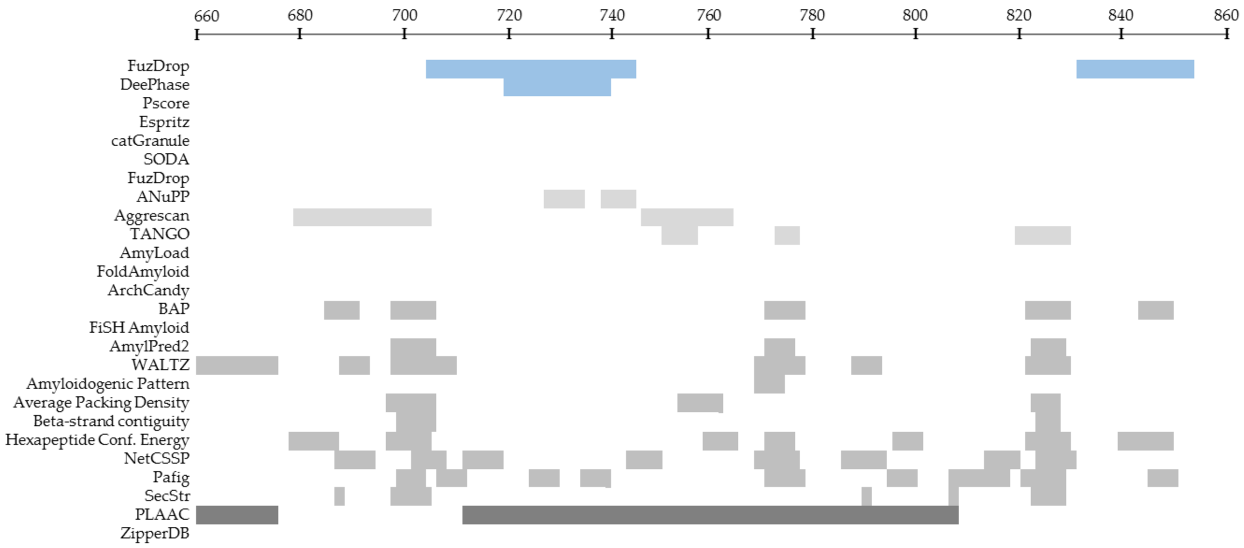
660
680
700
720
740
760
780
800
820
840
860
FuzDrop
DeePhase
Pscore
Espritz
catGranule
SODA
FuzDrop
ANuPP
Aggrescan
TANGO
AmyLoad
FoldAmyloid
ArchCandy
BAP
FiSH Amyloid
AmylPred2
WALTZ
Amyloidogenic Pattern
Average Packing Density
Beta-strand contiguity
Hexapeptide Conf. Energy
NetCSSP
Pafig
SecStr
PLAAC
ZipperDB

(b) AF: **Q13148**

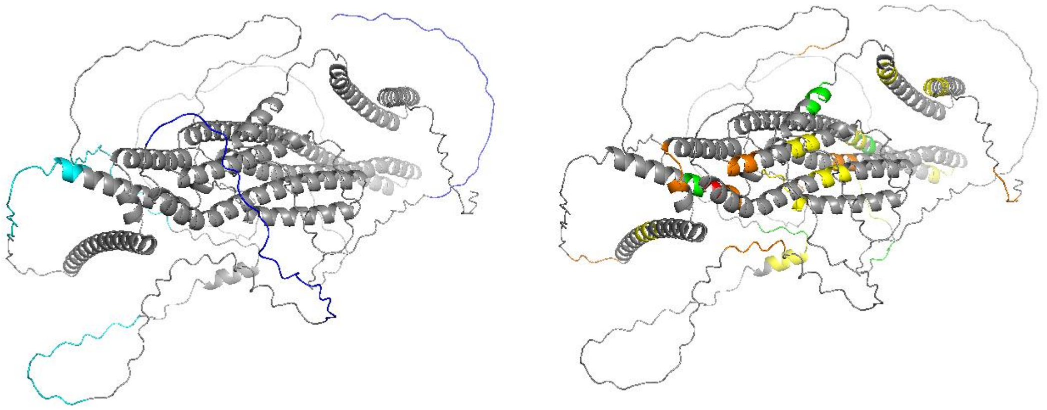

Condensation prone regions

1 10 20 30 40 50 60 70 80 90 100
MPGGGPQGAPAAAGGGGVSHRAGSRDCLPPAACFRRRRLARRPGYMRSSTGPGIGFLSPAVGTLFRFPGGVSGEESHHSESRARQCGLDSRGLLVRSPVS
. 110 120 130 140 150 160 170 180 190 200
KSAAAPTVTSVRGTSAHFGIQLRGGTRLPDRLSWPCGPGSAGWQQEFAAMDSSETLDASWEAACSDGARRVRAAGSLPSAELSSNSCSPGCGPEVPPTPP
. 210 220 230 240 250 260 270 280 290 300
GSHSAFTSSFSFIRLSLGSAGERGEAEGCPPSREAESHCQSPQEMGAKAASLDGPHEDPRCLSRPFSLLATRVSADLAQAARNSSRPERDMHSLPDMDPG
. 310 320 330 340 350 360 370 380 390 400
SSSSLDPSLAGCGGDGSSGSGDAHSWDTLLRKWEPVLRDCLLRNRRQMEVISLRLKLQKLQEDAVENDDYDKAETLQQRLEDLEQEKISLHFQLPSRQPA
. 410 420 430 440 450 460 470 480 490 500
LSSFLGHLAAQVQAALRRGATQQASGDDTHTPLRMEPRLLEPTAQDSLHVSITRRDWLLQEKQQLQKEIEALQARMFVLEAKDQQLRREIEEQEQQLQWQ
. 510 520 530 540 550 560 570 580 590 600
GCDLTPLVGQLSLGQLQEVSKALQDTLASAGQIPFHAEPPETIRSLQERIKSLNLSLKEITTKVCMSEKFCSTLRKKVNDIETQLPALLEAKMHAISGNH .
. 610 620 630 640 650 660 670 680 690 700
FWTAKDLTEEIRSLERETSGLEGLLSKLLVLSSRNVKKLGSVKEDYNRLRREVEHQETAYETSVKENTMKYMETLKNKLCSCKCPLLGKVWEADLEACRL .
. 710 720 730 740 750 760 770 780 790 800
LIQSLQLQEARGSLSVEDERQMDDLEGAAPPIPPRLHSEDKRKTPLKVLEEWKTHLIPSLHCAGGEQKEESYILSAELGEKCEDIGKKLLYLEDQLHTAI .
. 810 820 830 840 850
HSHDEDLIQSLRRELQMVKETLQAMILQLQPAKEAGEREAAASCMTAGVHEAQA

Aggregation/amyloid prone regions

1 10 20 30 40 50 60 70 80 90 100
MPGGGPQGAPAAAGGGGVSHRAGSRDCLPPAACFRRRRLARRPGYMRSSTGPGIGFLSPAVGTLFRFPGGVSGEESHHSESRARQCGLDSRGLLVRSPVS
. 110 120 130 140 150 160 170 180 190 200
KSAAAPTVTSVRGTSAHFGIQLRGGTRLPDRLSWPCGPGSAGWQQEFAAMDSSETLDASWEAACSDGARRVRAAGSLPSAELSSNSCSPGCGPEVPPTPP
. 210 220 230 240 250 260 270 280 290 300
GSHSAFTSSFSFIRLSLGSAGERGEAEGCPPSREAESHCQSPQEMGAKAASLDGPHEDPRCLSRPFSLLATRVSADLAQAARNSSRPERDMHSLPDMDPG
. 310 320 330 340 350 360 370 380 390 400
SSSSLDPSLAGCGGDGSSGSGDAHSWDTLLRKWEPVLRDCLLRNRRQMEVISLRLKLQKLQEDAVENDDYDKAETLQQRLEDLEQEKISLHFQLPSRQPA
. 410 420 430 440 450 460 470 480 490 500
LSSFLGHLAAQVQAALRRGATQQASGDDTHTPLRMEPRLLEPTAQDSLHVSITRRDWLLQEKQQLQKEIEALQARMFVLEAKDQQLRREIEEQEQQLQWQ
. 510 520 530 540 550 560 570 580 590 600
GCDLTPLVGQLSLGQLQEVSKALQDTLASAGQIPFHAEPPETIRSLQERIKSLNLSLKEITTKVCMSEKFCSTLRKKVNDIETQLPALLEAKMHAISGNH .
. 610 620 630 640 650 660 670 680 690 700
FWTAKDLTEEIRSLERETSGLEGLLSKLLVLSSRNVKKLGSVKEDYNRLRREVEHQETAYETSVKENTMKYMETLKNKLCSCKCPLLGKVWEADLEACRL .
. 710 720 730 740 750 760 770 780 790 800
LIQSLQLQEARGSLSVEDERQMDDLEGAAPPIPPRLHSEDKRKTPLKVLEEWKTHLIPSLHCAGGEQKEESYILSAELGEKCEDIGKKLLYLEDQLHTAI .
. 810 820 830 840 850
HSHDEDLIQSLRRELQMVKETLQAMILQLQPAKEAGEREAAASCMTAGVHEAQA

Figure S15. FABP3: (a) Predictions of condensation, aggregation, amyloid and prion prone regions (b) visualization of regions in 3D protein structure.

(a)

> FABP3, P05413

MVDAFLGTWKLVDSKNFDDYMKSLGVGFATRQVASMTKPTTIIEKNGDILTLKTHSTFKNTEISFKLGVEFDETTADDRKVKSIVTLDGGKL VHLQKWDGQETTLVRELIDGKLILTLTHGTAVCTRTYEKEA

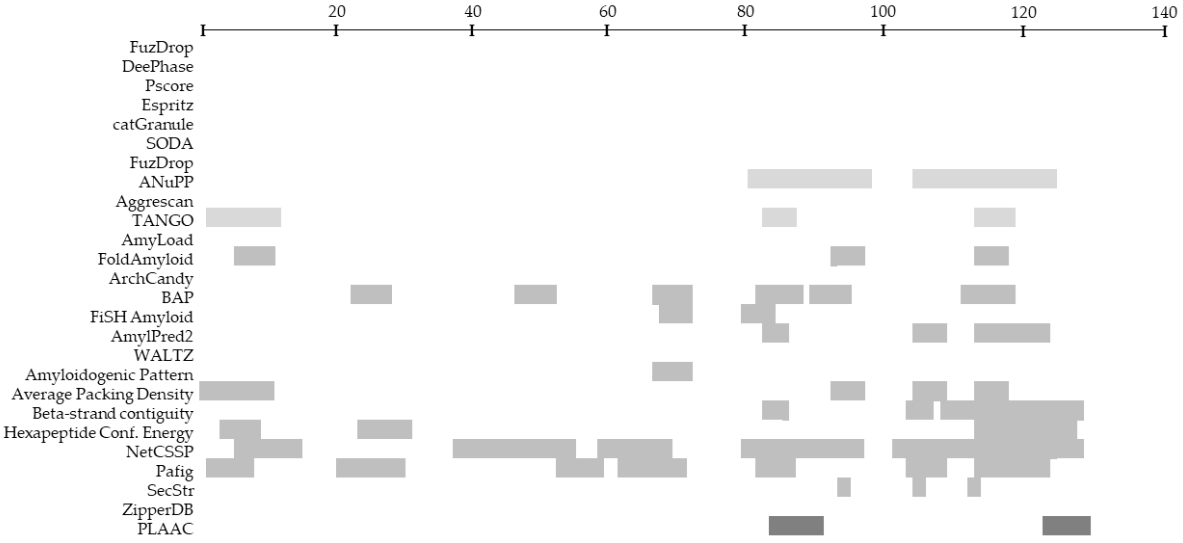

(b) **PDB: 1G5W**

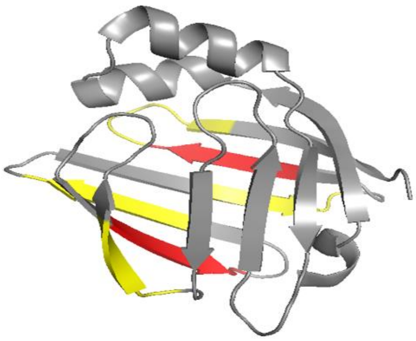

Aggregation/amyloid prone regions

1 10 20 30 40 50 60 70 80 90 100

MVDAFLGTWKLVDSKNFDDYMKSLGVGFATRQVASMTKPTTIIEKNGDILTLKTHSTFKNTEISFKLGVEFDETTADDRKVKSIVTLDGGKLVHLQKWDG

. 110 120 130

QETTLVRELIDGKLILTLTHGTAVCTRTYEKEA

Figure S16. LIMP2: (a) Predictions of condensation, aggregation, amyloid and prion prone regions (b) visualization of regions in 3D protein structure.

(a)

> LIMP2, Q14108

MGRCCFYTAGTLSLLLLVTSVTLLVARVFQKAVDQSIEKKIVLRNGTEAFDSWEKPPLPVYTQFYFFNVTNPEEILRGETPRVEEVGPYTYREL
RNKANIQFGDNGTTISAVSNKAYVFERDQSVGDPKIDLIRTLNIPVLTVIEWSQVHFLREIIEAMLKAYQQKLFVTHTVDELLWGYKDEILSLI
HVFRPDISPYFGLFYEKNGTNDGDYVFLTGEDSYLNFTKIVEWNGKTSLDWWITDKCNMINGTDGDSFHPLITKDEVLYVFPSDFCRSVYIT
FSDYESVQGLPAFRYKVPAEILANTSDNAGFCIPEGNCLGSGVLNVSICKNGAPIIMSFPHFYQADERFVSAIEGMHPNQEDHETFVDINPL
TGIILKAAKRFQINIYVKKLDDFVETGDIRTMVFPVMYLNESVHIDKETASRLKSMINTTLIITNIPYIIMALGVFFGLVFTWLACKGQGSMDE
GTADERAPLIRT

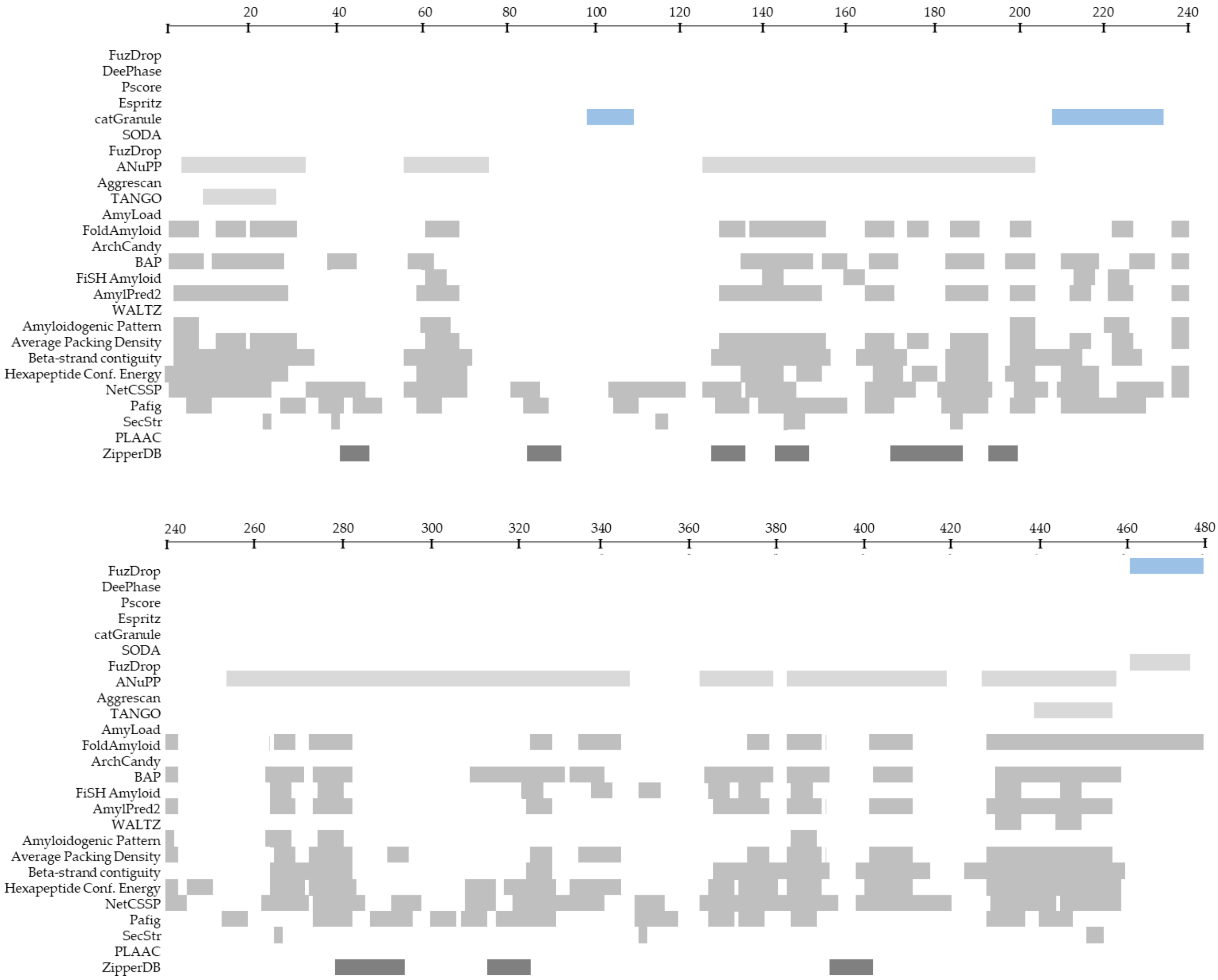

(b) AF: Q14108-F1-model_v6

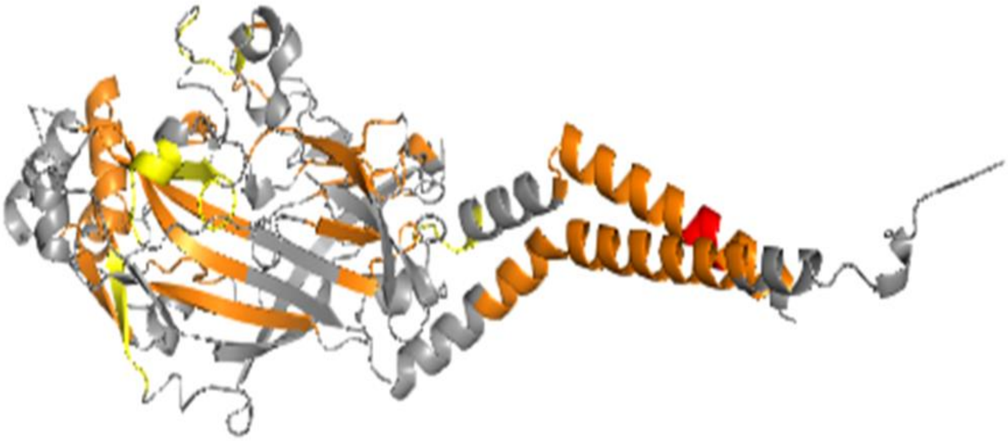

Aggregation/amyloid prone regions

1 10 20 30 40 50 60 70 80 90 100
MGRCCFYTAGTLSLLLLVTSVTLLVARVFQKAVDQSIEKKIVLRNGTEAFDSWEKPPLPVYTQFYFFNVTNPEEILRGETPRVEEVGPYTYRELRNKANI
. 110 120 130 140 150 160 170 180 190 200
QFGDNGTTISAVSNKAYVFERDQSVGDPKIDLIRTLNIPVLTVIEWSQVHFLREIIEAMLKAYQQKLFVTHTVDELLWGYKDEILSLIHVFRPDISPYFG
. 210 220 230 240 250 260 270 280 290 300
LFYEKNGTNDGDYVFLTGEDSYLNFTKIVEWNGKTSLDWWITDKCNMINGTDGDSFHPLITKDEVLYVFPSDFCRSVYITFSDYESVQGLPAFRYKVPAE
. 310 320 330 340 350 360 370 380 390 400
ILANTSDNAGFCIPEGNCLGSGVLNVSICKNGAPIIMSFPHFYQADERFVSAIEGMHPNQEDHETFVDINPLTGIILKAAKRFQINIYVKKLDDFVETGD
. 410 420 430 440 450 460 470
IRTMVFPVMYLNESVHIDKETASRLKSMINTTLIITNIPYIIMALGVFFGLVFTWLACKGQGSMDEGTADERAPLIRT

Figure S17. DTNBP1: (a) Predictions of condensation, aggregation, amyloid and prion prone regions (b) visualization of regions in 3D protein structure.

(a)

> DTNBP1, Q96EV8

MLETLRERLLSVQQDFTSGLKTLSDKSREAKVKSKPRTVPFLPKYSAGLELLSRYEDTWAALHRRAKDCASAGELVDSEVVMLSAHWEKKKT
SLVELQEQLQQLPALIADLESMTANLTHLEASFEEVENNLLHLEDLCGQCELERCKHMQSQQLENYKKNKRKELETFKAELDAEHAQKVLE
MEHTQQMKLKERQKFFEEAFQQDMEQYLSTGYLQIAERREPIGSMSSMEVNVDMLEQMDLMDISDQEALDVFLNSGGEENTVLSPAL
GPESSTCQNEITLQVPNPSELRAKPPSSSSTCTDSATRDISEGGESPVVQSDEEEVQVDTALATSHTDREATPDGGEDSDS

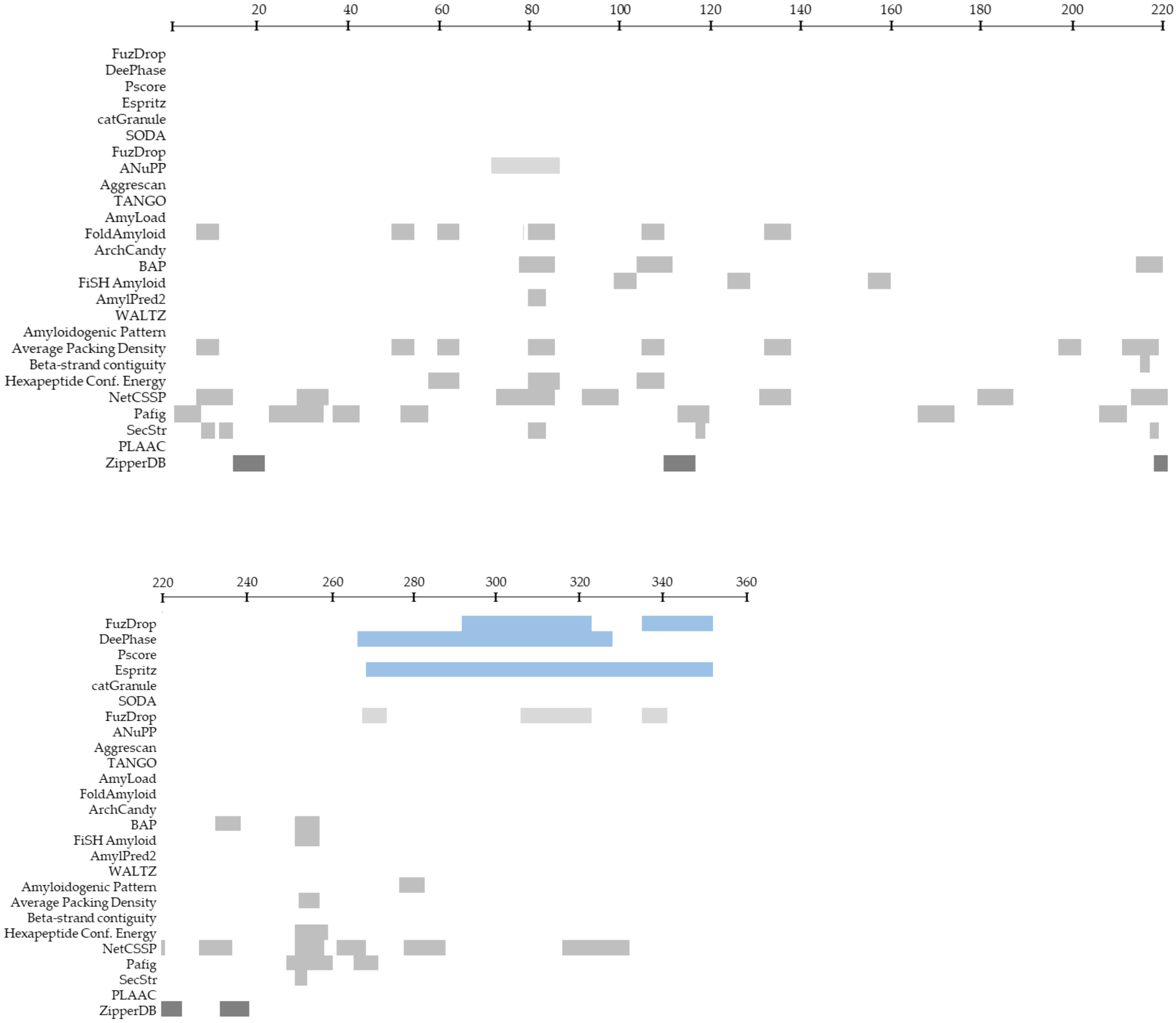

(b) AF-Q96EV8-2-F1

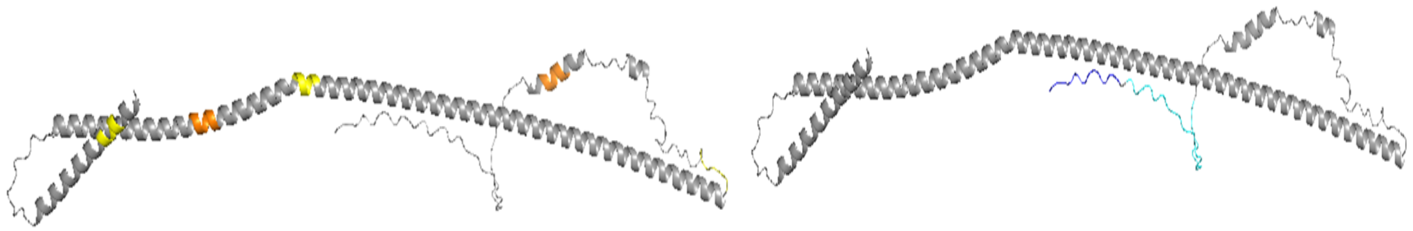

Condensation prone regions

1 10 20 30 40 50 60 70 80 90 100
MLETLRERLLSVQQDFTSGLKTLSDKSREAKVKSKPRTVPFLPKYSAGLELLSRYEDTWAALHRRAKDCASAGELVDSEVVMLSAHWEKKKTSLVELQEQ
. 110 120 130 140 150 160 170 180 190 200
LQQLPALIADLESMTANLTHLEASFEEVENNLLHLEDLCGQCELERCKHMQSQQLENYKKNKRKELETFKAELDAEHAQKVLEMEHTQQMKLKERQKFFE
. 210 220 230 240 250 260 270 280 290 300
EAFQQDMEQYLSTGYLQIAERREPIGSMSSMEVNVDMLEQMDLMDISDQEALDVFLNSGGEENTVLSPALGPESSTCQNEITLQVPNPSELRAKPPSSSS
. 310 320 330 340 350
TCTDSATRDISEGGESPVVQSDEEEVQVDTALATSHTDREATPDGGEDSDS

Aggregation/amyloid prone regions

1 10 20 30 40 50 60 70 80 90 100
MLETLRERLLSVQQDFTSGLKTLSDKSREAKVKSKPRTVPFLPKYSAGLELLSRYEDTWAALHRRAKDCASAGELVDSEVVMLSAHWEKKKTSLVELQEQ
. 110 120 130 140 150 160 170 180 190 200
LQQLPALIADLESMTANLTHLEASFEEVENNLLHLEDLCGQCELERCKHMQSQQLENYKKNKRKELETFKAELDAEHAQKVLEMEHTQQMKLKERQKFFE
. 210 220 230 240 250 260 270 280 290 300
EAFQQDMEQYLSTGYLQIAERREPIGSMSSMEVNVDMLEQMDLMDISDQEALDVFLNSGGEENTVLSPALGPESSTCQNEITLQVPNPSELRAKPPSSSS
. 310 320 330 340 350
TCTDSATRDISEGGESPVVQSDEEEVQVDTALATSHTDREATPDGGEDSDS

Figure S18. NPAS3: (a) Predictions of condensation, aggregation, amyloid and prion prone regions (b) visualization of regions in 3D protein structure.

(a)

> NPAS3, Q8IXF0

MAPTKPSFQQDPSRRERITAQHPLPNQSECRKIYRYDGIYCESTYQNLQALRKEKSRDAARSRRGKENFEFYELAKLLPLPAAITSQLDKASII
RLTISYLKMRDFANQGDPPWNLRMEGPPPNTSVKVIGAQRRRSPSALAIEVFEAHLGSHILQSLDGFVFALNQEGKFLYISETVSIYLGLSQV
ELTGSSVFDYVHPGDHVEMAEQLGMKLPPGRGLLSQGTAEDGASSASSSSQSETPEPVESTSPSLLTTDNTLERSFFIRMKSTLTKRGVHIKS
SGYKVIHITGRLRLRVSLSHGRTVPSQIMGLVVVAHALPPPTINEVRIDCHMFVTRVNMDLNIIYCENRISDYMDLTPVDIVGKRCYHFIHAE
DVEGIRHSHLDLLNKGQCVTKYYRWMQKNGGYIWIQSSATIAINAKNANEKNIIWVNYLLSNPEYKDTPMDIAQLPHLPEKTSESSETSDS
ESDSKDTSGITEDNENSKSDEKGNQSENSEDPEPDRKKSGNACDNDMNCNDDGHSSSNPDSRDSDDSFEHSDFENPKAGEDGFGALGA
MQIKVERYVESESDLRLQNCESLTSDSAKDSDSAGEAGAQASSKHQKRKKRRKRQKGGSASRRRLSSASSPGGLDAGLVEPPRLLSSPNSA
SVLKIKTEISEPINFDNDSSIWNYPPNREISRNESPYSMTKPPSSEHFPSPQGGGGGGGGGGGLHVAIPDSVLTPPGADGAAARKTQFGAS
ATAALAPVASDPLSPPLSASPRDKHPGNGGGGGGGGGGAGGGGPSASNSLLYTGDLEALQRLQAGNVVLPLVHRVTGTLAATSTAAQRV
YTTGTIRYAPAEVTLAMQSNLLPNAHAVNFVDVNSPGFGLDPKTPMEMLYHHVHRLNMSGPFGGAVSAASLTQMPAGNVFTTAEGLFS
TLPFPVYSNGIHAAQTLERKED

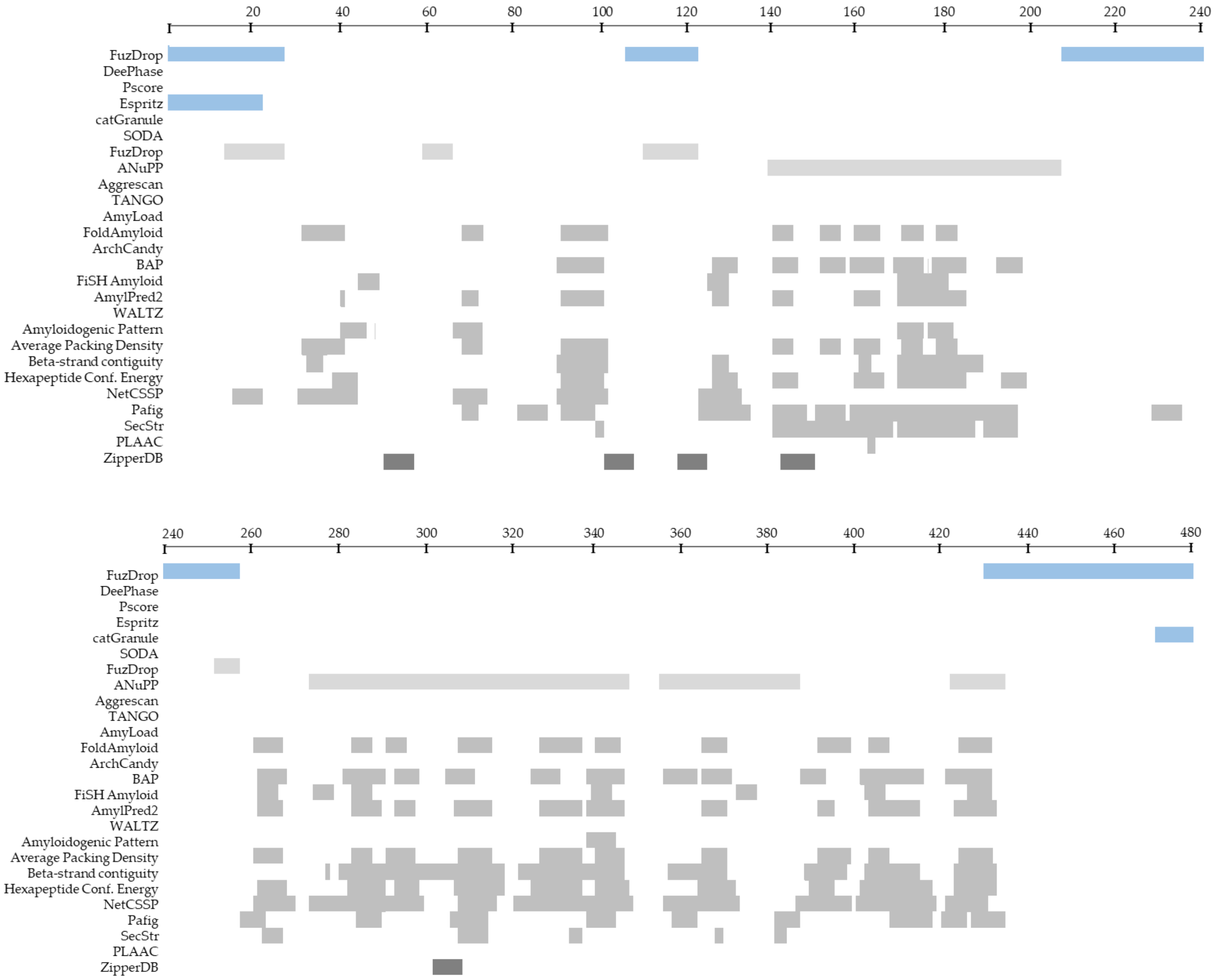

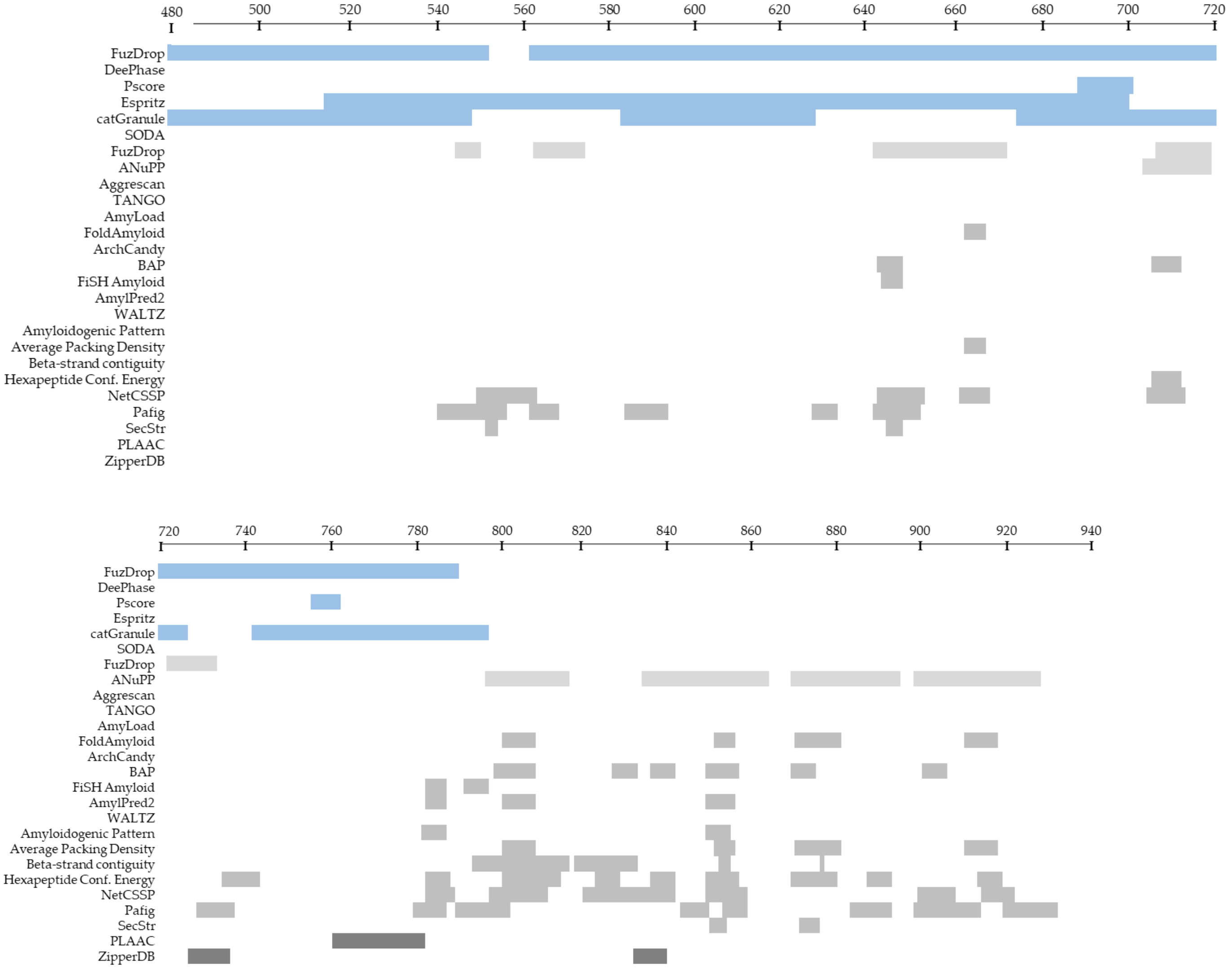
480
500
520
540
560
580
600
620
640
660
680
700
720
FuzDrop
DeePhase
Pscore
Espritz
catGranule
SODA
FuzDrop
ANuPP
Aggrescan
TANGO
AmyLoad
FoldAmyloid
ArchCandy
BAP
FiSH Amyloid
AmylPred2
WALTZ
Amyloidogenic Pattern
Average Packing Density
Beta-strand contiguity
Hexapeptide Conf. Energy
NetCSSP
Pafig
SecStr
PLAAC
ZipperDB
720
740
760
780
800
820
840
860
880
900
920
940

(b) **AF: Q8IXF0-F1**

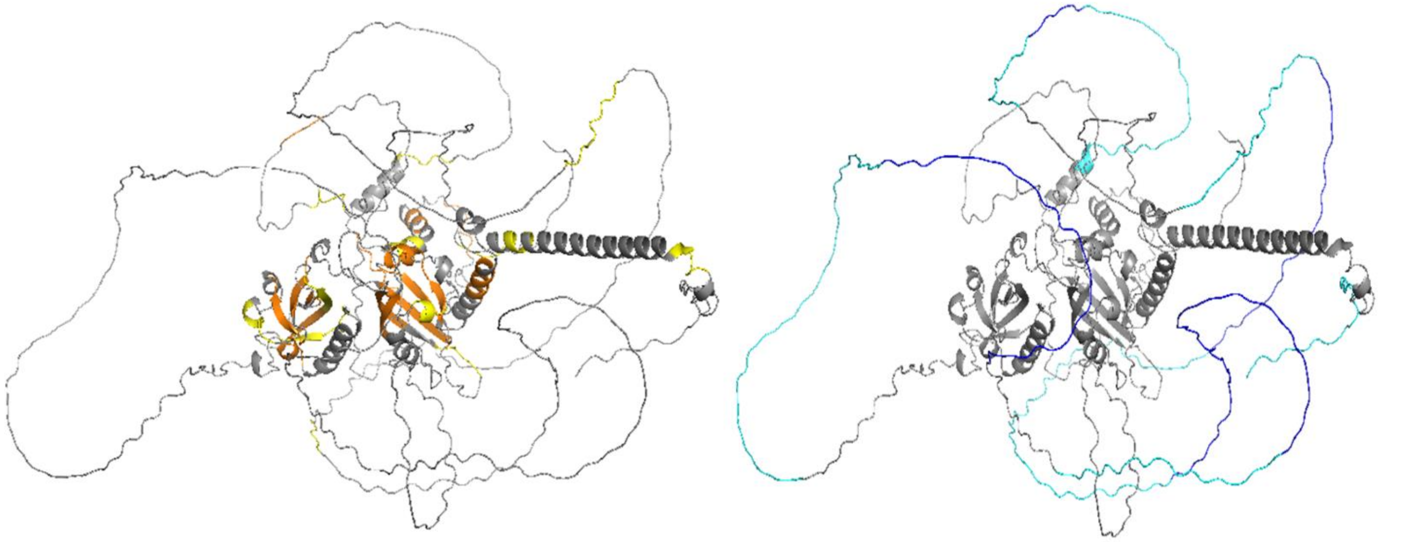

Condensation prone regions

1 10 20 30 40 50 60 70 80 90 100
MAPTKPSFQQDPSRRERITAQHPLPNQSECRKIYRYDGIYCESTYQNLQALRKEKSRDAARSRRGKENFEFYELAKLLPLPAAITSQLDKASIIRLTISY
. 110 120 130 140 150 160 170 180 190 200
LKMRDFANQGDPPWNLRMEGPPPNTSVKVIGAQRRRSPSALAIEVFEAHLGSHILQSLDGFVFALNQEGKFLYISETVSIYLGLSQVELTGSSVFDYVHP
. 210 220 230 240 250 260 270 280 290 300
GDHVEMAEQLGMKLPPGRGLLSQGTAEDGASSASSSSQSETPEPVESTSPSLLTTDNTLERSFFIRMKSTLTKRGVHIKSSGYKVIHITGRLRLRVSLSH
. 310 320 330 340 350 360 370 380 390 400
GRTVPSQIMGLVVVAHALPPPTINEVRIDCHMFVTRVNMDLNIIYCENRISDYMDLTPVDIVGKRCYHFIHAEDVEGIRHSHLDLLNKGQCVTKYYRWMQ
. 410 420 430 440 450 460 470 480 490 500
KNGGYIWIQSSATIAINAKNANEKNIIWVNYLLSNPEYKDTPMDIAQLPHLPEKTSESSETSDSESDSKDTSGITEDNENSKSDEKGNQSENSEDPEPDR
. 510 520 530 540 550 560 570 580 590 600
KKSGNACDNDMNCNDDGHSSSNPDSRDSDDSFEHSDFENPKAGEDGFGALGAMQIKVERYVESESDLRLQNCESLTSDSAKDSDSAGEAGAQASSKHQKR
. 610 620 630 640 650 660 670 680 690 700
KKRRKRQKGGSASRRRLSSASSPGGLDAGLVEPPRLLSSPNSASVLKIKTEISEPINFDNDSSIWNYPPNREISRNESPYSMTKPPSSEHFPSPQGGGGG .
. 710 720 730 740 750 760 770 780 790 800
GGGGGGLHVAIPDSVLTPPGADGAAARKTQFGASATAALAPVASDPLSPPLSASPRDKHPGNGGGGGGGGGGAGGGGPSASNSLLYTGDLEALQRLQAGN
. 810 820 830 840 850 860 870 880 890 900
VVLPLVHRVTGTLAATSTAAQRVYTTGTIRYAPAEVTLAMQSNLLPNAHAVNFVDVNSPGFGLDPKTPMEMLYHHVHRLNMSGPFGGAVSAASLTQMPAG
. 910 920 930
NVFTTAEGLFSTLPFPVYSNGIHAAQTLERKED

Aggregation/amyloid prone regions

1 10 20 30 40 50 60 70 80 90 100
MAPTKPSFQQDPSRRERITAQHPLPNQSECRKIYRYDGIYCESTYQNLQALRKEKSRDAARSRRGKENFEFYELAKLLPLPAAITSQLDKASIIRLTISY
. 110 120 130 140 150 160 170 180 190 200
LKMRDFANQGDPPWNLRMEGPPPNTSVKVIGAQRRRSPSALAIEVFEAHLGSHILQSLDGFVFALNQEGKFLYISETVSIYLGLSQVELTGSSVFDYVHP
. 210 220 230 240 250 260 270 280 290 300
GDHVEMAEQLGMKLPPGRGLLSQGTAEDGASSASSSSQSETPEPVESTSPSLLTTDNTLERSFFIRMKSTLTKRGVHIKSSGYKVIHITGRLRLRVSLSH
. 310 320 330 340 350 360 370 380 390 400
GRTVPSQIMGLVVVAHALPPPTINEVRIDCHMFVTRVNMDLNIIYCENRISDYMDLTPVDIVGKRCYHFIHAEDVEGIRHSHLDLLNKGQCVTKYYRWMQ
. 410 420 430 440 450 460 470 480 490 500
KNGGYIWIQSSATIAINAKNANEKNIIWVNYLLSNPEYKDTPMDIAQLPHLPEKTSESSETSDSESDSKDTSGITEDNENSKSDEKGNQSENSEDPEPDR
. 510 520 530 540 550 560 570 580 590 600
KKSGNACDNDMNCNDDGHSSSNPDSRDSDDSFEHSDFENPKAGEDGFGALGAMQIKVERYVESESDLRLQNCESLTSDSAKDSDSAGEAGAQASSKHQKR
. 610 620 630 640 650 660 670 680 690 700
KKRRKRQKGGSASRRRLSSASSPGGLDAGLVEPPRLLSSPNSASVLKIKTEISEPINFDNDSSIWNYPPNREISRNESPYSMTKPPSSEHFPSPQGGGGG .
. 710 720 730 740 750 760 770 780 790 800
GGGGGGLHVAIPDSVLTPPGADGAAARKTQFGASATAALAPVASDPLSPPLSASPRDKHPGNGGGGGGGGGGAGGGGPSASNSLLYTGDLEALQRLQAGN
. 810 820 830 840 850 860 870 880 890 900
VVLPLVHRVTGTLAATSTAAQRVYTTGTIRYAPAEVTLAMQSNLLPNAHAVNFVDVNSPGFGLDPKTPMEMLYHHVHRLNMSGPFGGAVSAASLTQMPAG
. 910 920 930
NVFTTAEGLFSTLPFPVYSNGIHAAQTLERKED

Figure S19. PRICKLE1: (a) Predictions of condensation, aggregation, amyloid and prion prone regions (b) visualization of regions in 3D protein structure.

(a)

> PRICKLE1, Q96MT3

MPLEMEPKMSKLAFGCQRSSTSDDDSGCALEEYAWVPPGLRPEQIQLYFACLPEEKVPYVNSPGEKHRIKQLLYQLPPHDNEVRYCQSLSE
EEKKELQVFSAQRKKEALGRGTIKLLSRAVMHAVCEQCGLKINGGEVAVFASRAGPGVCWHPSCFVCFTCNELLVDLIYFYQDGKIHCGRH
HAELLKPRCSACDEIIFADECTEAEGRHWHMKHFCCLECETVLGGQRYIMKDGRPFCCGCFESLYAEYCETCGEHIGVDHAQMTYDGQH
WHATEACFSCAQCKASLLGCPFLPKQGQIYCSKTCSLGEDVHASDSSDSAFQSARSRDSRRSVRMGKSSRSADQCRQSLLLSPALNYKFPG
LSGNADDTLSRKLDDLSLSRQGTSFASEEFWKGRVEQETPEDPEEWADHEDYMTQLLLKFGDKSLFQPQPNEMDIRASEHWISDNMVKS
KTELKQNNQSLASKKYQSDMYWAQSQDGLGDSAYGSHPGPASSRRLQELELDHGASGYNHDETQWYEDSLECLSDLKPEQSVRDSMDS
LALSNITGASVDGENKPRPSLYSLQNFEEMETEDCEKMSNMGTLNSSMLHRSAESLKSLSSELCPEKILPEEKPVHLPVLRRSKSQSRPQQV
KFSDDVIDNGNYDIEIRQPPMSERTRRRVYNFEERGSRSHHHRRRRSRKSRSDNALNLVTERKYSPKDRLRLYTPDNYEKFIQNKSAREIQAY
IQNADLYGQYAHATSDYGLQNPGMNRFLGLYGEDDDSWCSSSSSSSDSEEEGYFLGQPIPQPRPQRFAYYTDDLSSPPSALPTPQFGQRTT
KSKKKKGHKGKNCIIS

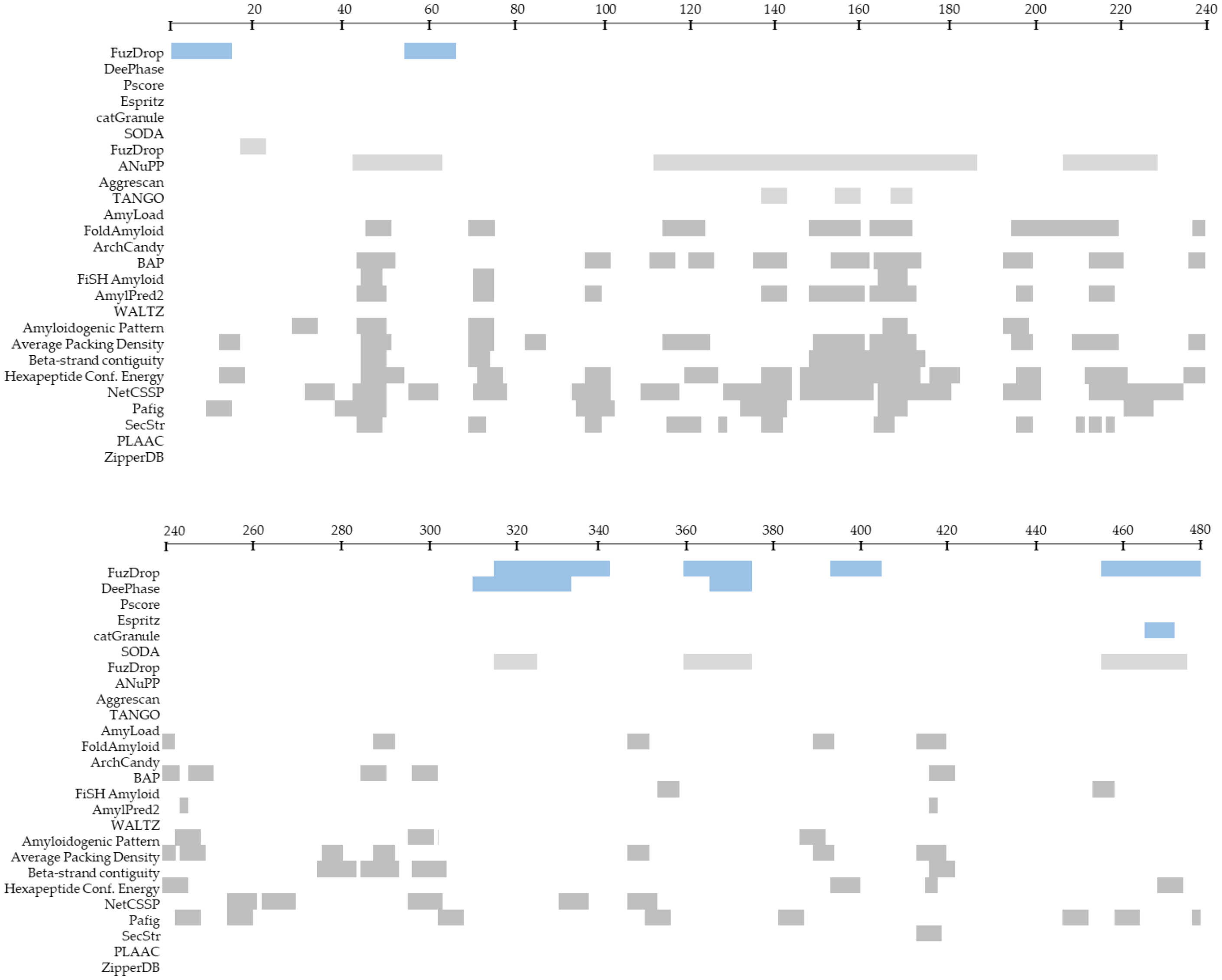

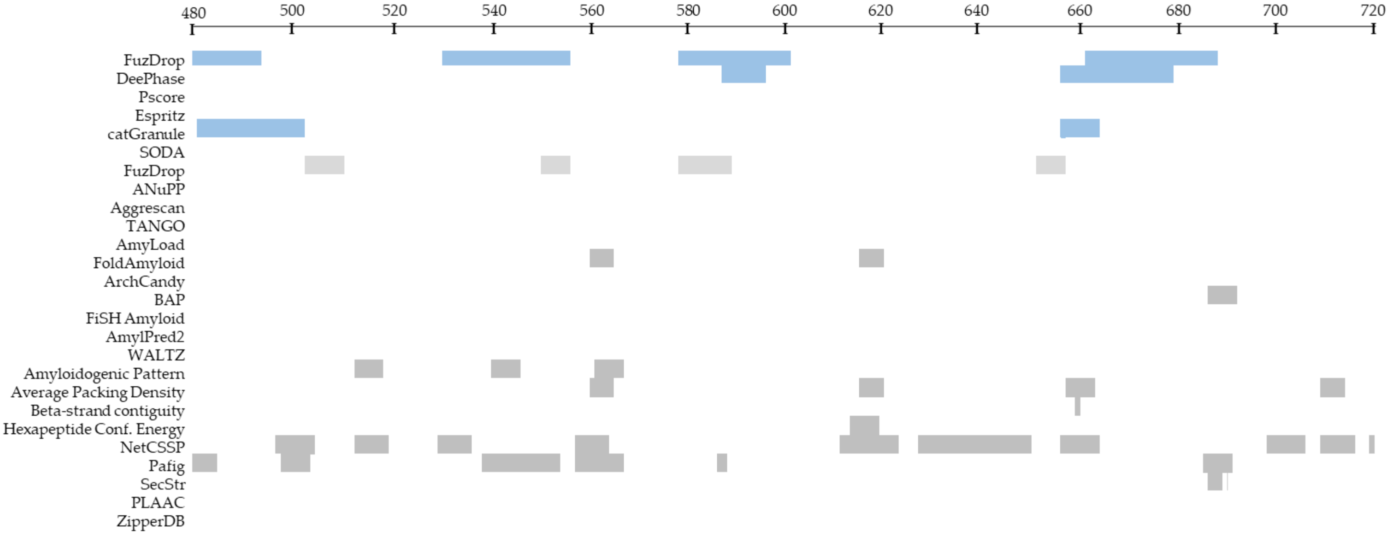
480
500
520
540
560
580
600
620
640
660
680
700
720
FuzDrop
DeePhase
Pscore
Espritz
catGranule
SODA
FuzDrop
ANuPP
Aggrescan
TANGO
AmyLoad
FoldAmyloid
ArchCandy
BAP
FiSH Amyloid
AmylPred2
WALTZ
Amyloidogenic Pattern
Average Packing Density
Beta-strand contiguity
Hexapeptide Conf. Energy
NetCSSP
Pafig
SecStr
PLAAC
ZipperDB

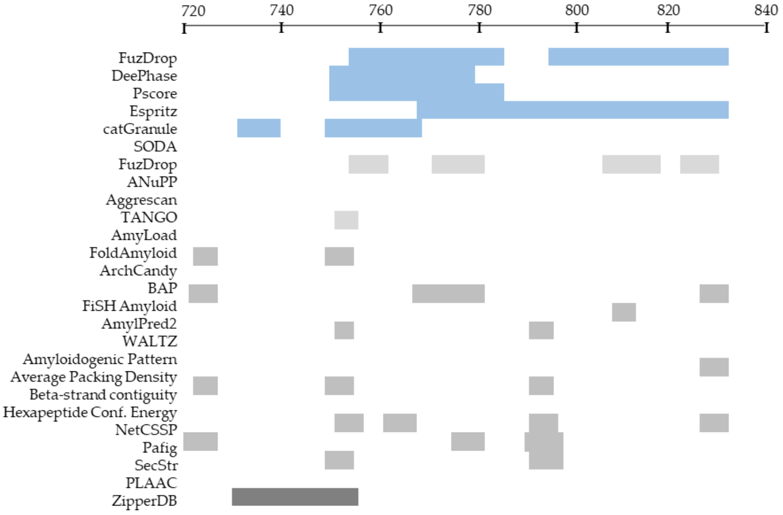
720
740
760
780
800
820
840
FuzDrop
DeePhase
Pscore
Espritz
catGranule
SODA
FuzDrop
ANuPP
Aggrescan
TANGO
AmyLoad
FoldAmyloid
ArchCandy
BAP
FiSH Amyloid
AmylPred2
WALTZ
Amyloidogenic Pattern
Average Packing Density
Beta-strand contiguity
Hexapeptide Conf. Energy
NetCSSP
Pafig
SecStr
PLAAC
ZipperDB

(b) AF-Q96MT3-F1

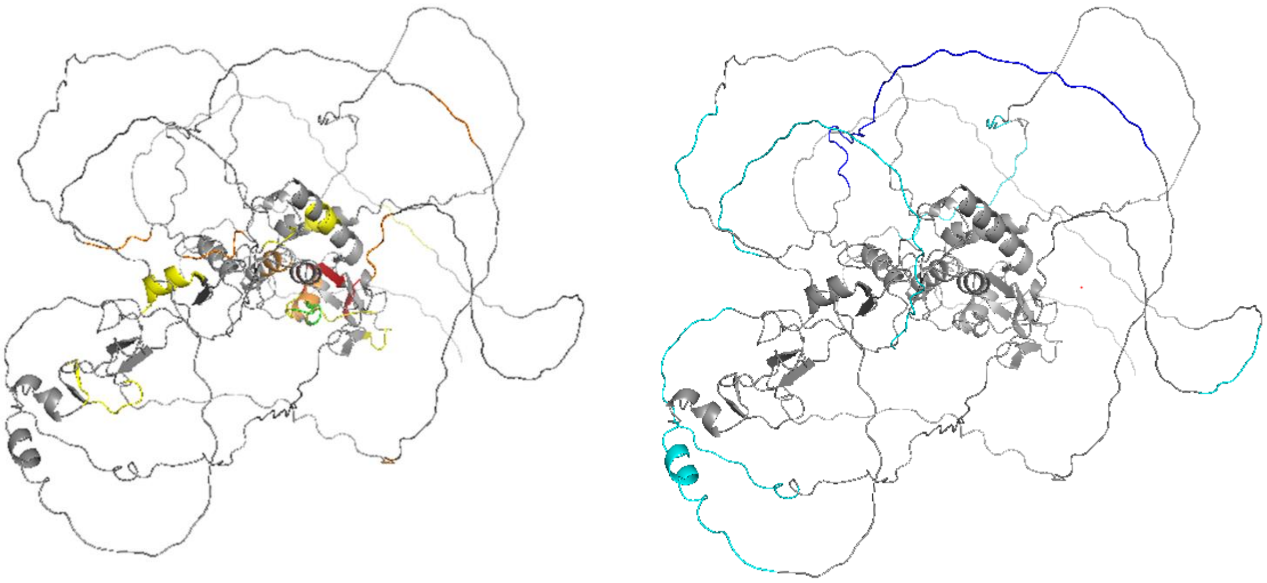

Condensation prone regions

1 10 20 30 40 50 60 70 80 90 100
MPLEMEPKMSKLAFGCQRSSTSDDDSGCALEEYAWVPPGLRPEQIQLYFACLPEEKVPYVNSPGEKHRIKQLLYQLPPHDNEVRYCQSLSEEEKKELQVF
. 110 120 130 140 150 160 170 180 190 200
SAQRKKEALGRGTIKLLSRAVMHAVCEQCGLKINGGEVAVFASRAGPGVCWHPSCFVCFTCNELLVDLIYFYQDGKIHCGRHHAELLKPRCSACDEIIFA
. 210 220 230 240 250 260 270 280 290 300
DECTEAEGRHWHMKHFCCLECETVLGGQRYIMKDGRPFCCGCFESLYAEYCETCGEHIGVDHAQMTYDGQHWHATEACFSCAQCKASLLGCPFLPKQGQI
. 310 320 330 340 350 360 370 380 390 400
YCSKTCSLGEDVHASDSSDSAFQSARSRDSRRSVRMGKSSRSADQCRQSLLLSPALNYKFPGLSGNADDTLSRKLDDLSLSRQGTSFASEEFWKGRVEQE
. 410 420 430 440 450 460 470 480 490 500
TPEDPEEWADHEDYMTQLLLKFGDKSLFQPQPNEMDIRASEHWISDNMVKSKTELKQNNQSLASKKYQSDMYWAQSQDGLGDSAYGSHPGPASSRRLQEL
. 510 520 530 540 550 560 570 580 590 600
ELDHGASGYNHDETQWYEDSLECLSDLKPEQSVRDSMDSLALSNITGASVDGENKPRPSLYSLQNFEEMETEDCEKMSNMGTLNSSMLHRSAESLKSLSS
. 610 620 630 640 650 660 670 680 690 700
ELCPEKILPEEKPVHLPVLRRSKSQSRPQQVKFSDDVIDNGNYDIEIRQPPMSERTRRRVYNFEERGSRSHHHRRRRSRKSRSDNALNLVTERKYSPKDR
. 710 720 730 740 750 760 770 780 790 800
LRLYTPDNYEKFIQNKSAREIQAYIQNADLYGQYAHATSDYGLQNPGMNRFLGLYGEDDDSWCSSSSSSSDSEEEGYFLGQPIPQPRPQRFAYYTDDLSS
. 810 820 830
PPSALPTPQFGQRTTKSKKKKGHKGKNCIIS

Aggregation/amyloid prone regions

1 10 20 30 40 50 60 70 80 90 100
MPLEMEPKMSKLAFGCQRSSTSDDDSGCALEEYAWVPPGLRPEQIQLYFACLPEEKVPYVNSPGEKHRIKQLLYQLPPHDNEVRYCQSLSEEEKKELQVF
. 110 120 130 140 150 160 170 180 190 200
SAQRKKEALGRGTIKLLSRAVMHAVCEQCGLKINGGEVAVFASRAGPGVCWHPSCFVCFTCNELLVDLIYFYQDGKIHCGRHHAELLKPRCSACDEIIFA
. 210 220 230 240 250 260 270 280 290 300
DECTEAEGRHWHMKHFCCLECETVLGGQRYIMKDGRPFCCGCFESLYAEYCETCGEHIGVDHAQMTYDGQHWHATEACFSCAQCKASLLGCPFLPKQGQI
. 310 320 330 340 350 360 370 380 390 400
YCSKTCSLGEDVHASDSSDSAFQSARSRDSRRSVRMGKSSRSADQCRQSLLLSPALNYKFPGLSGNADDTLSRKLDDLSLSRQGTSFASEEFWKGRVEQE
. 410 420 430 440 450 460 470 480 490 500
TPEDPEEWADHEDYMTQLLLKFGDKSLFQPQPNEMDIRASEHWISDNMVKSKTELKQNNQSLASKKYQSDMYWAQSQDGLGDSAYGSHPGPASSRRLQEL
. 510 520 530 540 550 560 570 580 590 600
ELDHGASGYNHDETQWYEDSLECLSDLKPEQSVRDSMDSLALSNITGASVDGENKPRPSLYSLQNFEEMETEDCEKMSNMGTLNSSMLHRSAESLKSLSS
. 610 620 630 640 650 660 670 680 690 700
ELCPEKILPEEKPVHLPVLRRSKSQSRPQQVKFSDDVIDNGNYDIEIRQPPMSERTRRRVYNFEERGSRSHHHRRRRSRKSRSDNALNLVTERKYSPKDR
. 710 720 730 740 750 760 770 780 790 800
LRLYTPDNYEKFIQNKSAREIQAYIQNADLYGQYAHATSDYGLQNPGMNRFLGLYGEDDDSWCSSSSSSSDSEEEGYFLGQPIPQPRPQRFAYYTDDLSS
. 810 820 830
PPSALPTPQFGQRTTKSKKKKGHKGKNCIIS

Figure S20. hnRNPA1: (a) Predictions of condensation, aggregation, amyloid and prion prone regions (b) visualization of regions in 3D protein structure.

(a)

> hnRNPA1, P09651

MSKSESPKEPEQLRKLFIGGLSFETTDESLRSHFEQWGTLTDCVVMRDPNTKRSRGFGFVTYATVEEVDAAMNARPHKVDGRVVEPKRAV
SREDSQRPGAHLTVKKIFVGGIKEDTEEHHLRDYFEQYGKIEVIEIMTDRGSGKKRGFAFVTFDDHDSVDKIVIQKYHTVNGHNCEVRKALS
KQEMASASSSQRGRSGSGNFGGGRGGGFGGNDNFGRGGNFSGRGGFGGSRGGGGYGGSGDGYNGFGNDGGYGGGGPGYSGGSRG
YGSGGQGYGNQGSGYGGSGSYDSYNNGGGGGFGGGSGSNFGGGGSYNDFGNYNNQSSNFGPMKGGNFGGRSSGPYGGGGQYFAK
PRNQGGYGGSSSSSSYGSGRRF

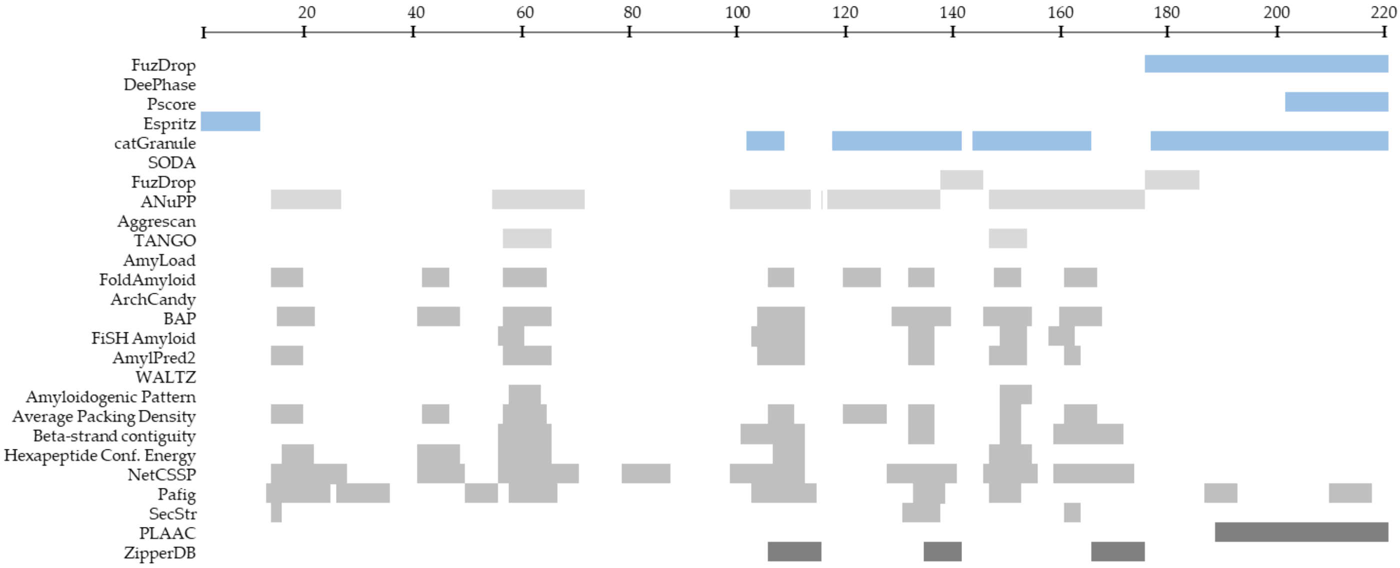

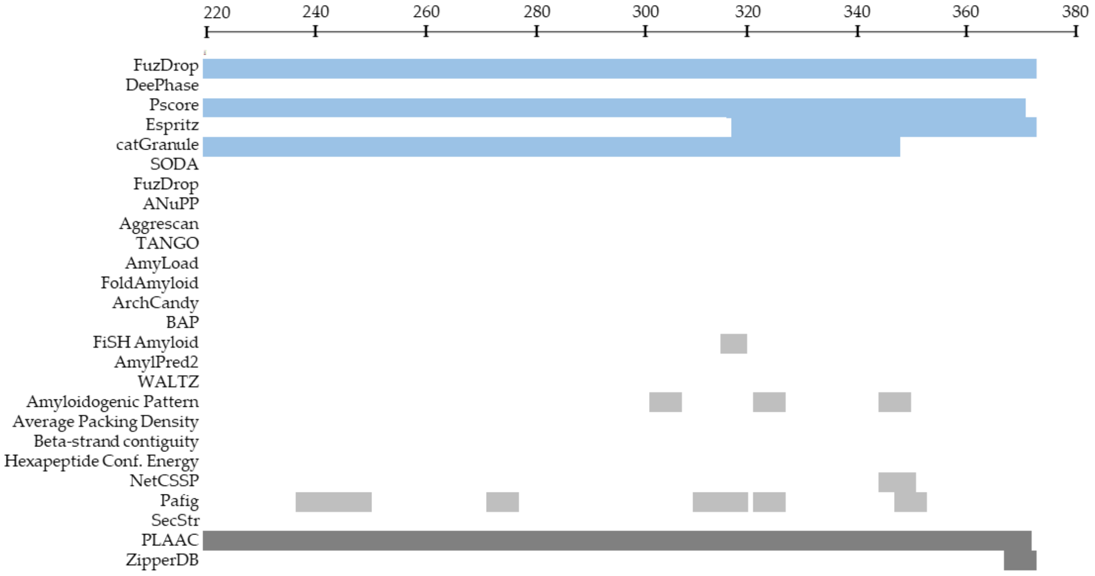

(b) AF-0000000210616471

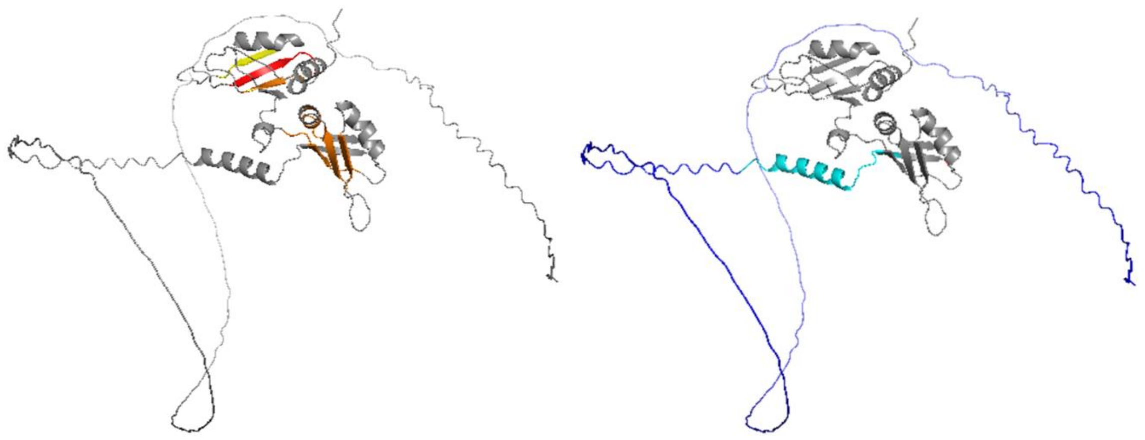

Condensation prone regions

1 10 20 30 40 50 60 70 80 90 100
MSKSESPKEPEQLRKLFIGGLSFETTDESLRSHFEQWGTLTDCVVMRDPNTKRSRGFGFVTYATVEEVDAAMNARPHKVDGRVVEPKRAVSREDSQRPGA
. 110 120 130 140 150 160 170 180 190 200
HLTVKKIFVGGIKEDTEEHHLRDYFEQYGKIEVIEIMTDRGSGKKRGFAFVTFDDHDSVDKIVIQKYHTVNGHNCEVRKALSKQEMASASSSQRGRSGSG
. 210 220 230 240 250 260 270 280 290
NFGGGRGGGFGGNDNFGRGGNFSGRGGFGGSRGGGGYGGSGDGYNGFGNDGGYGGGGPGYSGGSRGYGSGGQGYGNQGSGYGGSGSYDSY
. 300 310 320 330 340 350 360 370
NNGGGGGFGGGSGSNFGGGGSYNDFGNYNNQSSNFGPMKGGNFGGRSSGPYGGGGQYFAKPRNQGGYGGSSSSSSYGSGRRF

Aggregation/amyloid prone regions

1 10 20 30 40 50 60 70 80 90 100
MSKSESPKEPEQLRKLFIGGLSFETTDESLRSHFEQWGTLTDCVVMRDPNTKRSRGFGFVTYATVEEVDAAMNARPHKVDGRVVEPKRAVSREDSQRPGA
. 110 120 130 140 150 160 170 180 190 200
HLTVKKIFVGGIKEDTEEHHLRDYFEQYGKIEVIEIMTDRGSGKKRGFAFVTFDDHDSVDKIVIQKYHTVNGHNCEVRKALSKQEMASASSSQRGRSGSG
. 210 220 230 240 250 260 270 280 290
NFGGGRGGGFGGNDNFGRGGNFSGRGGFGGSRGGGGYGGSGDGYNGFGNDGGYGGGGPGYSGGSRGYGSGGQGYGNQGSGYGGSGSYDSY
. 300 310 320 330 340 350 360 370
NNGGGGGFGGGSGSNFGGGGSYNDFGNYNNQSSNFGPMKGGNFGGRSSGPYGGGGQYFAKPRNQGGYGGSSSSSSYGSGRRF

Table S1. Consensus of predictions of the condensation, aggregation, amyloid and prion -prone regions according to the number of the same region predictions by different predictors.

Protein	Condensation regions	Aggregation regions	Amyloid regions	Prion regions
stefin-A	1-11*, 53-59*, 64-73*	66-72*	16-20*, 41-57**, 65-70**, 81-85**	no
stefin-B	1-12*	37-42**, 43-52*, 54-58**, 66-72**	47-58**, 64-72**, 81-85*	no
cystatin-C	25-40*, 44-49*	9-20**, 82-90**, 122-130**	9-20**, 56-60*, 73-77**, 83-90**, 124-130**	no
α-synuclein	50-65**, 101-113**, 114-128*, 25-128*	14-19*, 36-42**, 55-63**, 73-80*	36-42**, 54-65**, 73-80*	no
SOD1	28-104*	30-38,* 101-107*, 114-121*, 146-152**	4-8*, 15-22*, 30-35*, 44-50*, 101-107**, 112-120**, 146-152**	no
malin	133-182*	44-48*, 49-59**, 86-95*, 121-128*, 137-134*, 183-187*, 198-211**, 212-217*, 241-247*, 266-279**, 283-325*, 336-348*, 366-372*, 380-395*	24-28**, 48-58**, 68-73*, 86-92**, 111-118*, 130-134*, 139-146*, 151-156*, 178-186**, 199-213**, 227-232*, 239-244*, 249-253*, 266-279**, 310-316**, 323-327**, 331-338**, 367-377**, 376-382**290, 295*, 301-305*, 387-392*	no
laforin	93-105*	119-124*, 144-152*, 156-161*, 180-191*, 226-230*, 248-256**, 257-268*, 275-287**, 288-298*	82-88**, 119-126**, 145-150**, 163-167*, 175-179*, 185-192**, 218-222*, 226-230**, 249-254**, 259-267**, 278-285**, 291-298**	no
RIP3	160-187*, 329-333*, 400-450*, 434-447**, 476-487*	46-54*, 120-131**, 202-217**	1-6*, 34-38*, 47-52**, 70-75**, 120-131**, 137-141*, 189-194**, 204-216**, 228-232**, 262-266**, 297-301*, 344-348*, 432-436*, 449-454**, 461-466*	no
CRMP1	510-567*, 525-547**	33-38*, 45-51*, 98-109*, 113-121*, 136-144**, 165-172**, 181-198**, 235-265*, 429-434*, 444-450**	31-37**, 47-51**, 62-66*, 72-76*, 101-107**, 137-146**, 152-157*, 163-169**,182-189**, 193-198**, 235-242**, 248-254**, 262-266*, 288-293*, 299-304**, 318-322*, 348-352*, 362-368**, 379-386**, 390-395**, 429-434*, 444-450**	no
TRIOBP1	20-30*, 140-214*, 187-200**, 280-295*, 350-363*, 397-420*	39-43*, 91-95*, 551-558*	12-16*, 39-43**, 82-86**, 91-95**, 105-109*, 171-175*, 487-491**, 510-514**, 552-558**, 574-579*	no
FUS	1-274**, 360-437*, 370-423**, 440-526**	283-312*, 370-375*	286-290**, 294-299*, 308-313*, 322-326**, 364-370*, 437-441*	49-53*, 81-88*, 112-119*
TAU	1-25*, 26-50**, 51-119*, 120-134**,135-146*,147-230**,231-300**, 231-300*,310-317**, 318-330*,331-349**, 350-455*, 456-525**,526-573*, 574-589**,590-605*, 606-611**,612-622*, 700-709*, 719-739*	186-195+, 265-278*, 613-620*	299-308**, 543-548*, 591-596**, 623-628**, 707-711*	no
TDP-43	255-269*, 270-310**, 311-344*, 345-414**	54-59*, 70-75**, 104-111*, 125-135*, 212-222*, 228-235**, 244-255*, 332-336**	3-7*, 27-35**, 54-60**, 70-76**, 105-111**, 129-135**, 148-154**, 171-175*, 192-196*, 218-222**, 228-234**, 248-257*, 313-318**, 335-339*	324-329*, 368-381*
DISC1	1-30**, 180-200*, 230-250*, 285-315**, 420-440*	54-59*, 205-217*, 403-411*, 624-633*, 751-757*	62-65**, 118-122*, 144-148*, 210-217**, 266-271*, 348-354**, 388-394**, 400-406*, 448-453*, 474-479**, 508-513*,	no

			557-562*, 599-604**, 628-632**, 685-691*, 699-705**, 823-829**, 844-850*	
LIMP2	no	10-26*, 440-457*	3-8**, 9-12*, 13-28**, 39-43*, 60-67**, 131-136*, 138-152**, 166-171**, 185-192**, 199-204**, 213-217**, 223-227**, 237-242**, 264-269**, 274-282**, 310-315*, 323-328**, 335-339*, 366-370**, 374-378**, 383-390**, 403-411**, 430-457**	no
PRICKLE1	317-334*, 367-376*, 468-474*, 481-493*, 588-596*, 657-679*, 750-785**, 795-831*	138-143*, 155-160*, 168-172*	45-50**, 71-75**, 97-102*, 120-124*, 138-143*, 149-164*, 165-172**, 196-200*, 214-219**, 238-248*, 289-293*, 298-302*, 417-421*, 561-565*, 616-620*, 722-726*, 750-754*, 791-795*	no
FABP3	no	83-87*, 114-119*	67-71*,82-86*,93-97*, 105-109*, 114-119**, 119-124*	no
DTNBP1	269-291, 323-327, 335-351	no	7-11*, 80-85**, 105-109*, 214-218*, 252-257**	no
hnRNPA1	177-201*, 202-370**	57-65*, 147-153*	14-19**, 42-46*, 57-65**, 104-112**, 132-136**, 148-152**, 161-166**	367-371
NPAS3	1-22*, 472-515*, 516-549**,563-583*,584-628**, 629-674*,675-701**, 702-726*, 742-755*, 763-790*	707-719*	33-37*, 69-73*, 92-101**, 127-132**, 141-145**, 152-156*, 160-165**, 170-183**, 193-197*, 262-267**,284-290**, 294-299*, 309-316**, 328-337**, 340-347**, 359-364*, 366-371**, 391-399*, 405-409**, 410-417*, 426-433**, 644-648*, 783-787*, 801-808**, 851-855**, 870-876*, 913-917*	no

* consensus of 2 predictions in condensation, aggregation and prion regions or consensus of 4-5 predictions in amyloid regions

** consensus of ≥ 3 predictions in condensation and aggregation regions or consensus of ≥ 6 predictions in amyloid regions
